# Multi-electron reactivity of a cofacial di-tin(ii) cryptand: partial reduction of sulfur and selenium and reversible generation of S_3_˙^–^ †
†Electronic supplementary information (ESI) available: Experimental procedures and crystallographic details. CCDC 1469593, 1469595 and 1469596. For ESI and crystallographic data in CIF or other electronic format see DOI: 10.1039/c6sc01754a



**DOI:** 10.1039/c6sc01754a

**Published:** 2016-07-06

**Authors:** Julia M. Stauber, Peter Müller, Yizhe Dai, Gang Wu, Daniel G. Nocera, Christopher C. Cummins

**Affiliations:** a Department of Chemistry , Massachusetts Institute of Technology , 77 Massachusetts Avenue , Cambridge , MA 02139-4307 , USA . Email: ccummins@mit.edu; b Department of Chemistry and Chemical Biology , Harvard University , 12 Oxford Street , Cambridge , MA 02138-2902 , USA . Email: dnocera@fas.harvard.edu; c Department of Chemistry , Queen's University , 90 Bader Lane , Kingston , Ontario , Canada K7L 3N6 . Email: gang.wu@chem.queensu.ca

## Abstract

Cofacial bimetallic tin(ii) ([Sn_2_(*m*BDCA-5t)]^2–^, **1**) and lead(ii) ([Pb_2_(*m*BDCA-5t)]^2–^, **2**) complexes have been prepared by hexadeprotonation of hexacarboxamide cryptand *m*BDCA-5t-H_6_ together with double Sn(ii) or Pb(ii) insertion. Reaction of **1** with elemental sulfur or selenium generates di-tin polychalcogenide complexes containing μ-E and bridging μ-E_5_ ligands where E = S or Se, and the Sn(ii) centers have both been oxidized to Sn(iv). Solution and solid-state UV-Vis spectra of [(μ-S_5_)Sn_2_(μ-S)(*m*BDCA-5t)]^2–^ (**4**) indicate that the complex acts reversibly as a source of S_3_˙^–^ in DMF solution with a *K*_eq_ = 0.012 ± 0.002. Reductive removal of all six chalcogen atoms is achieved through treatment of [(μ-E_5_)Sn_2_(μ-E)(*m*BDCA-5t)]^2–^ with PR_3_ (R = ^*t*^Bu, Ph, O^i^Pr) to produce six equiv. of the corresponding EPR_3_ compound with regeneration of di-tin(ii) cryptand complex **1**.

## Abstract 2

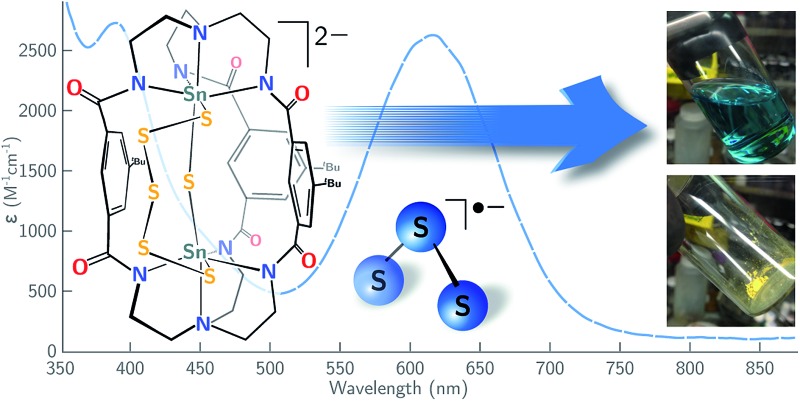
A cofacial di-tin(ii) hexacarboxamide cryptand that binds sulfur to form a complex containing μ-S and bridging μ-S_5_ ligands and acts reversibly as a source of S_3_˙^–^ in DMF solution is described.

## Introduction

Multi-electron transfer reactions are an area of intense interest due to their key role in both biological^1^ and synthetic systems for the activation and transformation of small molecules germane to energy conversion. These systems have the ability to accommodate and deliver multiple electrons to reaction substrates at one time, and typically feature two or more redox active metal ions confined within a single structural unit. Synthetic organometallic complexes that undergo multi-electron redox processes have been extensively studied for their applications in electrocatalysis,^2^ sensing,^3^ homogeneous,^4^ and heterogeneous^5^ catalysis. Many of these systems are based upon face-to-face diporphyrins, in which two metalloporphyrins are rigidly linked together in a cofacial arrangement.^6^ Complexes of this type have received considerable interest due to their ability to carry out multi-electron processes such as oxygen reduction,^7^ nitrogen reduction,^8^ and H_2_O_2_ disproportionation.^9^

We have shown previously that macro-bicyclic hexacarboxamide cryptand molecules,^10^ in hexa-deprotonated form, serve as excellent frameworks engendering cofacial transition-metal bimetallic systems with a range of intermetal distances;^10,11^ these systems are distinguished from bis-porphyrin constructs in that the metal ion coordination environment is trigonal rather than tetragonal. While transition metal complexes that carry out multi-electron reactions have, and continue to receive attention, functional p-block metal analogues have been far less studied.^12^ Recent work has shown that the chemistry of some heavy main group elements can resemble that of transition-metal complexes, and main-group metal systems have displayed small-molecule reactivity previously thought to be the exclusive domain of d-block elements.^13^

For the present work, in extending the approach we sought to access a pair of tin(ii)/tin(iv) redox couples giving the potential for four-electron transformations within the capsular cryptand environment. We chose to investigate redox reactions involving the group 16 elements, the ensuing finding that clean and informative reactivity was observed for both sulfur and selenium forming the basis of the present work. We note that both the reduction of oxygen to peroxide dianion, and the reduction of sulfur to sulfide ion, are currently targets for the development of new battery chemistries;^14^ fundamental studies such as the present one have the potential to reveal how the reduction processes may become controlled and selective in response to the utilization of a pre-organized architecture.

## Results and discussion

**Scheme 1:**
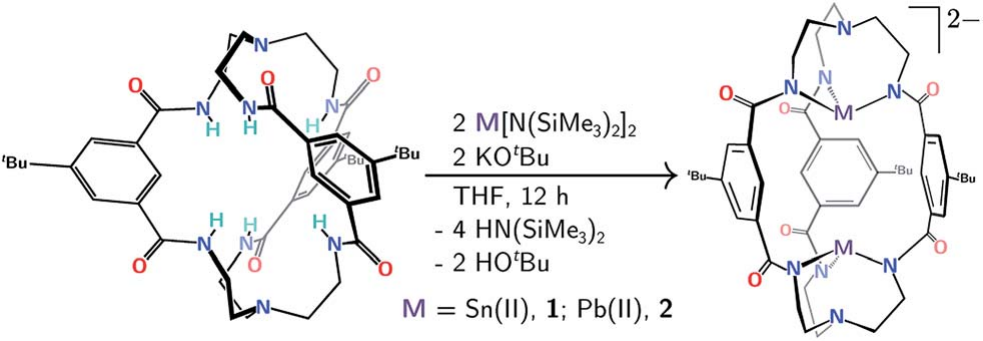
Protocol for double Sn(ii) or Pb(ii) insertion into the *m*BDCA-5t-H_6_ cryptand (together with hexadeprotonation) to generate [Sn_2_(*m*BDCA-5t)]^2–^ (**1**) and [Pb_2_(*m*BDCA-5t)]^2–^ (**2**).

Double insertion of tin(ii) or lead(ii) into the cryptand proceeds upon treatment of *m*BDCA-5t-H_6_ (ref. 15) with KO^*t*^Bu in the presence of Sn[N(SiMe_3_)_2_]_2_ or Pb[N(SiMe_3_)_2_]_2_ (ref. 16) in THF. This procedure affords the [K_2_(THF)][Sn_2_(*m*BDCA-5t)] ([K_2_(THF)][**1**]) and [K_2_(THF)][Pb_2_(*m*BDCA-5t)] ([K_2_(THF)][**2**]) salt complexes (Scheme 1) as colorless powders in 68, and 69% yield, respectively. In their ^1^H NMR spectra, both **1** (Fig. S1†) and **2** (Fig. S7†) display two resonances corresponding to the aryl protons of the three phenylene spacers, and four resonances are observed for the tren (tren = tris-2-aminoethylamine) methylene residues, suggesting that these bimetallic dianions retain their approximate *C*_3h_ symmetry in solution at room temperature. Complexes **1** and **2** display single resonances in their ^119^Sn{^1^H} NMR (*I* = 1/2, *δ* –381.6 ppm, DMSO-*d*_6_, Fig. 5c), and ^207^Pb NMR spectra (*I* = 1/2, *δ* 2641.4 ppm, DMSO-*d*_6_, Fig. S9†), similarly consistent with the presence of a horizontal mirror plane now relating the tin and lead ions in their *C*_3h_ geometry. The formulation of both dianions **1** and **2** has also been confirmed by ESI-MS (–) with *m*/*z* values of 541.12 (calc'd, 541.13, Fig. S4†), and 630.18 (calc'd, 630.21, Fig. S10†), respectively.

**Fig. 1:**
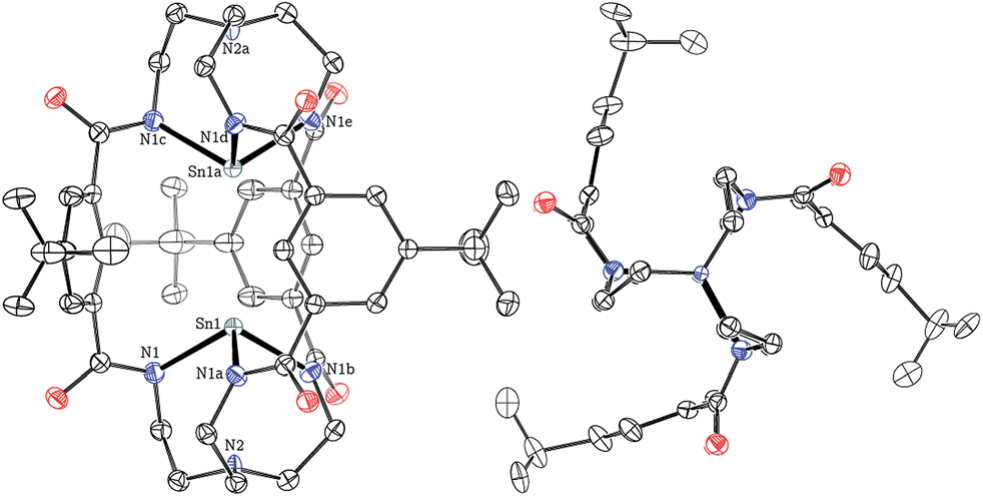
Solid-state structure of [Sn_2_(*m*BDCA-5t)]^2–^ (**1**) with thermal ellipsoids (drawn using PLATON^17^) shown at the 50% probability level and with [K(Kryptofix 2,2,2)]^+^ cations, disorder, and hydrogen atoms omitted for clarity. Selected interatomic distances (Å) and angles (°): Sn1–N1 3.116(3), Sn1–N2 2.2531(16), Sn1–Sn1a 3.5373(7), N1–Sn1–N1a 100.82(5).

Crystals of both **1** and **2** as their [K(Kryptofix 2,2,2)]^+^ salts were grown by vapor diffusion of Et_2_O into saturated DMF solutions over the course of 48 h at 23 °C. Single-crystal X-ray diffraction studies provided the structures of **1** and **2** shown in Fig. 1 and S51,† respectively. [K(Kryptofix 2,2,2)]_2_[**1**] and [K(Kryptofix 2,2,2)]_2_[**2**] are isostructural, crystallizing in the hexagonal space group *P*6_3_/*m*, with the bimetallic M···M cores lying on a crystallographic three-fold axis of rotation. Both anions **1** and **2** have effective *C*_3h_ point group symmetry with the metal centers adopting a trigonal pyramidal coordination environment (Fig. 1) as expected for triamidostannate(ii) systems.^18^ The solid-state structures of **1** and **2** reveal Sn···Sn and Pb···Pb intermetal separations of only 3.5373(7), 3.5274(5) Å, respectively, distances which are significantly shorter than the average M···M distances that span 6.080 to 6.495 Å for the bimetallic cryptand complexes of metal(ii) ions previously reported, where M = Mn, Fe, Co, Ni, Zn, and wherein the transition-metal ions adopt the trigonal monopyramidal coordination motif as they sink deeper into the tren-based N_3_N binding pockets.^11^

**Fig. 2:**
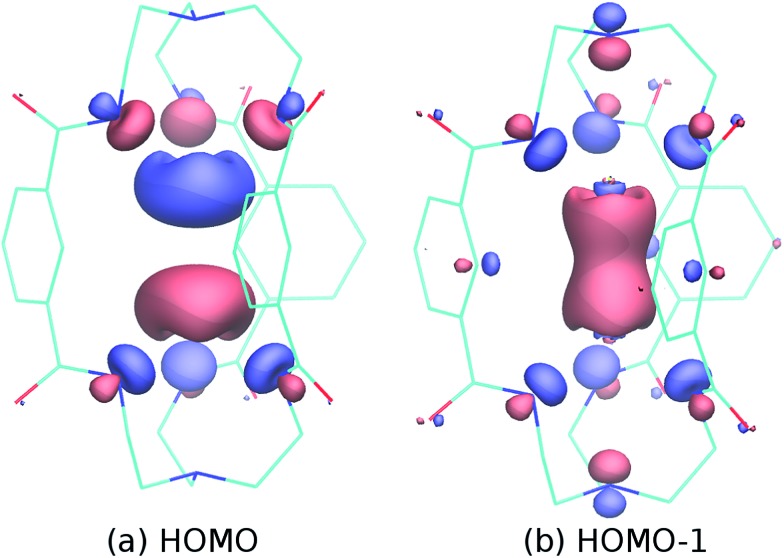
HOMO (a) and HOMO–1 (b) of the model dianion [Sn_2_(*m*BDCA-5H)]^2–^ based on the crystallographic coordinates of **1** and computed using ORCA^19^ at the double-hybrid level of DFT theory^20^ B2PLYP-D3/def2-TZVPP with corrections for dispersion^21^ and including scalar relativistic effects *via* the ZORA method.^22^

Being interested in the potential multi-electron redox reactivity of these p-block metal cryptates, we carried out computations of electronic structure for complex **1** (see Fig. 2 for details); the HOMO consists largely of an out-of-phase combination of the two tin lone pairs, while the HOMO–1 is an in-phase combination whose appearance nicely suggests that these metal ions are within bonding distance. The largely metal-centered nature of the HOMO and HOMO–1 electron pairs of **1** is fully in line with the expected metal-centered redox activity.

With access to salts of **1** and **2** on scales approaching 300 mg per synthesis, we were able to initiate exploratory reactivity studies involving the group 16 elements. While the di-lead(ii) complex **2** displayed no reaction with chalcogens, the di-tin(ii) cryptate **1** was found to react cleanly with both elemental sulfur and selenium. There are hundreds of fully characterized transition-metal cyclic polysulfide and polyselenide complexes known in the literature,^23^ however, significantly fewer examples of polychalcogenide main group metal complexes exist. Polychalcogenide complexes containing group 14 metal ions are especially rare,^24^ and there are only two structurally characterized examples we are aware of where a polysulfide or polyselenide ring (with *n* > 3 for S, or Se) bridges two group 14 centers.^25^ Even though tin(ii) amide compounds are known to react with sulfur and selenium to typically form thermodynamically favored bis-μ-chalcogenide products,^26,27^ we were interested to determine whether the preorganization offered by the *m*BDCA-5t cryptand framework would direct the system into new assemblages of atoms otherwise inaccessible in the absence of such a supramolecular construct.

**Scheme 2:**
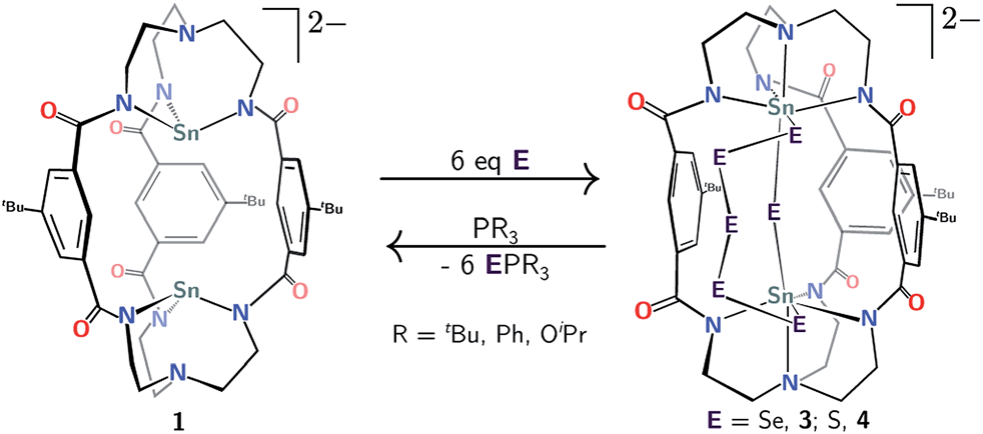
Reactivity of [Sn_2_(*m*BDCA-5t)]^2–^ (**1**) with elemental selenium and sulfur to generate [(μ-E_5_)Sn_2_(μ-E)(*m*BDCA-5t)]^2–^ (E = Se, **3**; S, **4**).

**Fig. 3:**
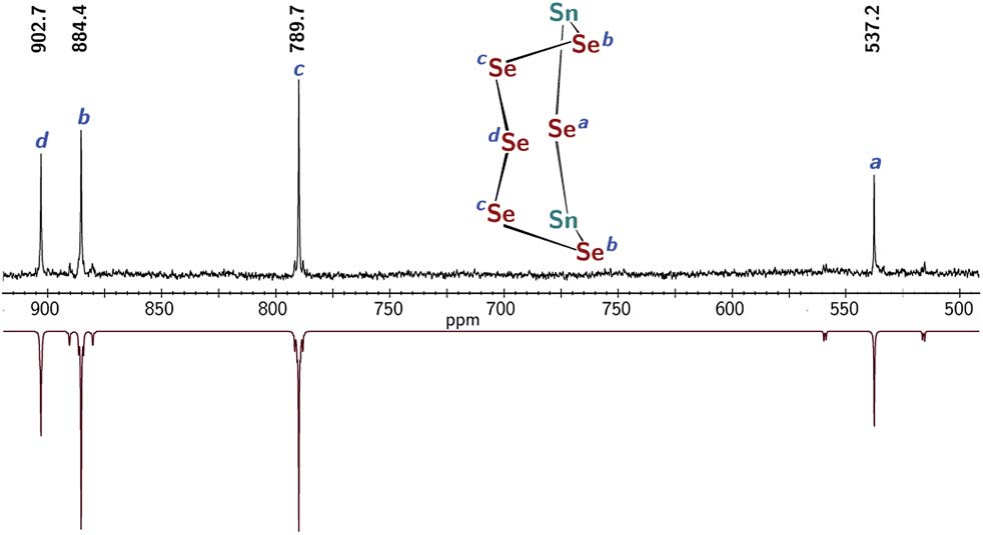
Experimental ^77^Se NMR spectrum of **3** (top, DMSO-*d*_6_, 76.3 MHz, 20 °C), and simulated spectrum (bottom) showing satellite peaks corresponding to the *J*_119_Sn_–77_Se__ coupling.

Treatment of **1** with elemental selenium (6 equiv.) in DMF solution was found to elicit a color change from colorless to deep red upon thorough mixing (Scheme 2). After workup, the selectively formed product of this reaction, [(μ-Se_5_)Sn_2_(μ-Se)(*m*BDCA-5t)]^2–^ (**3**), was isolated in 71% yield as a brick red solid. The ^1^H NMR spectrum of **3** (Fig. S12†) features three distinct *tert*-butyl resonances, each with integrations of nine protons, and six aryl resonances with integrations of one and two protons in a 1 : 1 ratio. The ^119^Sn NMR spectrum of **3** consists of one singlet (*δ* –876.5 ppm, Fig. 5a, ^1^ *J*_119_Sn_–77_Se__ = 3381 Hz), and the ^77^Se NMR spectrum contains four resonances that are attributed to the four distinct selenium environments of complex **3**. The resonances located at *δ* 537.2, and 884.4 ppm contain well-resolved satellite peaks corresponding to ^1^ *J*_119_Sn_–77_Se__ coupling of 3371 and 785 Hz, respectively (Fig. 3).^28^ While the ^1^H NMR spectrum revealed that the three arms of the cryptand are inequivalent, the ^119^Sn NMR data indicate that **3** contains only one tin environment, indicating that a lowering of symmetry from *C*_3h_ to *C*_s_ has occurred through the reaction of **1** with selenium.

**Fig. 4:**
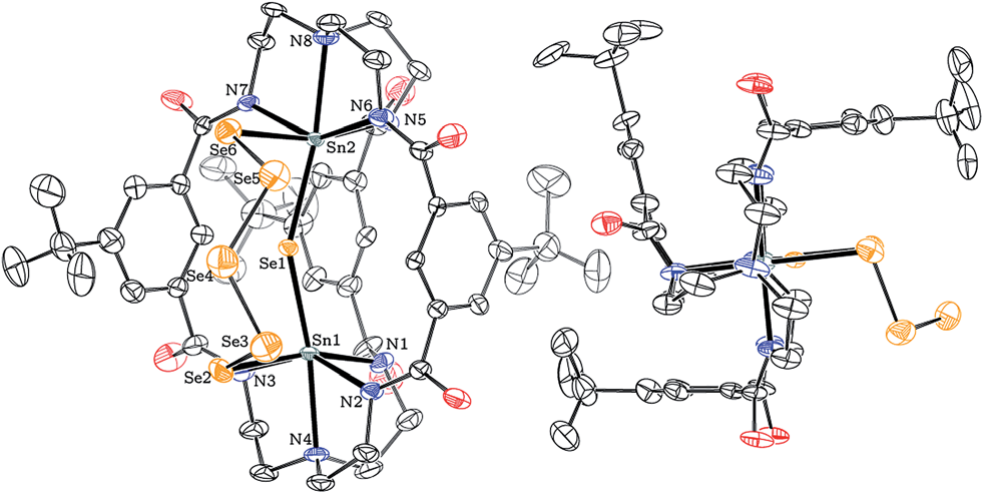
Solid-state structure of [(μ-Se_5_)Sn_2_(μ-Se)(*m*BDCA-5t)]^2–^ (**3**) with thermal ellipsoids (drawn using PLATON^17^) shown at the 50% probability level. K^+^ cations, DMF solvent molecules, disorder, and H-atoms omitted for clarity. Selected interatomic distances (Å) and angles (°): Sn1–Sn2 4.932(1), Sn1–Se1 2.578(2), Sn2–Se1 2.605(2), Sn1–Se2 2.726(3), Sn2–Se6 2.710(3), Sn1–N2 2.243(16), Sn1–N3 2.235(16), Sn1–N4 2.354(14), Sn2–N5 2.241(18), Sn2–N6 2.251(16), Sn2–N7 2.248(17), Sn2–N8 2.343(14), Sn1–Se1–Sn2 144.20(8), N3–Sn1–N1 86.8(18), N2–Sn1–N1 90.3(17), N3–Sn1–Se2 88.8(15), N2–Sn1–Se2 86.0(15), N5–Sn2–N7 90.1(15), N5–Sn2–N6 95.8(13), N7–Sn2–Se6 87.0(12), N6–Sn2–Se6 80.5(10).

A solid-state structure established the identity of this species as [K_2_(DMF)_3_][(μ-Se_5_)Sn_2_(μ-Se)(*m*BDCA-5t)] (**3**), and Fig. 4 shows a thermal ellipsoid plot of the dianion in this complex salt. X-ray quality dark red crystals of [K_2_(DMF)_3_][**3**] were grown by vapor diffusion of diethyl ether into a saturated DMF solution of the salt over the course of 12 h at 23 °C. The solid-state molecular structure of **3** reveals that the two Sn(iv) ions reside in a distorted octahedral environment, each coordinated to the three carboxamide nitrogen atoms from the cryptand as well as one μ-Se^2–^ ligand and one selenium from the five membered ^–^Se(Se)_3_Se^–^ pentaselenide chain that links the two tin(iv) centers. The observed molecular structure of dianion **3** provides the first glimpse of a conformation in which two cryptand arms are splayed apart in a manner that permits the pentaselenide chain to bridge the tin(iv) ions in between them, revealing a degree of flexibility we had not previously appreciated for this type of bimetallic hexacarboxamide ligand architecture. The selective formation of this product shows that elemental selenium reacts with complete consumption of all four reducing equivalents stored in the di-tin(ii) reservoir of dianion **1**, while the level of reduction of the elemental selenium, to Se^2–^ and Se_5_^2–^, is such that only one-third of the complete oxidizing power of 6 Se^0^ is quenched and four reducible Se–Se bonds remain.

The UV-Vis spectrum of **3** in DMF solution shows two major absorptions located at *λ*_max_ = 452 (*ε* = 4207 M^–1^ cm^–1^), and 585 (*ε* = 842 M^–1^ cm^–1^) nm (0.26 mM, Fig. S16†). Both observed bands are in good agreement with the literature reported absorption values for Se_6_^2–^ of *λ*_max_ = 440 and 598 nm in DMA solution.^29^

**Fig. 5:**
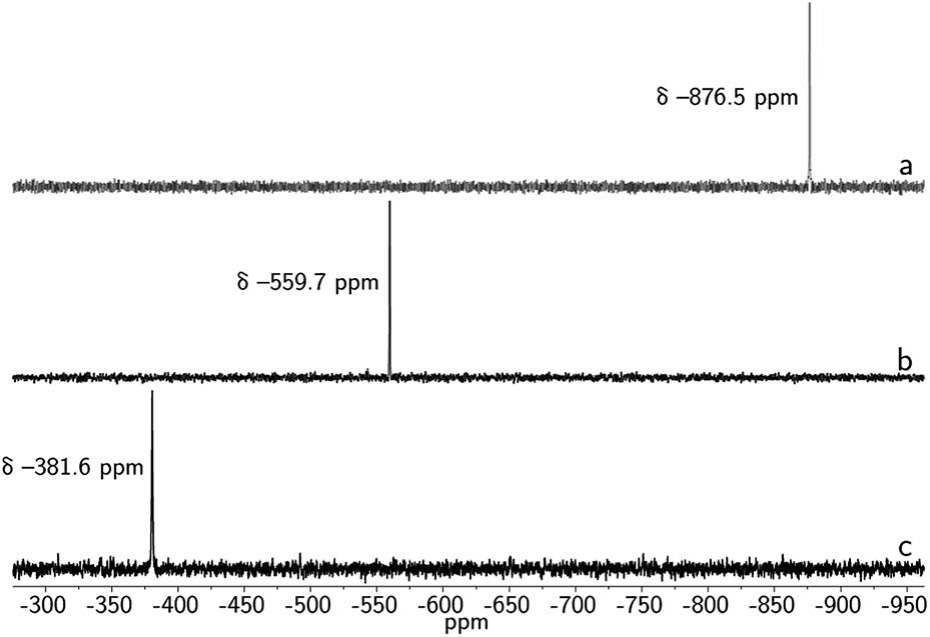
^119^Sn{^1^H} NMR spectra of **3** (a), **4** (b), and **1** (c) taken in DMSO-*d*_6_ at 20 °C (149 MHz).

Similarly, the reactivity of **1** with elemental sulfur was investigated. Treatment of **1** with 3/4 eq. S_8_ in DMF solution elicits a rapid color change from colorless to blue green. Addition of Et_2_O to the crude reaction mixture results in the precipitation of the [K_2_(DMF)_3_][(μ-S_5_)Sn_2_(μ-S)(*m*BDCA-5t)] complex (**4**, Scheme 2) as a bright yellow solid in 63% yield. Although suitable crystals were not obtained for an X-ray diffraction study, the structure of the sulfur-containing product can be deduced from its NMR spectra. The NMR properties of **4** are similar to those of **3**, with three distinct *tert*-butyl resonances and six aryl resonances in its ^1^H NMR spectrum (Fig. S18†), and one resonance in its ^119^Sn NMR spectrum (*δ* = –559.7 ppm, Fig. 5b), consistent with μ-S_5_ and μ-S ligands bridging two tin centers. The data again indicate that reaction of **1** with elemental sulfur results in oxidation of both Sn(ii) centers to Sn(iv) with concomitant partial reduction of sulfur by a total of four electrons.

Reactions of tin(ii) amide compounds with oxygen, sulfur, or selenium typically result in complexes containing terminal Sn=E bonds^30^ or thermodynamically favored bis-μ-chalcogenide products,^27^ with complete reduction to the oxide, sulfide, or selenide ion, E^2–^ (E = O, S, Se). In our system, however, the preorganization of the cryptand architecture has offered kinetic control of the products obtained such that the chalcogen reduction is incomplete and we have stopped at the level of two electrons for every three sulfur/selenium atoms. Such partial chalcogen reduction calls to mind results from Richeson *et al.* who reported the reaction of a mononuclear tin(ii) amide with elemental sulfur to provide a bidentate chelating S_4_^2–^ ligand.^31^

We note that treatment of **1** with sulfur-atom-transfer (SAT) reagents such as propylene sulfide and Ph_3_SbS^32^ led to no observable reaction. The five-membered chalcogen rings of **3** and **4** are favored over any other ring size, as treatment of **1** with >6 equiv. Se or S results in exclusive formation of **3** and **4**, and reaction of **1** with <6 equiv. Se or S results in incomplete conversion of **1** to **3** or **4**, respectively. The exclusive isolation of the [(μ-E_5_)Sn_2_(μ-E)(*m*BDCA-5t)]^2–^ (E = Se, S) polychalcogenide complexes is likely due to the favorable geometry as a result of the formation of the five membered chalcogenide ring, as the solid-state structure of **3** shows the Se_5_ ring is similar in size to one of the aromatic spacer arms of the cryptand. A similar phenomenon has been observed for polychalcogenide tin Tb complexes (Tb = 2,4,6-tris[bis(trimethylsilyl)methyl]phenyl) reported by Okazaki.^33^

**Fig. 6:**
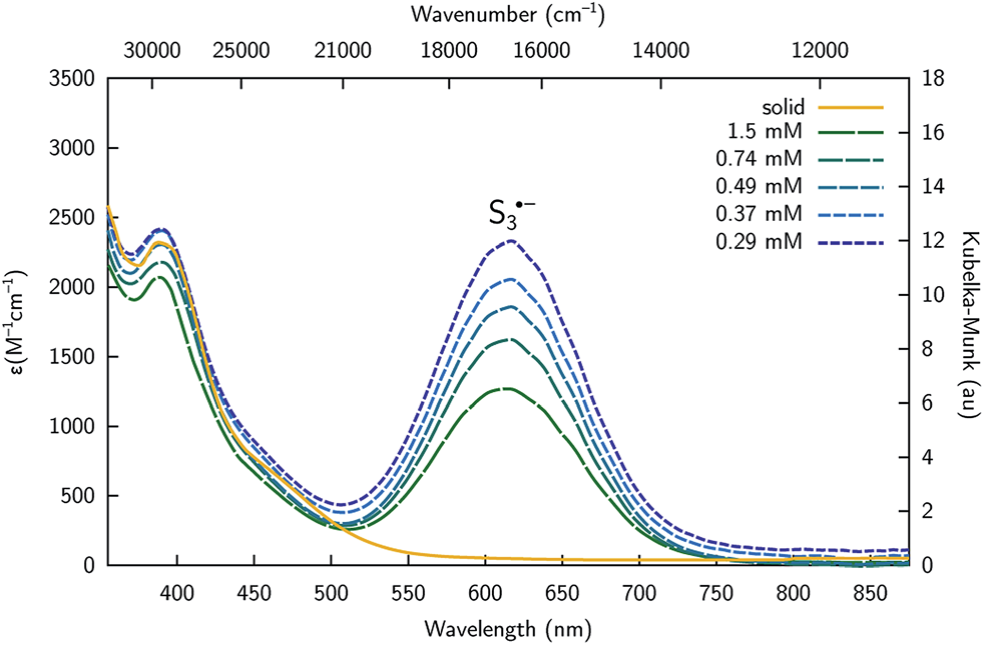
UV-Vis spectra of [K_2_(DMF)_3_][**4**] in the solid state (yellow), and in DMF at 20 °C collected at varying concentration showing the presence of S_3_˙^–^ (*λ*_max_ = 617 nm) in solution.

Complex **4** is cleanly isolated as a yellow solid; however, when the yellow solid is dissolved in DMF or DMSO, the color of the resulting solution is dark blue-green. In order to investigate the cause of this color change, both solution and solid-state UV-Vis spectra of **4** were obtained. The solution UV-Vis spectrum of **4** in DMF shows two major absorptions at *λ*_max_ = 392 and 617 nm, while the diffuse reflectance UV-Vis spectrum shows only one major absorption at *λ*_max_ = 392 nm (Fig. 6). The absorption at *λ*_max_ = 617 nm that is only present in the solution spectrum is consistent with the literature reported absorption band of trisulfur radical anion (S_3_˙^–^).^34^ These data suggest that S_3_˙^–^ dissociates from the complex in solution possibly as indicated by eqn (1), and is therefore responsible for the marked difference in color between solution and the solid state for this system. The trisulfur radical anion has been extensively studied and is known as the blue color center in ultramarine pigments in which this highly reactive species is trapped within a zeolite framework; S_3_˙^–^ is also responsible for the blue color in the naturally occurring mineral, lapis lazuli.^35^

1

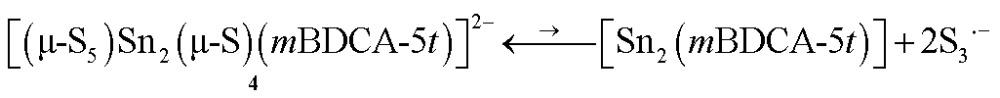




It is known that S_3_˙^–^ is readily formed from its S_6_^2–^ dimer *via* dissociation in an entropy-driven process that is favored by high temperatures and in dilute solutions in highly polar solvents such as DMF, HMPA, and DMSO.^36^ These properties are consistent with observations made using the present system, as variable temperature UV-Vis spectra of complex **4** display a significant increase in the amount of S_3_˙^–^ as the temperature of the solution increases from 15 to 85 °C (Fig. S24†). Similarly, UV-Vis studies of complex **4** indicate that the amount of S_3_˙^–^ in solution increases as the concentration of **4** decreases as indicated by its molar extinction value that maximizes at *ε* = 2330 M^–1^ cm^–1^ (0.29 mM, Fig. 6). The equilibrium constant (*K*_eq_) for S_3_˙^–^ dissociation from complex **1** based upon eqn (1) was calculated to be 0.012 ± 0.002 in DMF solution at 20 °C, indicating the equilibrium lies heavily to the left. The presence of S_3_˙^–^ was also confirmed by electron paramagnetic resonance (EPR) spectroscopy, where a broad signal centred at *g* = 2.0290 was observed in DMF solution at 20 °C (Fig. S25†). This signal is in agreement with the literature reported EPR signal of S_3_˙^–^.^37^ Therefore, the observed data suggest that in solution, the sulfur atoms of complex **4** undergo dissociation from the di-tin cryptand to release S_3_˙^–^.

The presence of S_3_˙^–^ upon dissolution of **4** may be a result of initial release of S_6_^2–^ followed by dissociation into two equivalents of S_3_˙^–^. According to this overall stoichiometry, dissociation of two equivalents of S_3_˙^–^ from the dianionic complex **4** would require the concomitant formation of a neutral [Sn_2_(*m*BDCA-5t)] species. Although this is perhaps the most straightforward explanation, we cannot definitively rule out S_5_^2–^ or S_4_^2–^ as the source of S_3_˙^–^. While more work is required to substantiate this proposal, such a postulated neutral [Sn_2_(*m*BDCA-5t)] species may exist as a Sn–Sn bonded hexaaminodistannane analogous to one which Gade *et al.* obtained upon oxidative coupling of a tin(ii) triamidostannate and for which the Sn–Sn interatomic distance was reported to be 2.8204(4) Å.^38^ The metal–metal single bond in such a species corresponds to the HOMO–1 of complex **1** (Fig. 2b).

In an effort to drive to full completion the level of sulfur/selenium reduction in this system, we probed the reaction of **3** and **4** with PR_3_ reductants (R = ^*t*^Bu, Ph, O^i^Pr, Scheme 2). Proceeding accordingly, we found that treatment of either complex with PR_3_ (6 equiv.) resulted in abstraction of all six chalcogen atoms and quantitative regeneration of di-tin(ii) complex **1**! Incomplete conversion of **3** and **4** to **1** was the result when using <6 equiv. PR_3_, with no other species observed by ^1^H or ^119^Sn NMR spectroscopy. Treatment of either **3** or **4** with PMes_3_ (excess), however, led to no observed reaction, this being explicable in terms of steric effects. Dechalcogenation of transition^39^ and group 14 (ref. 31 and 40) metal cyclic polychalcogenide complexes is commonly accomplished using tertiary phosphines or electron deficient alkenes and alkynes. These reactions, however, typically result in only partial reduction in the size of the chalcogen ring or conversion to the typical bis-μ-chalcogen thermodynamic product instead of complete dechalcogenation. The present polychalcogen/di-tin cryptand complexes are very unusual inasmuch as PR_3_ compounds are capable of complete de-chalcogenation and reduction of tin back to the +2 oxidation state, suggesting in turn that the macrobicyclic nature of bimetallic complex **1** imbues the tin(ii) oxidation state with comparatively greater stability than is typical sans such structural constraints.

## References

[cit1] Babcock G. T., Wikstrom M. (1992). Nature.

[cit2] Gueutin C., Lexa D., Savéant J.-M., Momenteau M. (1988). J. Electroanal. Chem. Interfacial Electrochem..

[cit3] Proni G., Pescitelli G., Huang X., Quraishi N. Q., Nakanishi K., Berova N. (2002). Chem. Commun..

[cit4] Shimazaki Y., Nagano T., Takesue H., Ye B.-H., Tani F., Naruta Y. (2004). Angew. Chem., Int. Ed..

[cit5] (b) Basset J. M. and Ugo R., in On the Origins and Development of “Surface Organometallic Chemistry”, Wiley-VCH Verlag GmbH & Co. KGaA, 2009, pp. 1–21.

[cit6] Collman J. P., Wagenknecht P. S., Hutchison J. E. (1994). Angew. Chem., Int. Ed. Engl..

[cit7] Chang C. K., Liu H. Y., Abdalmuhdi I. (1984). J. Am. Chem. Soc..

[cit8] Collman J. P., Hutchison J. E., Lopez M. A., Guilard R., Reed R. A. (1991). J. Am. Chem. Soc..

[cit9] Naruta Y., Maruyama K. (1991). J. Am. Chem. Soc..

[cit10] Alliger G. E., Mueller P., Cummins C. C., Nocera D. G. (2010). Inorg. Chem..

[cit11] Alliger G. E., Mueller P., Do L. H., Cummins C. C., Nocera D. G. (2011). Inorg. Chem..

[cit12] Spikes G. H., Fettinger J. C., Power P. P. (2005). J. Am. Chem. Soc..

[cit13] Power P. P. (2010). Nature.

[cit14] Bruce P. G., Freunberger S. A., Hardwick L. J., Tarascon J.-M. (2011). Nat. Mater..

[cit15] Explanation of *m*BDCA-5t-H_6_ shorthand: *m*BDCA denotes a *meta*-substituted derivative of benzene dicarboxylic acid for the three spacers joining two tren, tris-2-aminoethylamine, end caps. 5*t* refers to a *tert*-butyl group at the 5-position of each substituted benzene spacer, and H_ *n* _ is the state of protonation of the six carboxamide groups (*n* = 0–6)

[cit16] Gynane M. J. S., Harris D. H., Lappert M. F., Power P. P., Riviere P., Riviere-Baudet M. (1977). J. Chem. Soc., Dalton Trans..

[cit17] Spek A. L. (2009). Acta Crystallogr., Sect. D: Biol. Crystallogr..

[cit18] (b) Singh A. K. and Sonali B., Main Group Metal Chemistry, 2011, vol. 26, pp. 155–211.

[cit19] Neese F. (2012). Wiley Interdiscip. Rev.: Comput. Mol. Sci..

[cit20] Grimme S. (2006). J. Chem. Phys..

[cit21] Grimme S., Ehrlich S., Goerigk L. (2011). J. Comput. Chem..

[cit22] van Lenthe E., Ehlers A., Baerends E.-J. (1999). J. Chem. Phys..

[cit23] Tsai M.-L., Chen C.-C., Hsu I.-J., Ke S.-C., Hsieh C.-H., Chiang K.-A., Lee G.-H., Wang Y., Chen J.-M., Lee J.-F., Liaw W.-F. (2004). Inorg. Chem..

[cit24] Tokitoh N., Matsumoto T., Okazaki R. (1991). Tetrahedron Lett..

[cit25] Matsumoto T., Matsui Y., Ito M., Tatsumi K. (2008). Chem.–Asian J..

[cit26] Zhou Y., Richeson D. S. (1996). J. Am. Chem. Soc..

[cit27] Schranz I., Grocholl L., Carrow C. J., Stahl L., Staples R. J. (2008). J. Organomet. Chem..

[cit28] Eisler D. J., Chivers T. (2006). Chem.–Eur. J..

[cit29] Ahrika A., Robert J., Anouti M., Paris J. (2001). New J. Chem..

[cit30] Chivers T., Eisler D. J. (2004). Angew. Chem., Int. Ed..

[cit31] Foley S. R., Yap G. P. A., Richeson D. S. (1999). Organometallics.

[cit32] Donahue J. P. (2006). Chem. Rev..

[cit33] Matsuhashi Y., Tokitoh N., Okazaki R., Goto M., Nagase S. (1993). Organometallics.

[cit34] Chivers T., Drummond I. (1972). Inorg. Chem..

[cit35] Clark R. J. H., Cobbold D. G. (1978). Inorg. Chem..

[cit36] Chivers T. (1974). Nature.

[cit37] Pinon V., Levillain E., Lelieur J. P. (1991). J. Phys. Chem..

[cit38] Lutz M., Haukka M., Pakkanen T. A., Gade L. H. (2003). Z. Anorg. Allg. Chem..

[cit39] Bolinger C. M., Rauchfuss T. B., Wilson S. R. (1981). J. Am. Chem. Soc..

[cit40] Saito M., Tokitoh N., Okazaki R. (1997). J. Am. Chem. Soc..

