# Supplementary material for: Multi-electron reactivity of a cofacial di-tin(ii) cryptand: partial reduction of sulfur and selenium and reversible generation of S3˙–
Source: Chem Sci. 2016 Jul 6;7(12):6928–33. doi: 10.1039/c6sc01754a (PMC5450590; doi:10.1039/c6sc01754a)
Supplement: Supplementary file 1 [file SC-007-C6SC01754A-s001.pdf]

Electronic Supplementary Material (ESI) for Chemical Science.
This journal is © The Royal Society of Chemistry 2016

Supporting Information for the paper entitled, Multi-electron reactivity of a cofacial di-tin(II) cryptand: Partial reduction of sulfur and selenium and reversible generation of $S_3^{\bullet-}$

Julia M. Stauber,[†] Peter Müller,[†] Yizhe Dai,[‡] Gang Wu,[*,‡] Daniel G. Nocera,[*,¶] and Christopher C. Cummins[*,†]

Department of Chemistry, Massachusetts Institute of Technology, 77 Massachusetts Avenue, Cambridge, MA 02139-4307, Department of Chemistry, Queen's University, 90 Bader Lane, Kingston, Ontario, Canada K7L 3N6, and Department of Chemistry and Chemical Biology, Harvard University, 12 Oxford Street, Cambridge, MA 02138-2902

E-mail: gang.wu@chem.queensu.ca; dnocera@fas.harvard.edu; ccummins@mit.edu

Contents

1 General methods **S4**

2 Experimental details and characterization of Sn and Pb complexes **S6**

2.1 Synthesis of $[K_2(THF)][Sn_2(m\text{BDCA-5t})]$ ($[K_2(THF)][\mathbf{1}]$) S6

*To whom correspondence should be addressed

†Department of Chemistry, Massachusetts Institute of Technology, 77 Massachusetts Avenue, Cambridge, MA 02139-4307

‡Department of Chemistry, Queen's University, 90 Bader Lane, Kingston, Ontario, Canada K7L 3N6

¶Department of Chemistry and Chemical Biology, Harvard University, 12 Oxford Street, Cambridge, MA 02138-2902

2.2 Synthesis of [K(18-crown-6)]$_2$[Sn$_2$(mBDCA-5t)] ([K(18-crown-6)]$_2$[**1**]). S11
2.3 Synthesis of [K(Kryptofix-2,2,2)]$_2$[Sn$_2$(mBDCA-5t)] ([K(Kryptofix-2,2,2)]$_2$[**1**]) . . S12
2.4 Synthesis of [K$_2$(THF)][Pb$_2$(mBDCA-5t)] ([K$_2$(THF)][**2**]) S13
2.5 Synthesis of [K(Kryptofix-2,2,2)]$_2$[Pb$_2$(mBDCA-5t)] ([K(Kryptofix-2,2,2)]$_2$[**2**]) . . S18
2.6 Synthesis of [K$_2$(DMF)$_3$][(μ–Se$_5$)Sn$_2$(μ–Se)(mBDCA-5t)] ([K$_2$(DMF)$_3$][**3**]) . . . S20
2.7 Synthesis of [K$_2$(DMF)$_3$][(μ–S$_5$)Sn$_2$(μ–S)(mBDCA-5t)] ([K$_2$(DMF)$_3$][**4**]) S26

3 Selenium atom transfer (SeAT) and sulfur atom transfer (SAT) from 3 and 4 to PR$_3$ (R = Ph, ^{t}Bu, O^{i}Pr) S34

3.1 SeAT S34
3.1.1 Treatment of **3** with PPh$_3$ in DMSO-d_6 . . . . S34
3.1.2 Monitoring the reaction between **3** and PPh$_3$ by UV-Vis. S37
3.1.3 Treatment of **3** with P(^{t}Bu)$_3$ in DMSO-d_6 . . . . S38
3.1.4 Treatment of **3** with P(O^{i}Pr)$_3$ in DMSO-d_6 . . . . S40
3.2 SAT S42
3.2.1 Treatment of **4** with PPh$_3$ in DMSO-d_6 . . . . S42
3.2.2 Monitoring the reaction between **4** and PPh$_3$ by UV-Vis S45
3.2.3 Treatment of **4** with P(^{t}Bu)$_3$ in DMSO-d_6 . . . . S46
3.2.4 Treatment of **4** with P(O^{i}Pr)$_3$ in DMSO-d_6 . . . . S48

4 Electrochemistry of [K(18-crown-6)]$_2$[1] S50

5 Calculation of the [(μ-S$_5$)Sn$_2$(μ-S)(mBDCA-5t)]$^{2-}$ $\rightleftharpoons$ 2S$_3^{\bullet-}$ + [Sn$_2$(mBDCA-5t)] equilibrium constant S51

6 Computational details for ^{77}Se and ^{119}Sn NMR calculations of complex 3 and simulated spectra S52

6.1 Computed NMR parameters S53
6.1.1 X, Y, Z coordinates of truncated model complex (Figure S45) S54

6.2 Experimental and simulated spectra S57

7 Computational details for model complex, $[Sn_2(m\text{BDCA-5H})]^{2-}$ S61

7.1 X, Y, Z coordinates of model complex, $[Sn_2(m\text{BDCA-5H})]^{2-}$ S61

8 Crystallographic information for $[K(\text{Kryptofix-2,2,2})]_2$[1], $[K(\text{Kryptofix-2,2,2})]_2$[2], and $[K_2(\text{DMF})_3]$[3] S64

8.1 X-ray crystal structure determination details S64

8.2 Solid-state structure of $[K(\text{Kryptofix-2,2,2})]_2$[**2**] S66

1 General methods

All manipulations were performed either using Schlenk techniques or in a Vacuum Atmospheres model MO-40M glovebox under an inert atmosphere of purified N_2. All reagents were purchased from Sigma Aldrich, Strem Chemicals or Alfa Aesar. 18-crown-6 was purified by crystallization from dry acetonitrile at –35 °C, and S_8 was crystallized from refluxing toluene. All other chemicals were used as received. The *m*BDCA-5t-H_6 cryptand was prepared as previously reported,[1] and both $Sn[N(SiMe_3)_2]_2$ and $Pb[N(SiMe_3)_2]_2$ were prepared according to literature procedures.[2] Solvents were either purified on a Glass Contour Solvent Purification System built by SG Water USA, LLC, or prepared according to literature procedures. DMSO-d_6 was obtained from Cambridge Isotope Laboratories and was degassed and stored over molecular sieves (4 Å beads, 8-12 mesh) for at least 2 days prior to use. All NMR spectra were obtained on Bruker Avance 400 instruments equipped with Magnex Scientific superconducting magnets or a VARIAN Inova-500 instrument equipped with an Oxford Instruments Ltd. superconducting magnet, and ^{1}H NMR and ^{13}C{^{1}H} NMR spectra are referenced to residual protio-solvent signals. ^{119}Sn NMR spectra were referenced to Me_4Sn (90% in C_6D_6, δ = 0.0 ppm), ^{77}Se NMR spectra were referenced to Ph_2Se_2 (C_6D_6, δ = 463 ppm), and ^{207}Pb NMR spectra were referenced to a 1 M solution of $Pb(NO_3)_2$ in ACS reagent grade H_2O (δ –2961 ppm).[3] ^{77}Se and ^{119}Sn NMR spectra were simulated using the program gNMR version 5.1. Celite 435 (EM Science) was dried by heating above 200 °C under dynamic vacuum for at least 48 h prior to use. ESI-MS data were obtained on a Waters Q-TOF micro mass spectrometer using a source temperature of 100 °C and a desolvation temperature of 150 °C. ESI-MS samples were run in neat DMF at concentrations < 1 μM and the data were processed using the program mMass Version 5.4.1.0. Elemental analyses were performed by Complete Analysis Laboratories Inc (CALI, http://www.calilabs.com). UV-Vis spectra were collected with an Ocean Optics USB4000 spectrophotometer and a DT-Mini-2GS UV-vis-NIR light source equipped with a qpod 2e temperature controller. Diffuse reflectance UV-Vis spectra were collected between 200–2000 nm on a Varian Cary 5000 UV-Vis-NIR spectrometer equipped with a Praying

Mantis diffuse reflectance accessory and an environmental chamber (Harrick Scientific Products) and referenced to $BaSO_4$. X-band EPR samples of **3** and **4** were run in dry DMF (11 mM) at 25 °C using a Bruker EMX spectrometer equipped with an ER 4199HS cavity and a Gunn diode microwave source. Complex **3** displayed a silent EPR spectrum when measured at 25 °C in DMF solution.

ICP-AES measurements: Lead, tin, and potassium ICP-AES analyses were conducted at the MIT Center for Materials Science and Engineering Shared Experimental Facility (CMSE-SEF) using a HORIBA Jobin ACTIVA inductively coupled plasma atomic emission spectrometer (ICP-AES). Solutions of standard concentrations were used for calibration purposes and were prepared from solutions purchased from ULTRA Scientific or Sigma Aldrich, designated suitable for ICP analysis. Standard solutions for potassium were prepared with concentrations of 10, 20, 30, 40, and 50 ppm. Tin standard solutions were prepared with concentrations of 50, 70, 90, 110, and 130 ppm, and lead standard solutions were prepared with concentrations of 20, 30, 50, 60, and 70 ppm. The samples of [K(18-cown-6)]$_2$[**1**], [K(Kryptofix 2,2,2)]$_2$[**1**], and [K(Kryptofix 2,2,2)]$_2$[**2**] consisted of 2% HNO_3 solutions containing the bimetallic cryptand at concentrations in the range of 20-30 ppm K, 60 ppm Pb, and 60 ppm Sn. To prepare these solutions, 300-500 μL of 70% Omni-Trace grade HNO_3 was added to [K(18-crown-6)]$_2$[**1**], [K(Kryptofix 2,2,2)]$_2$[**2**] and [K(Kryptofix 2,2,2)]$_2$[**2**] powders. The samples were then sonicated to ensure complete dissolution of the solids, diluted with Milli-Q water to obtain 2% HNO_3 solutions, and then filtered through a 0.2 micron filter. Potassium analyses were run with λ = 766.491 nm; tin analyses were run with λ = 283.998, 242.950, and 189.925 nm, and lead analyses were run with λ = 220.353, 217.000, and 182.143 nm. The K/Sn ratios are reported as the averages of the data acquired for each sample for the three wavelengths chosen for tin and the K/Pb ratio is reported as the average for three wavelengths chosen for lead.

2 Experimental details and characterization of Sn and Pb complexes

2.1 Synthesis of [K_2(THF)][Sn_2(*m*BDCA-5t)] ([K_2(THF)][1])

In the glovebox, a solution of KO^{t}Bu (65 mg, 0.58 mmol, 2.2 equiv) and Sn[N($SiMe_3$)$_2$]$_2$ (255 mg, 0.579 mmol, 2.20 equiv) in THF (2 mL) was added to a stirring suspension of *m*BDCA-5t-H_6 (224 mg, 0.263 mmol, 1.00 equiv) in THF (1 mL). The colorless suspension was allowed to stir at glovebox temperature (23 °C) for a total of 10 h, at which point the colorless solids were isolated via filtration on a medium-porosity fritted funnel, washed with THF (10 mL), and dried under reduced pressure affording [K_2(THF)][**1**] as a colorless powder (yield: 220 mg, 0.178 mmol, 68%). ^{1}H NMR (DMSO-d_6, 25 °C, 400.1 MHz, δ): 6.91 (d, 6H), 6.27 (t, 3H), 3.86 (d, 6H), 2.81 (m, 6H), 2.61 (m, 12H), 1.21 (s, 27H) ppm. ^{13}C{^{1}H} NMR (DMSO-d_6, 25 °C, 100.6 MHz, δ): 174.9, 149.2, 141.9, 123.3, 122.9, 67.0 (THF), 56.4, 41.1, 34.1, 31.2, 25.1 (THF) ppm. ^{119}Sn{^{1}H} NMR (DMSO-d_6, 25 °C, 149.2 MHz, δ): –381.6 ppm. ESI-MS(−): 541.12 m/z (calc'd, 541.14 m/z). Satisfactory elemental analysis results were not obtained for this complex due to its variable solvent content. Removal of coordinated THF molecules by sequestration of the K^+ cations with 18-crown-6 did yield satisfactory elemental analysis results, and the synthetic details and characterization of [K(18-crown-6)]$_2$[**1**] are described in the following section.

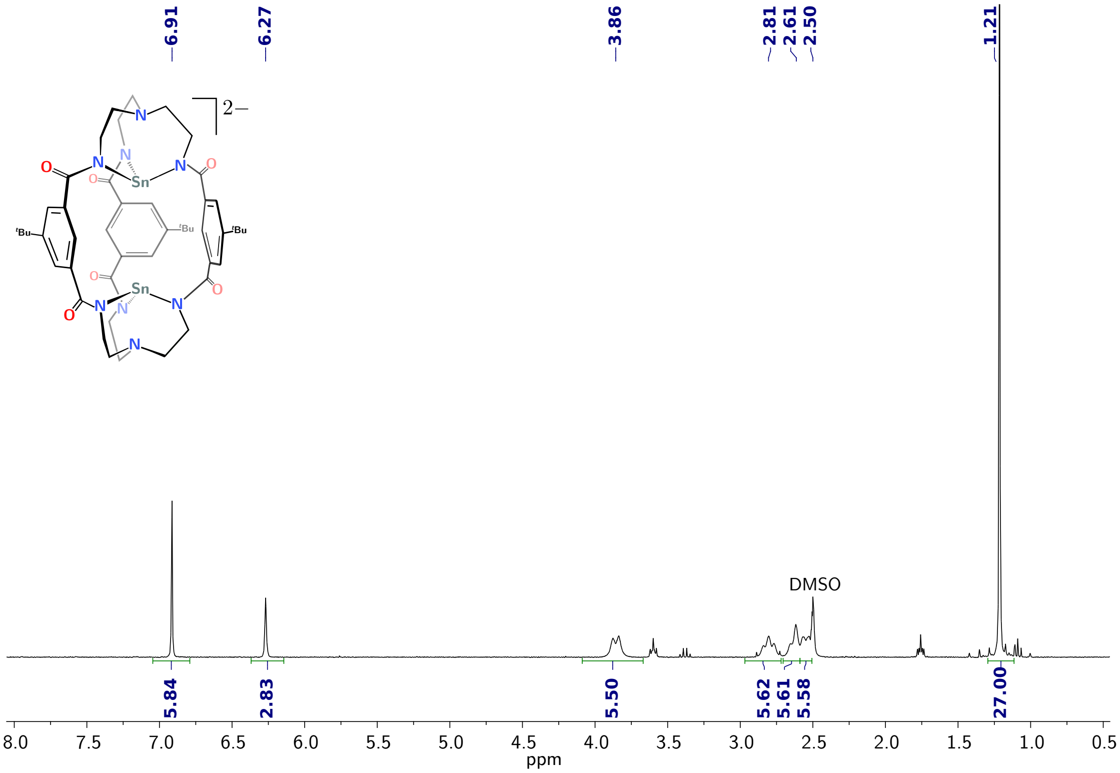

Figure S1: ^{1}H NMR spectrum of [K_2(THF)][**1**] in DMSO-d_6 (400.1 MHz, 25 °C).

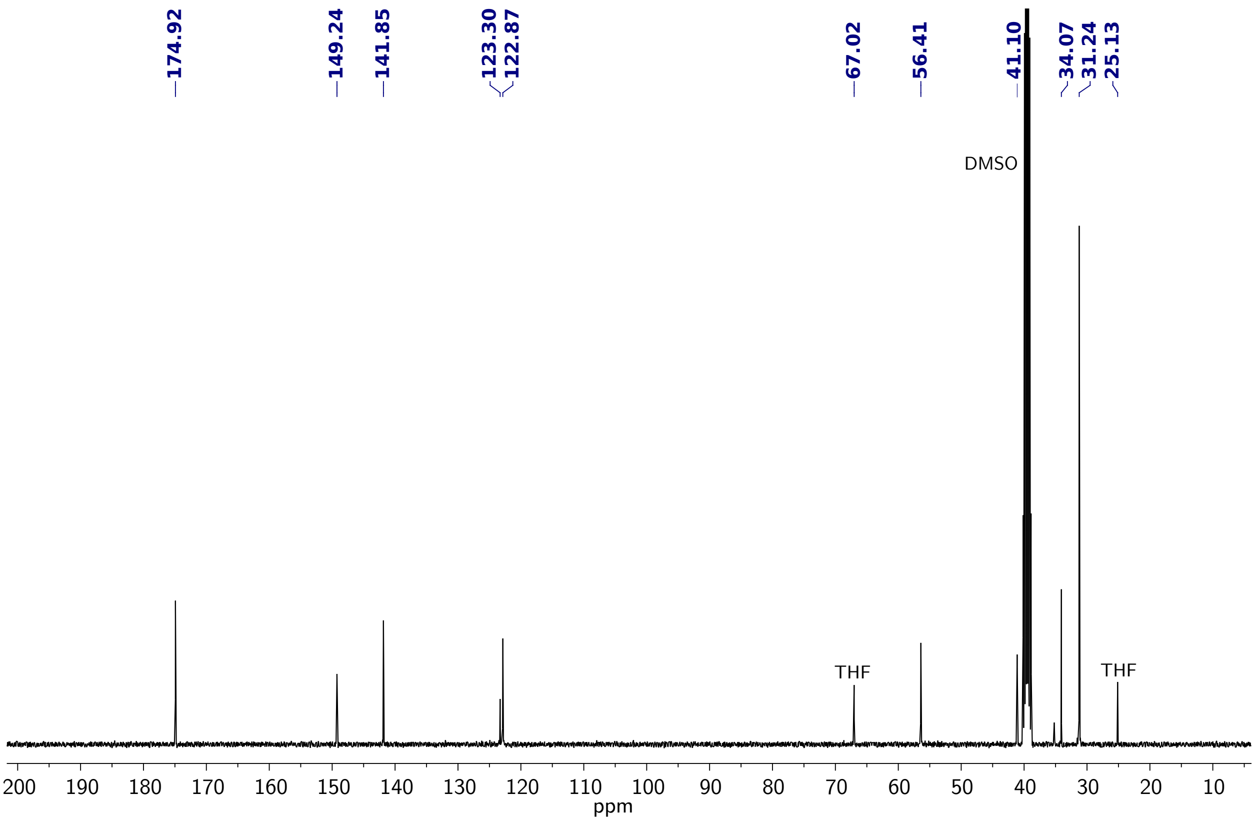

Figure S2: $^{13}C\{^{1}H\}$ NMR spectrum of $[K_2(THF)][\mathbf{1}]$ in DMSO-d_6 (100.6 MHz, 25 °C).

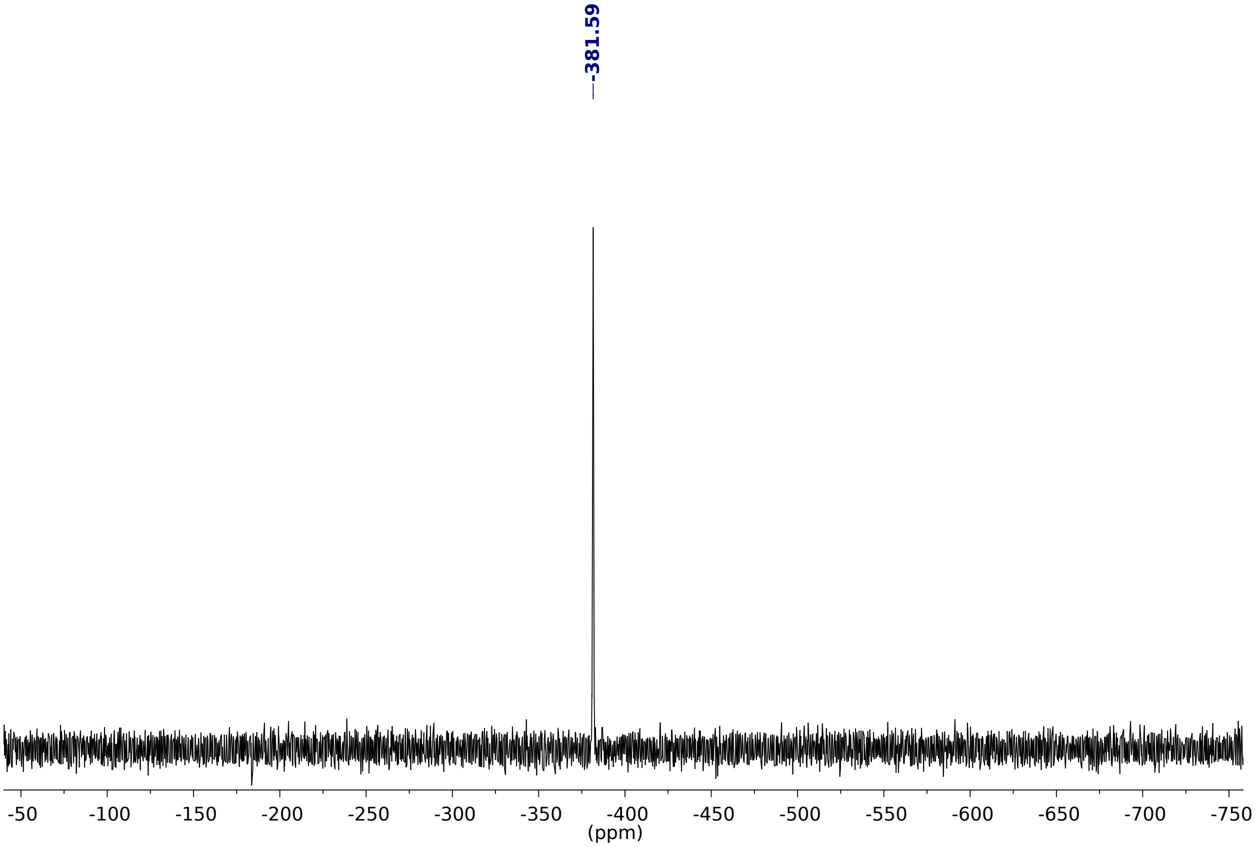

Figure S3: $^{119}Sn\{^1H\}$ NMR spectrum of $[K_2(THF)][\mathbf{1}]$ in DMSO-d_6 (149.2 MHz, 25 °C).

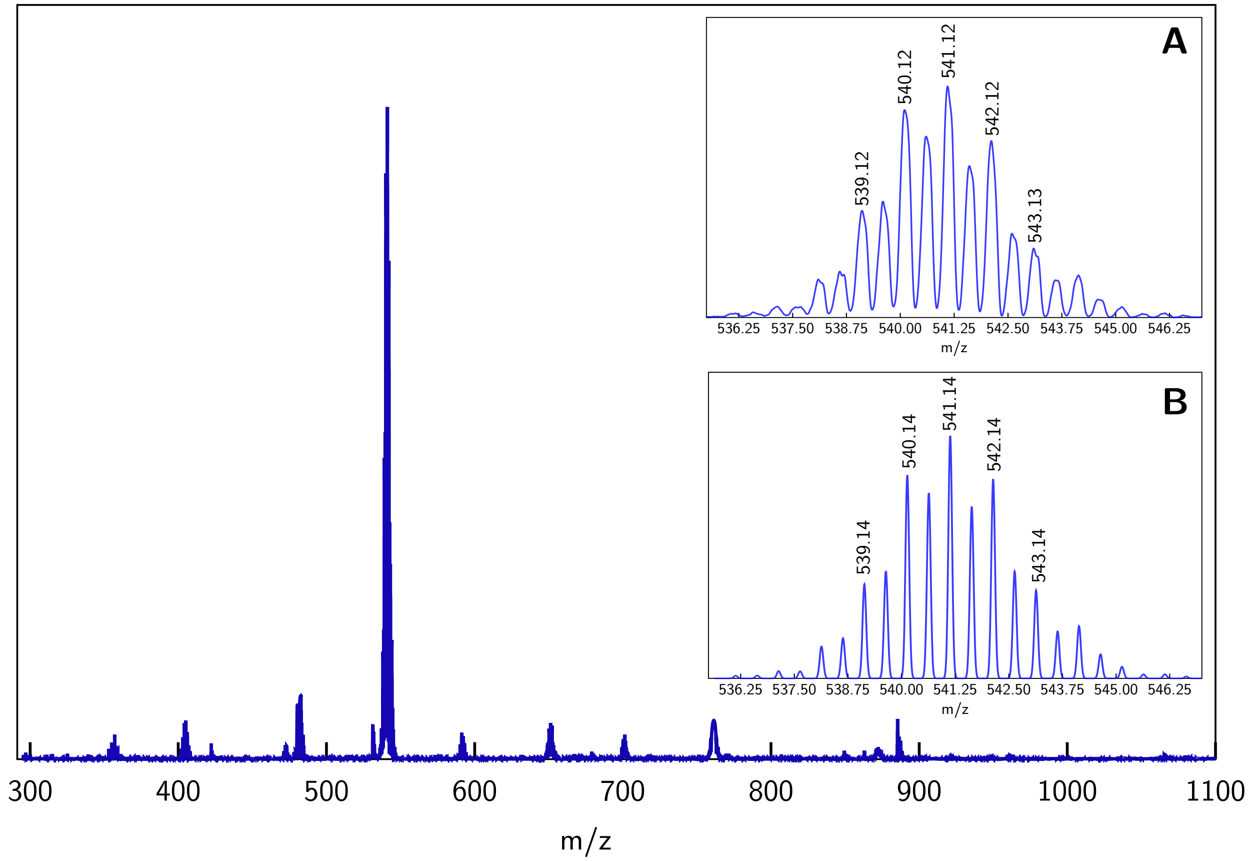

Figure S4: ESI-MS(–) of [K_2(THF)][**1**]. A: zoomed-in spectrum, B: simulated spectrum (DMF, 3200 V).

2.2 Synthesis of $[K(18\text{-crown-}6)]_2[Sn_2(m\mathrm{BDCA}\text{-}5t)]$ ($[K(18\text{-crown-}6)]_2[\mathbf{1}]$).

Solid 18-crown-6 (43 mg, 0.16 mmol, 2.0 equiv) was added to a stirring suspension of $[K_2(THF)][\mathbf{1}]$ (100 mg, 0.0811 mmol, 1.00 equiv) in THF (3 mL) at glovebox temperature (23 °C). The colorless suspension was allowed to stir for a total of 1 h, at which point the solids were isolated by filtration, washed with THF (3 mL), and dried under reduced pressure to afford $[K(18\text{-crown-}6)]_2[\mathbf{1}]$ as a colorless powder (123 mg, 0.0729 mmol, 90%). ^{1}H NMR (DMSO-d_6, 25 °C, 400.1 MHz, δ): 6.91 (d, 6H), 6.27 (t, 3H), 3.84 (d, 6H), 3.54 (s, 48H, 18-crown-6), 2.80 (m, 6H), 2.62 (m, 12H), 1.22 (s, 27H) ppm. Anal. Calc'd (found) for $C_{72}H_{108}N_8O_{18}K_2Sn_2$: C, 51.20 (51.56); H, 6.44 (6.22); N, 6.63 (6.31). ICP-AES results show a K/Sn ratio of 1.06 ± 0.01.

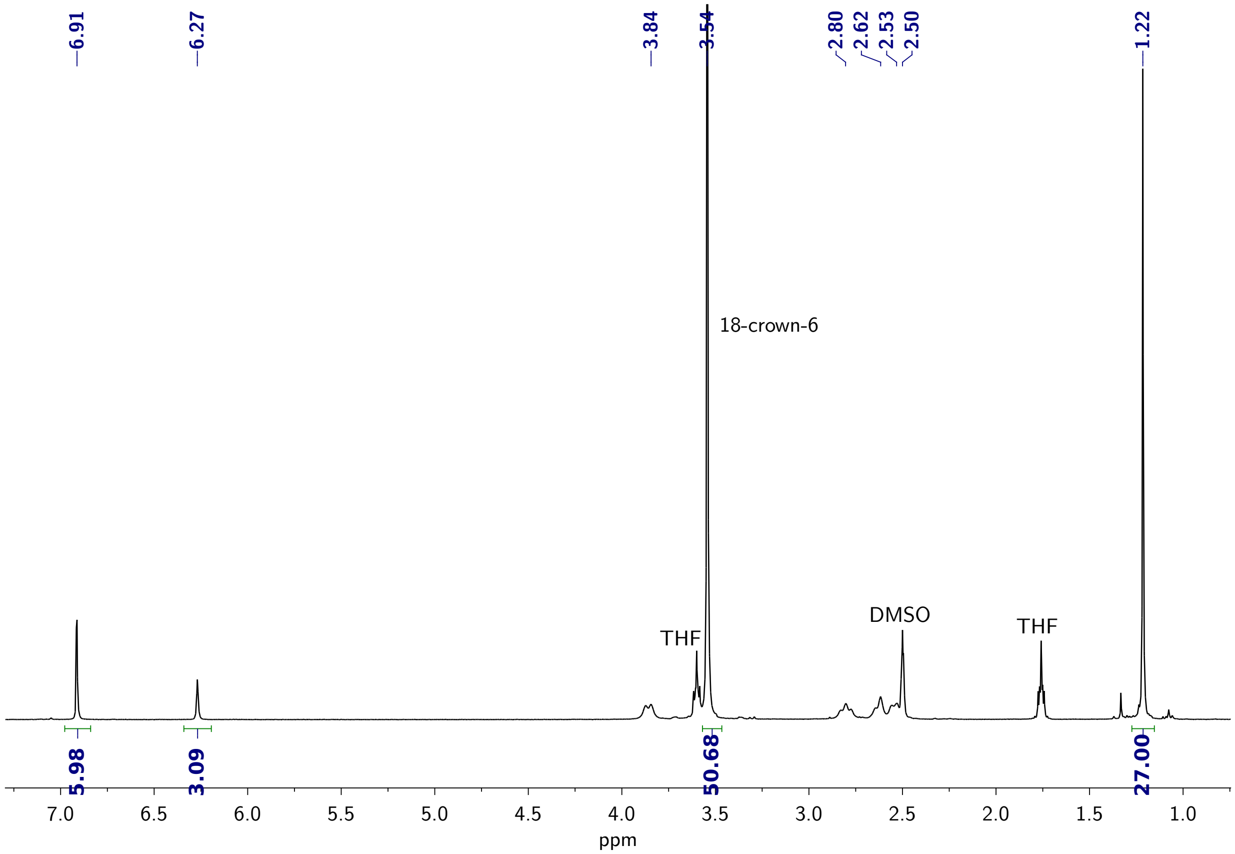

Figure S5: ^{1}H NMR spectrum of $[K(18\text{-crown-}6)]_2[\mathbf{1}]$ in DMSO-d_6 (400.1 MHz, 25 °C).

2.3 Synthesis of [K(Kryptofix-2,2,2)]$_2$[Sn$_2$(*m*BDCA-5t)] ([K(Kryptofix-2,2,2)]$_2$[**1**])

In the glovebox, THF (2 mL) was added to solid [K$_2$(THF)][**1**] (100 mg, 0.081 mmol, 1.0 eq) and Kryptofix-2,2,2 (61 mg, 0.16 mmol, 2.0 eq). The colorless suspension was allowed to stir at glovebox temperature (23 °C) for a total of 1 h, at which point the solids were isolated by filtration, washed with THF (1 mL), and dried under reduced pressure affording [K(Kryptofix-2,2,2)]$_2$[**1**] as a colorless powder (yield: 112 mg, 0.058 mmol, 72%). Colorless X-ray quality crystals were grown by vapor diffusion of Et$_2$O into a saturated DMF solution of [K(Kryptofix-2,2,2)]$_2$[**1**] over the course of 24 h at 23 °C. ^{1}H NMR (DMSO-d_6, 25 °C, 400.1 MHz, δ): 6.92 (d, 6H), 6.27 (t, 3H), 3.87 (d, 6H), 3.55 (s, 24H, Kryptofix-2,2,2), 3.51 (m, 24H, Kryptofix-2,2,2), 2.80 (m, 6H), 2.62 (m, 12H), 2.50 (m, 24H, Kryptofix-2,2,2 overlapping with DMSO solvent), 1.22 (s, 27H) ppm. ICP-AES results show a K/Sn ratio of 1.07 ± 0.01.

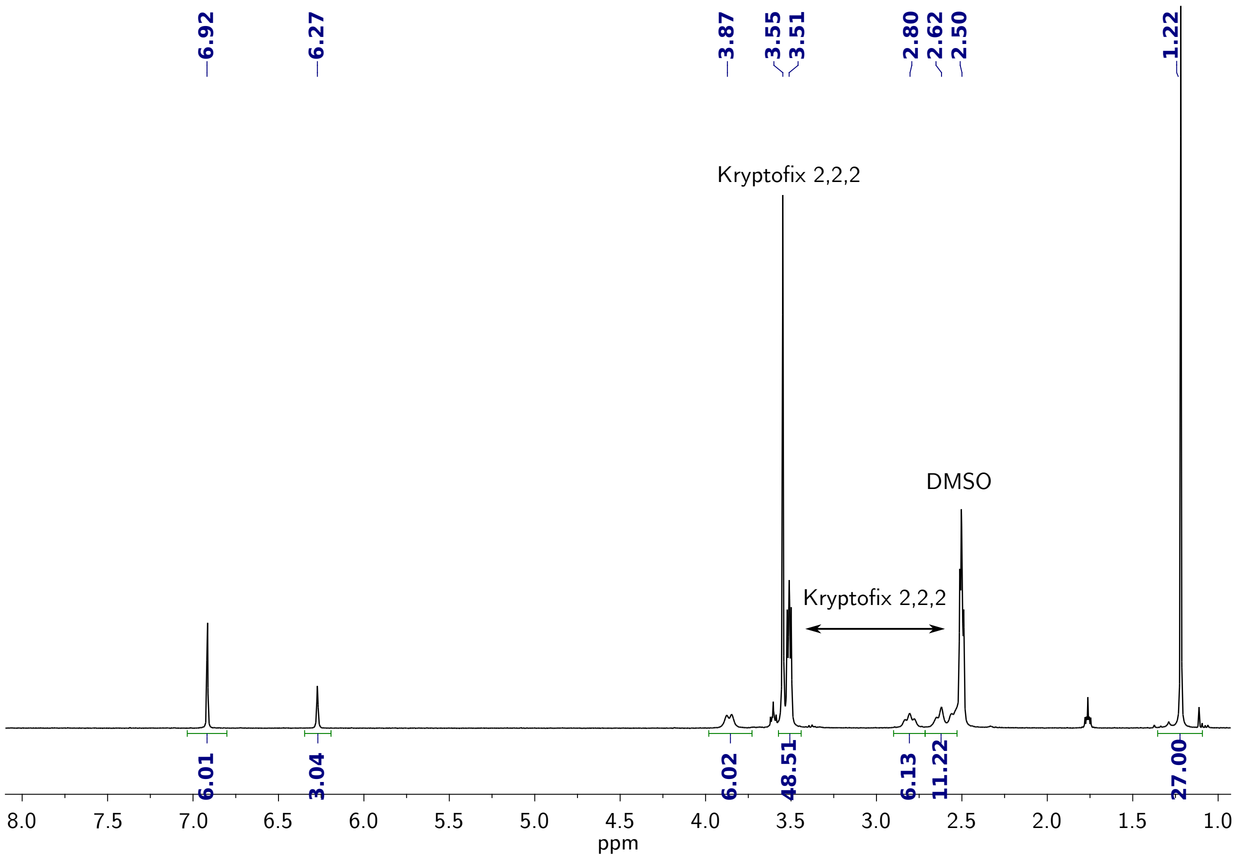

Figure S6: ^{1}H NMR spectrum of [K(Kryptofix-2,2,2)]$_2$[**1**] in DMSO-d_6 (400.1 MHz, 25 °C).

2.4 Synthesis of $[K_2(THF)][Pb_2(m\mathbf{BDCA\text{-}5t})]$ ($[K_2(THF)][\mathbf{2}]$)

In the glovebox, a solution of KO^tBu (73 mg, 0.65 mmol, 2.2 equiv) and $Pb[N(SiMe_3)_2]_2$ (342 mg, 0.647 mmol, 2.20 equiv) in THF (2 mL) was added to a stirring suspension of *m*BDCA-5t-H_6 (250 mg, 0.294 mmol, 1.00 equiv) in THF (1 mL). The colorless suspension was allowed to stir at glovebox temperature (23 °C) for a total of 10 h, at which point the colorless solids were isolated via filtration on a medium-porosity fritted funnel, washed with THF (10 mL), and dried under reduced pressure affording $[K_2(THF)]$[**2**] as a colorless powder (yield: 300 mg, 0.202 mmol, 69%). ^1H NMR (DMSO-d_6, 25 °C, 400.1 MHz, δ): 7.09 (d, 6H), 6.46 (t, 3H), 4.48 (d, 6H), 2.84 (m, 6H), 2.64 (m, 6H), 2.40 (m, 6H), 1.25 (s, 27H) ppm. $^{13}C\{^1H\}$ NMR (DMSO-d_6, 25 °C, 100.6 MHz, δ): 174.8, 150.1, 142.2, 123.2, 121.9, 67.0 (THF), 57.4, 43.1, 34.1, 31.2, 25.1 (THF) ppm. ^{207}Pb NMR (DMSO-d_6, 25 °C, 105 MHz, δ): –2641.4 ppm ($\Delta\nu_{1/2}$ = 1160 Hz). ESI-MS(−): 630.18 m/z (calc'd, 630.21 m/z). Satisfactory elemental analysis results were not obtained for this complex due to its variable solvent content. Removal of coordinated THF molecules by sequestration of the K^+ cations with Kryptofix-2,2,2 did yield satisfactory elemental analysis results, and the synthetic details and characterization of $[K(Kryptofix\text{-}2,2,2)]_2$[**2**] are described in the following section.

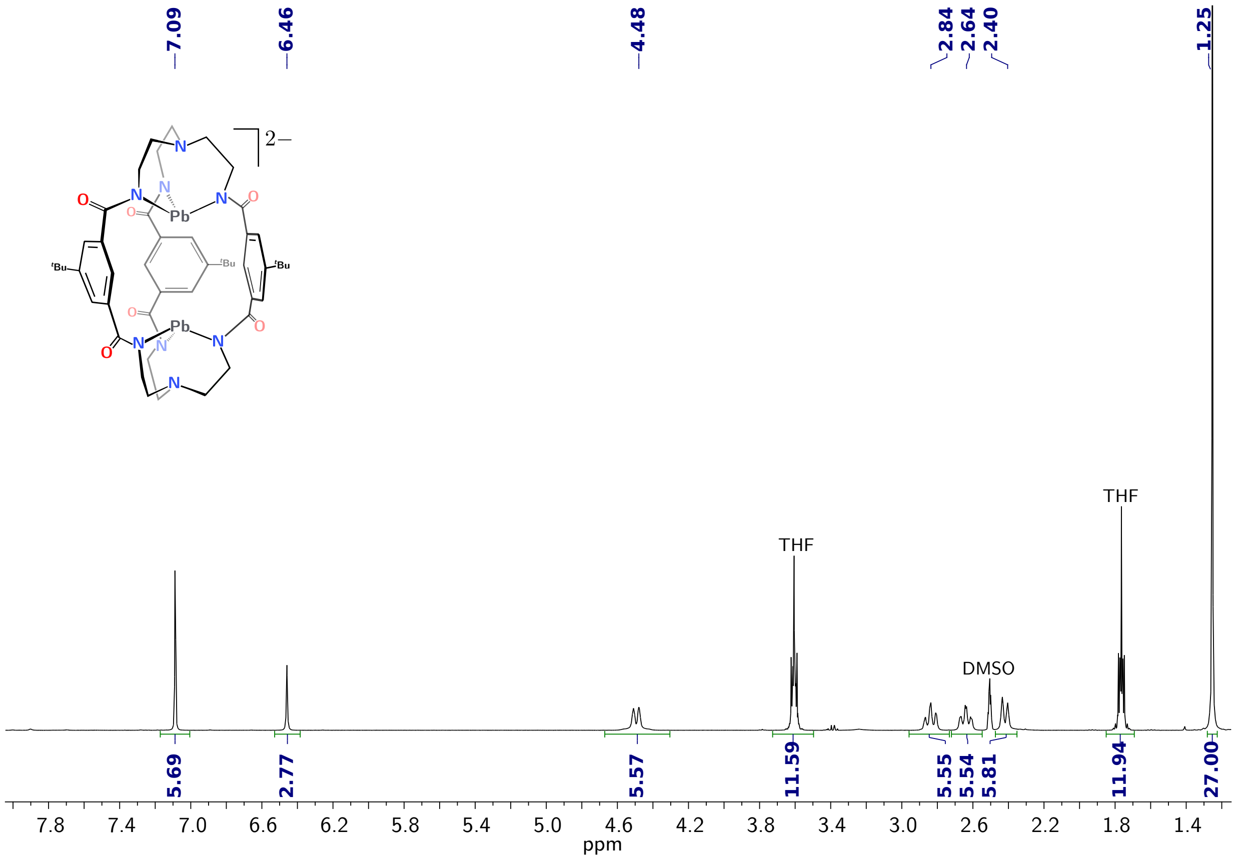

Figure S7: ^{1}H NMR spectrum of $[K_2(THF)][\mathbf{2}]$ in DMSO-d_6 (400.1 MHz, 25 °C).

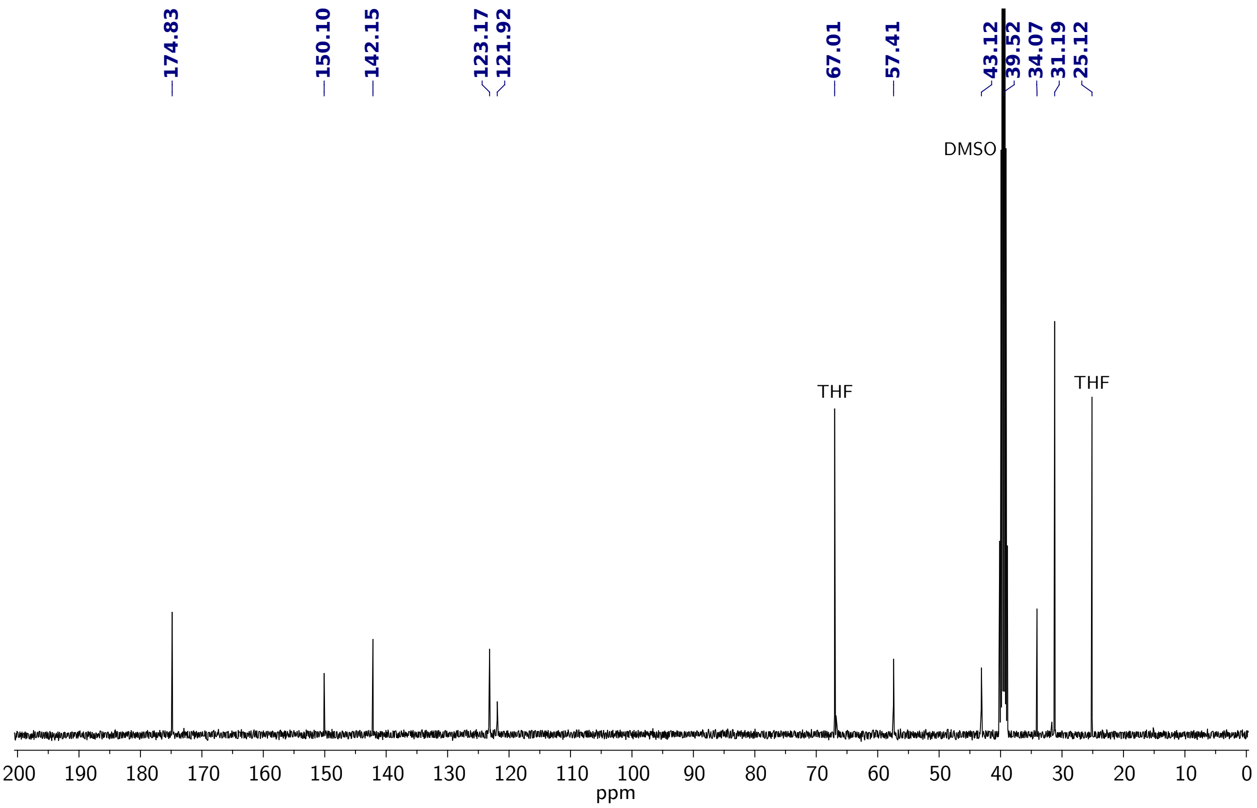

Figure S8: $^{13}C\{^1H\}$ NMR spectrum of $[K_2(THF)][\mathbf{2}]$ in DMSO-d_6 (100.6 MHz, 25 °C).

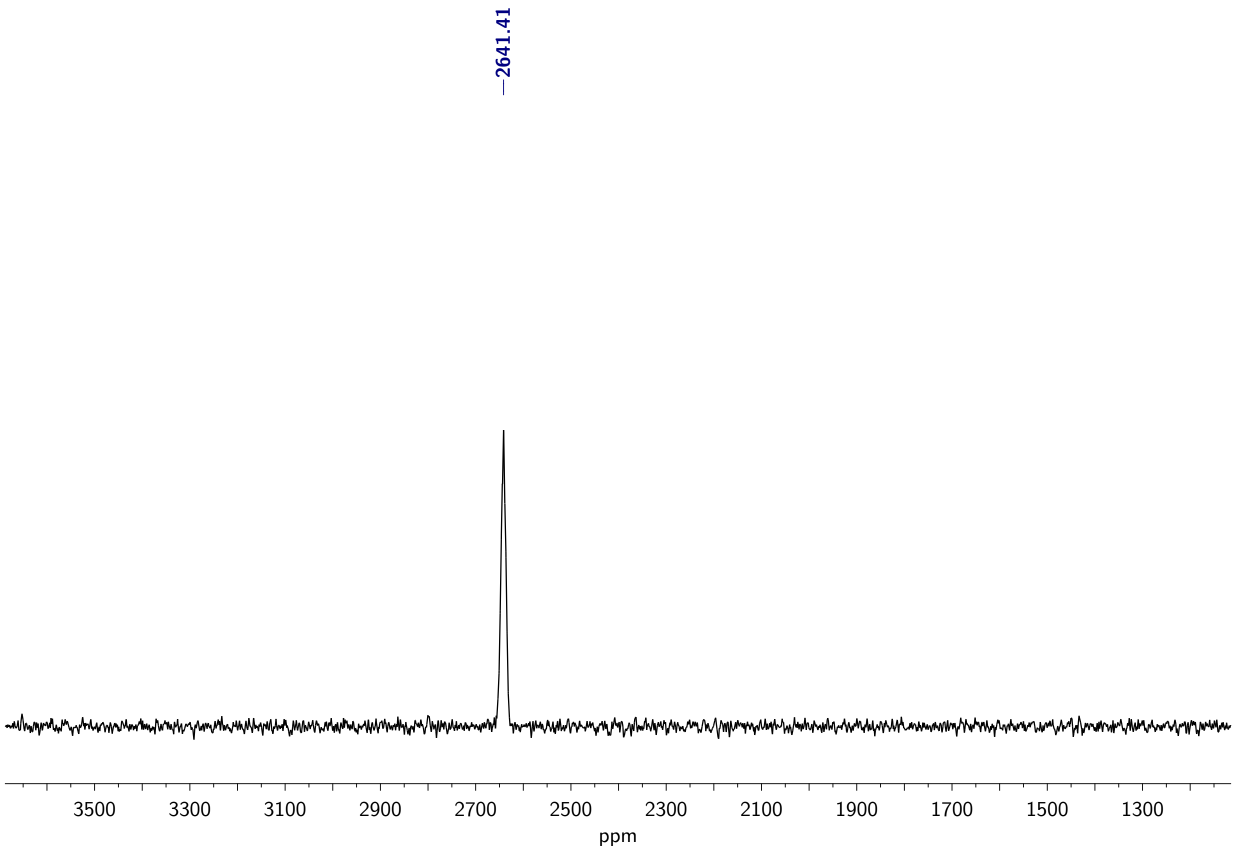

Figure S9: ^{207}Pb NMR spectrum of [K_2(THF)][**2**] in DMSO-d_6 (105 MHz, 25 °C). Relaxation delay: 0.010 sec, acquisition time: 0.020 sec, spectral width: 418,848.2 Hz.

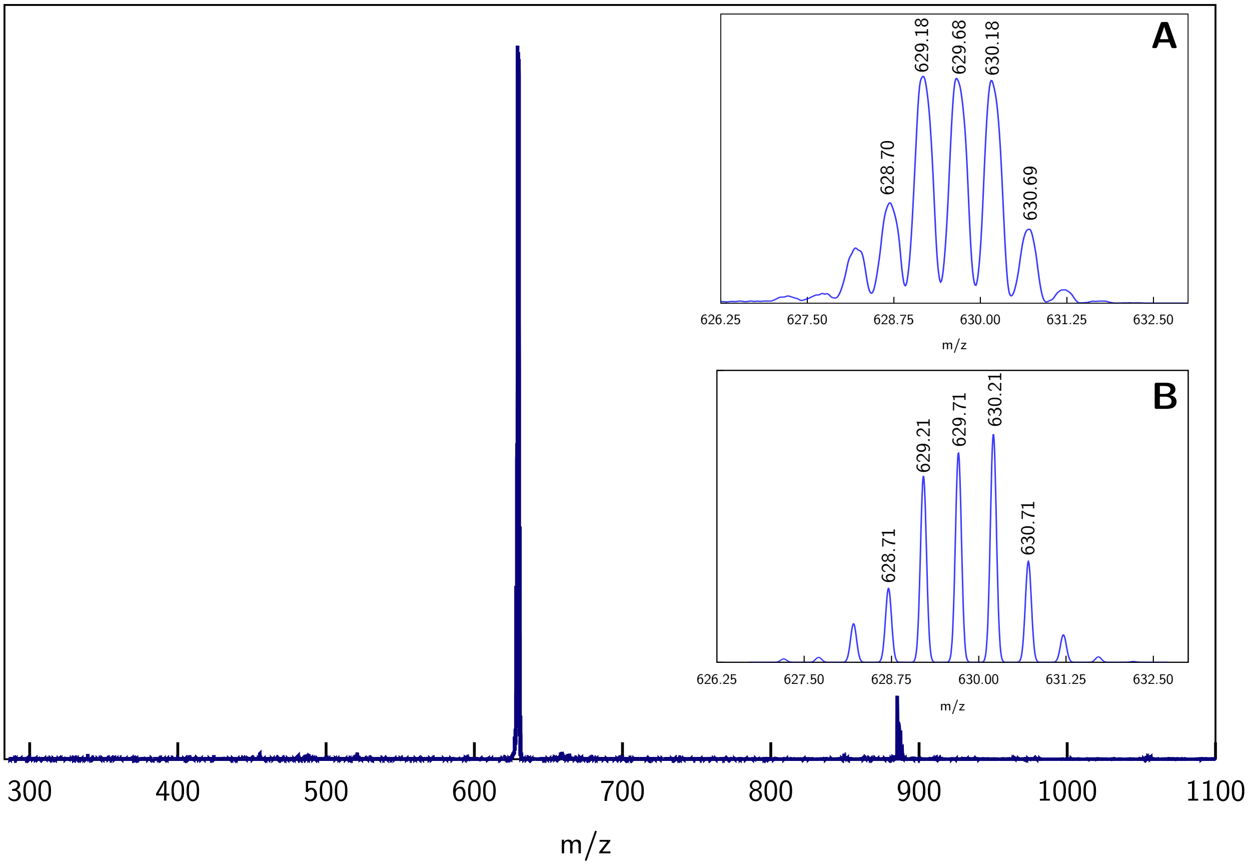

Figure S10: ESI-MS(–) of [K_2(THF)][**2**]. A: zoomed-in spectrum, B: simulated spectrum (DMF, 3200 V).

2.5 Synthesis of [K(Kryptofix-2,2,2)]$_2$[Pb$_2$(*m*BDCA-5t)] ([K(Kryptofix-2,2,2)]$_2$[2])

In the glovebox, THF (2 mL) was added to solid [K_2(THF)][**2**] (100 mg, 0.071 mmol, 1.0 eq) and Kryptofix-2,2,2 (53 mg, 0.14 mmol, 2.0 eq). The colorless suspension was allowed to stir at glovebox temperature (23 °C) for a total of 1 h, at which point the solids were isolated by filtration, washed with THF (1 mL), and dried under reduced pressure affording [K(Kryptofix-2,2,2)]$_2$[**2**] as a colorless powder (yield: 112 mg, 0.054 mmol, 76%). Colorless X-ray quality crystals were grown by vapor diffusion of Et_2O into a saturated DMF solution of [K(Kryptofix-2,2,2)]$_2$[**2**] over the course of 24 h at 23 °C. ^{1}H NMR (DMSO-d_6, 25 °C, 400.1 MHz, δ): 7.09 (d, 6H), 6.46 (t, 3H), 4.48 (d, 6H), 3.55 (s, 24H, Kryptofix-2,2,2), 3.51 (m, 24H, Kryptofix-2,2,2), 2.84 (m, 6H), 2.64 (m, 6H), 2.50 (m, 24H, Kryptofix-2,2,2 overlapping with DMSO solvent), 2.40 (m, 6H), 1.25 (s, 27H) ppm. Anal. Calc'd (found) for $C_{84}H_{132}N_{12}O_{18}K_2Pb_2$: C, 48.26 (48.45); H, 6.36 (6.44); N, 8.04 (7.75). ICP-AES results show a K/Pb ratio of 1.00 $\pm$ 0.01.

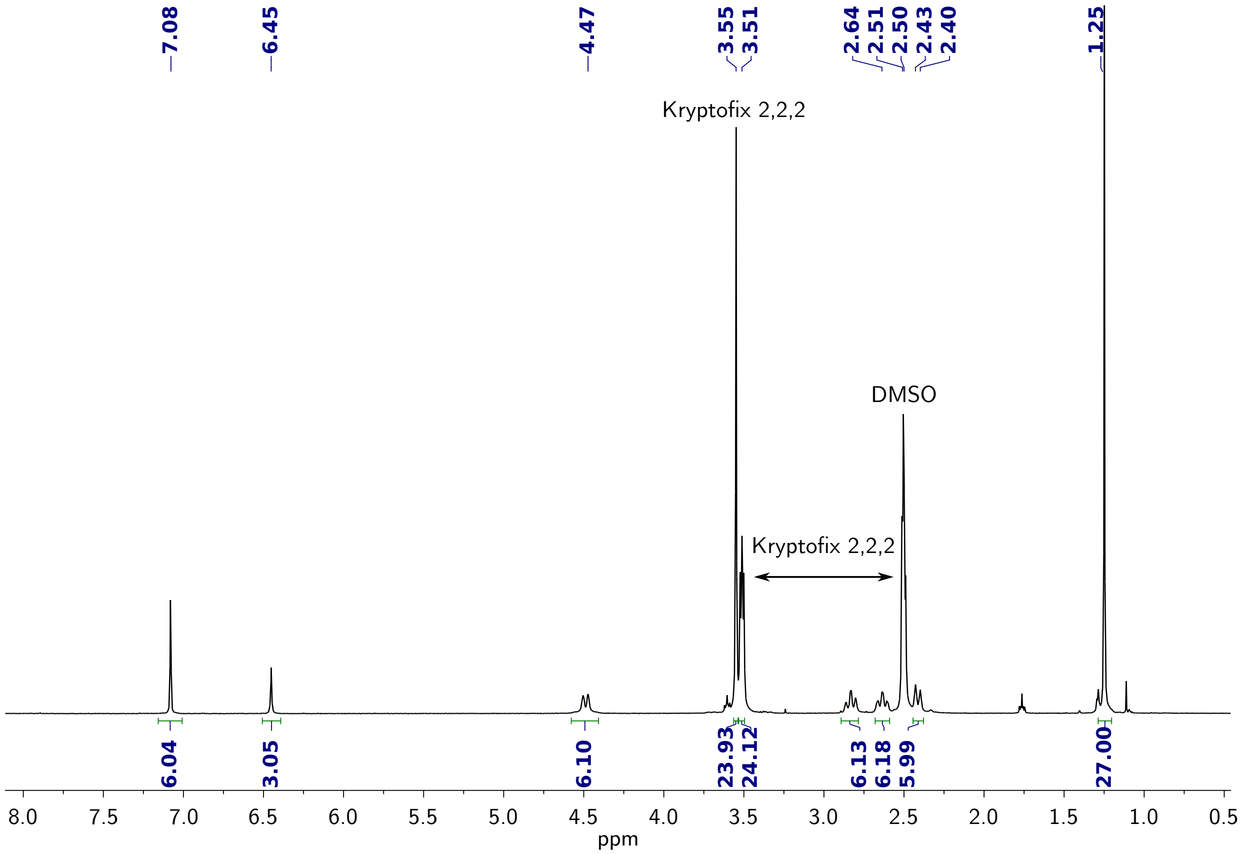

Figure S11: ^{1}H NMR spectrum of [K(Kryptofix-2,2,2)]$_2$[**2**] in DMSO-d_6 (400.1 MHz, 25 °C).

2.6 Synthesis of [K_2(DMF)$_3$][(μ–Se_5)Sn_2(μ–Se)(*m*BDCA-5t)] ([K_2(DMF)$_3$][**3**])

In the glovebox, solid gray selenium (79 mg, 1.0 mmol, 6.2 equiv) was added to a stirring solution of [K_2(THF)][**1**] (200 mg, 0.162 mmol, 1.0 equiv) in DMF (3 mL). The color of the reaction mixture quickly changed from colorless to maroon within 5 min of stirring. The reaction mixture was allowed to stir at glovebox temperature (23 °C) for a total of 6 h, at which point the maroon solution was filtered through a pad of Celite to remove any unreacted selenium. Et_2O (15 mL) was added to the maroon filtrate to precipitate dark red solids from solution. The solids were isolated by filtration on a medium-porosity fritted funnel, washed with Et_2O (10 mL), and dried under reduced pressure affording [K_2(DMF)$_3$][**3**] as a dark red powder (yield: 213 mg, 0.115 mmol, 71%). ^{1}H NMR (DMSO-d_6, 25 °C, 400.1 MHz, δ): 8.02 (t, 1H), 7.95 (DMF, s, 3H), 7.37 (d, 2H), 6.96 (d, 2H), 6.91 (d, 2H), 6.30 (t, 1H), 4.63 (t, 1H), 4.13 (m, 4H), 3.95 (m, 2H), 3.76 (m, 2H), 3.19 (m, 2H), 2.99 (m, 6H), 2.89 (DMF, 9H), 2.82 (d, 2H), 2.73 (DMF, 9H), 2.60 (m, 2H), 2.19 (m, 2H), 1.36 (s, 9H), 1.13 (s, 9H), 1.04 (s, 9H) ppm. ^{13}C{^{1}H} NMR (DMSO-d_6, 25 °C, 100.6 MHz, δ): 177.0, 176.4, 175.4, 162.3 (DMF), 149.0, 145.7, 144.5, 137.9, 137.3, 136.9, 128.2, 127.6, 127.0, 125.9, 124.8, 120.9, 60.2, 55.4, 54.2, 41.6, 38.3, one –*C*H_2– resonance masked by DMSO solvent, 35.8 (DMF), 34.5, 34.0, 33.9, 37.9, 31.1, 30.8, 30.76 (DMF) ppm. ^{119}Sn{^{1}H} NMR (DMSO-d_6, 25 °C, 149.2 MHz, δ): –876.5 ppm. ^{77}Se NMR (DMSO-d_6, 25 °C, 76.3 MHz, δ): 902.7 (s, 1Se), 884.4 (s, 2Se), 789.7 (s, 2Se), 537.2 (s, 1Se). λ_{max} [ε ($M^{-1}cm^{-1}$)]: 452 [4,207], 585 [842] nm. Anal. Calc'd (found) for $C_{57}H_{81}N_{11}O_9K_2Se_6Sn_2$: C, 36.93 (36.74); H, 4.40 (4.25); N, 8.31 (8.55).

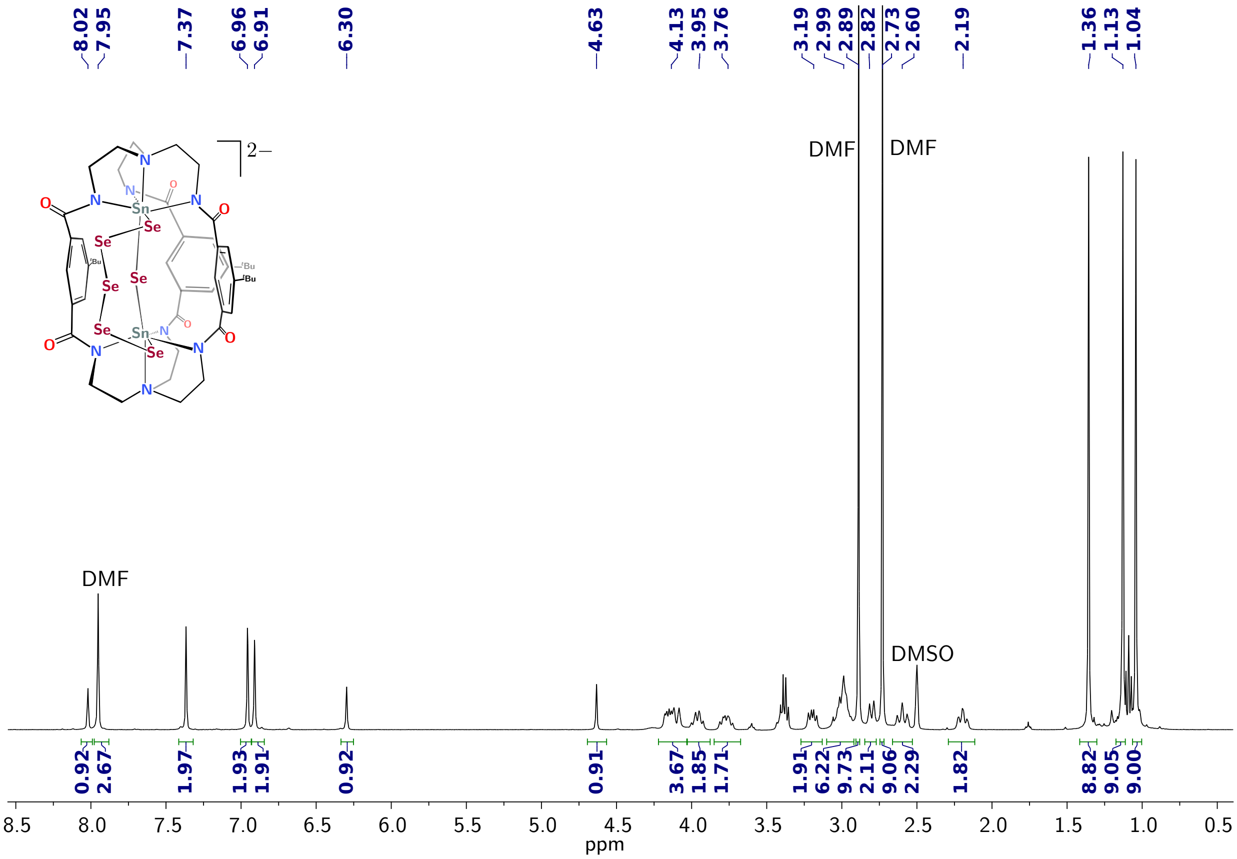

Figure S12: ^{1}H NMR spectrum of [$K_2(DMF)_3$][**3**] in DMSO-d_6 (400.1 MHz, 25 °C).

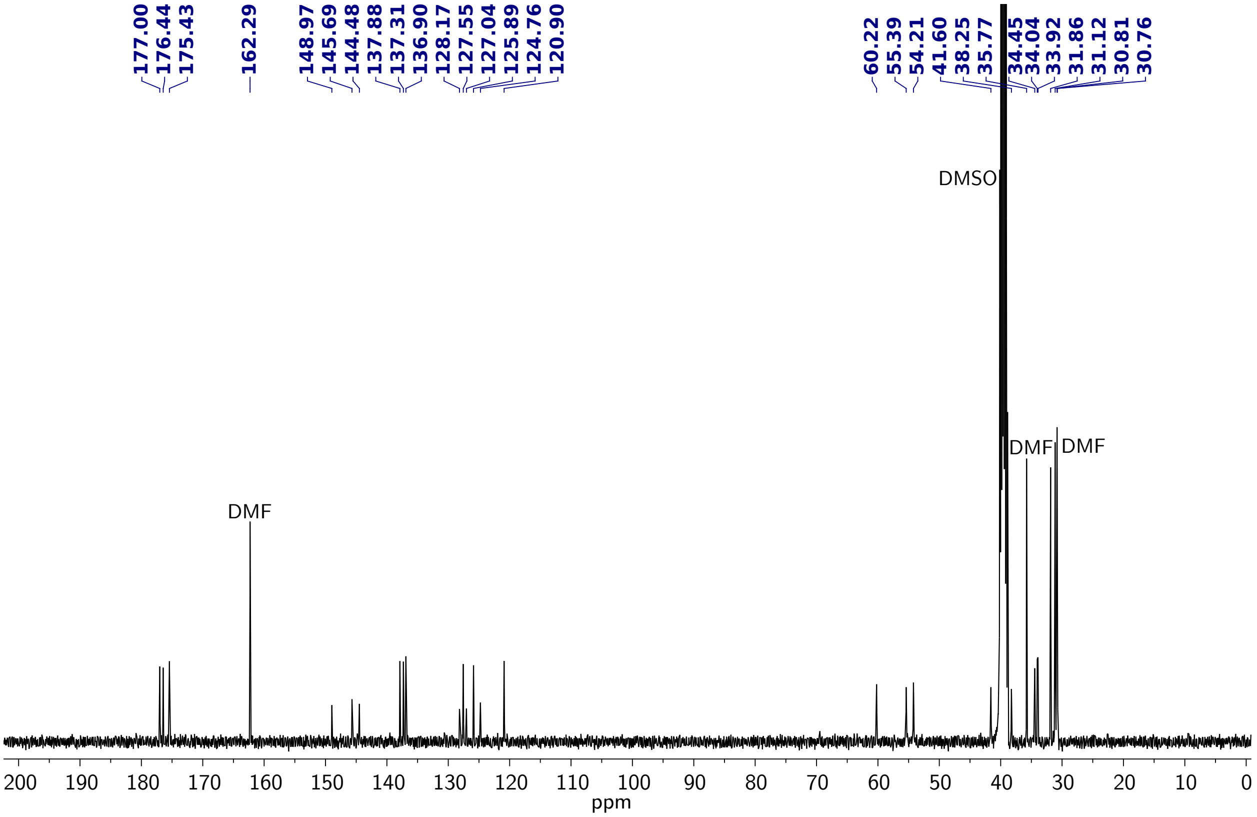

Figure S13: $^{13}C\{^{1}H\}$ NMR spectrum of $[K_2(DMF)_3]$[**3**] in DMSO-d_6 (100.6 MHz, 25 °C).

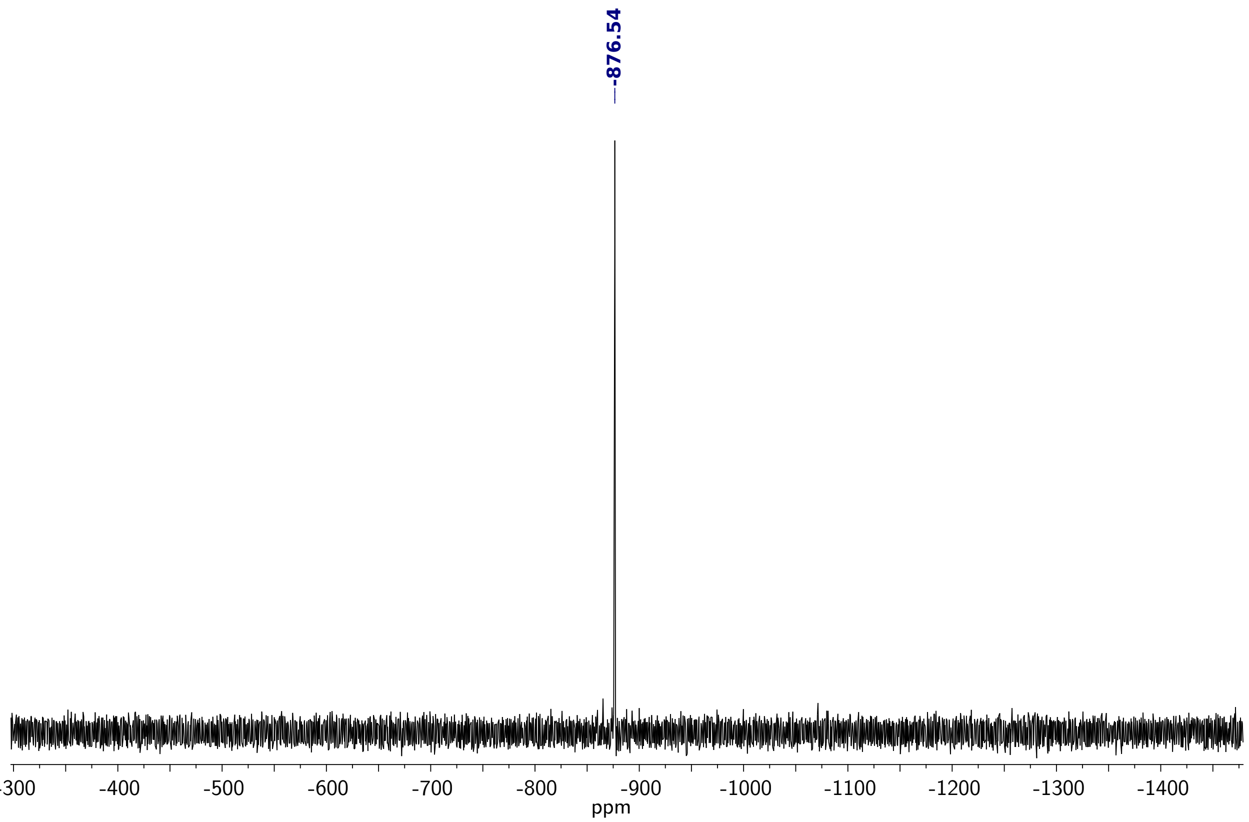

Figure S14: $^{119}Sn\{^{1}H\}$ NMR spectrum of $[K_2(DMF)_3][\mathbf{3}]$ in DMSO-d_6 (186.38 MHz, 25 °C).

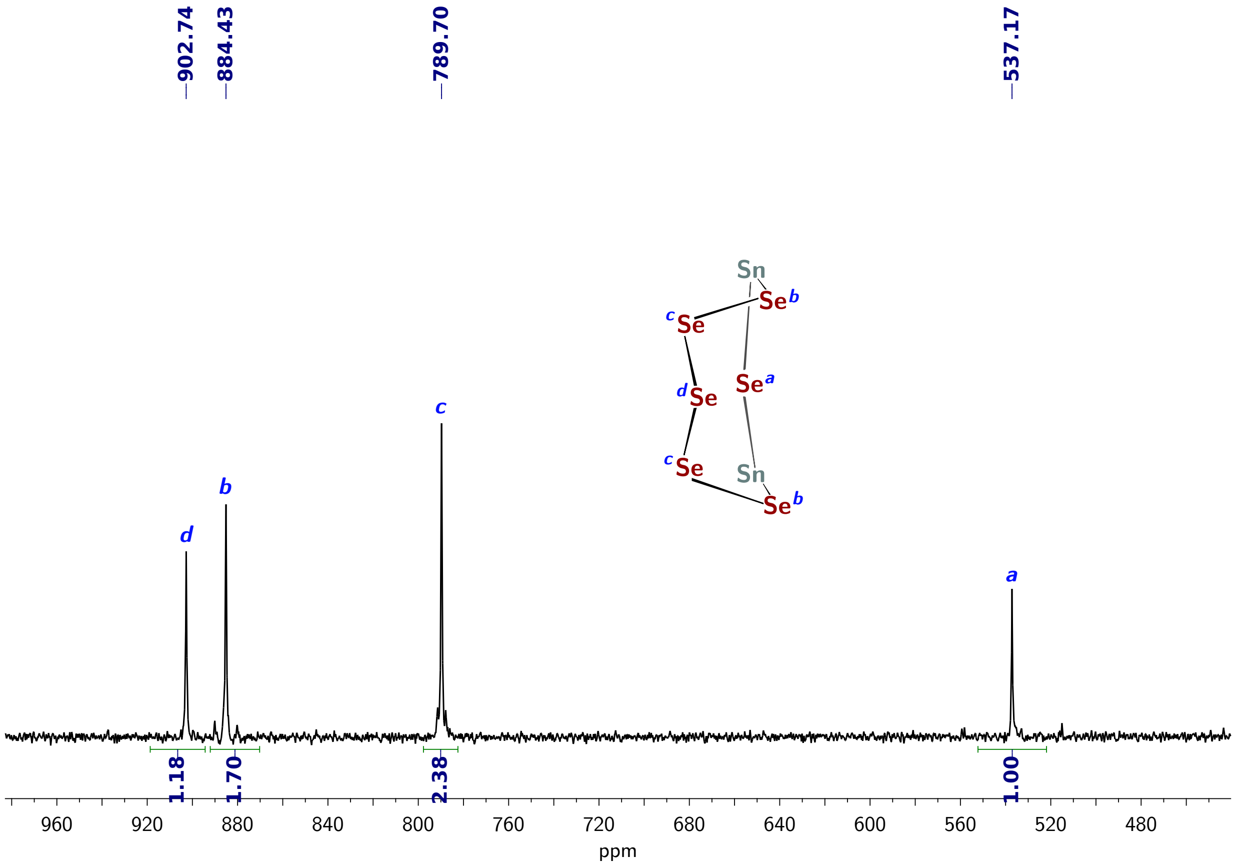

Figure S15: ^{77}Se NMR spectrum of $[K_2(DMF)_3]$[**3**] with peak assignments (DMSO-d_6, 76.3 MHz, 25 °C).

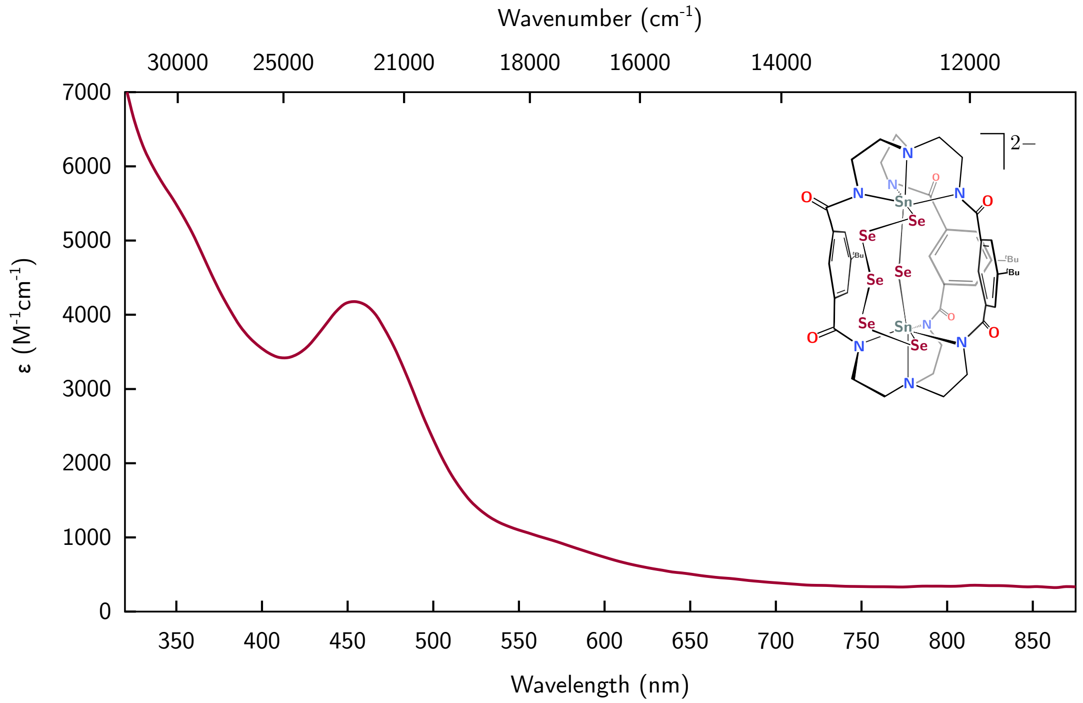

Figure S16: UV-Vis spectrum of $[K_2(DMF)_3]$[**3**] (DMF, 0.26 mM).

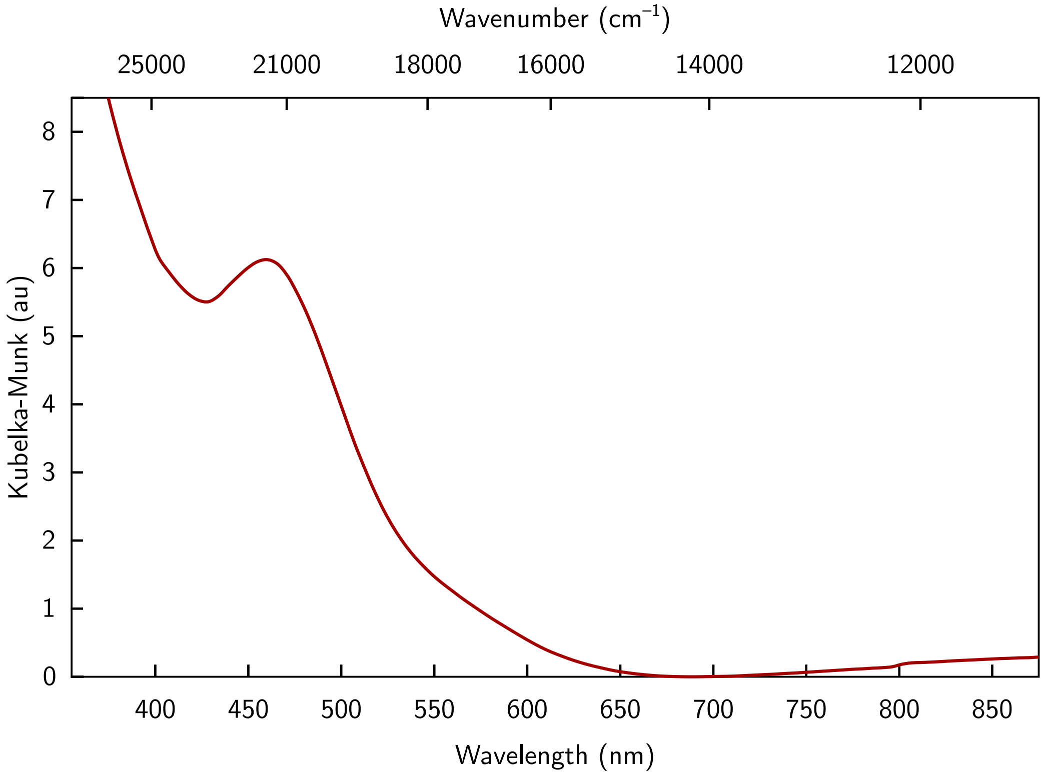

Figure S17: Diffuse reflectance UV-Vis spectrum of $[K_2(DMF)_3]$[**3**].

2.7 Synthesis of $[K_2(DMF)_3][(\mu\text{–}S_5)Sn_2(\mu\text{–}S)(m\mathbf{BDCA\text{-}5t})]$ ($[K_2(DMF)_3][\mathbf{4}]$)

In the glovebox, solid S_8 (31 mg, 0.12 mmol, 0.75 equiv) was added to a stirring solution of $[K_2(THF)][\mathbf{1}]$ (200 mg, 0.162 mmol, 1.00 equiv) in DMF (3 mL). The color of the reaction mixture quickly changed from colorless to deep green within 2 min of stirring. The reaction mixture was allowed to stir at glovebox temperature (23 °C) for a total of 3 h, at which point Et_2O (15 mL) was added to precipitate yellow solids from solution. The solids were isolated by filtration on a medium-porosity fritted funnel, washed with Et_2O (10 mL), and dried under reduced pressure affording $[K_2(DMF)_3][\mathbf{4}]$ as a yellow powder (yield: 160 mg, 0.102 mmol, 63%). ^{1}H NMR (DMSO-d_6, 25 °C, 400.1 MHz, δ): 7.95 (DMF, s, 3H), 7.92 (t, 1H), 7.27 (d, 2H), 7.08 (d, 2H), 6.92 (d, 2H), 6.43 (t, 1H), 5.29 (t, 1H), 4.28 (d, 2H), 4.19 (m, 2H), 3.79 (m, 2H), 3.68 (m, 2H), 3.27 (m, 2H), 3.02 (m, 8H), 2.89 (DMF, 9H), 2.77 (d, 2H), 2.73 (DMF, 9H), 2.57 (m, 2H), 2.13 (m, 2H), 1.30 (s, 9H), 1.09 (s, 9H), 1.07 (s, 9H) ppm. ^{13}C{^{1}H} NMR (DMSO-d_6, 25 °C, 100.6 MHz, δ): 177.3, 177.2, 145.5, 162.3 (DMF), 148.5, 145.9, 143.7, 137.6, 136.6, 135.9, 127.6, 127.5, 127.4, 126.4, 125.4, 120.6, 61.3, 55.2, 54.7, 41.0, 38.2, one –CH$_2$– resonance masked by DMSO solvent, 35.8 (DMF), 34.0, 33.9 (two signals overlapping), 31.6, 31.1, 30.9, 30.8 (DMF) ppm. ^{119}Sn{^{1}H} NMR (DMSO-d_6, 25 °C, 149.2 MHz, δ): –559.7 ppm. λ_{max} [ε ($M^{-1}cm^{-1}$)]: 392 [2,412], 617 nm [2,332]. 25 °C, 0.29 mM in DMF. Anal. Calc'd (found) for $C_{57}H_{81}N_{11}O_9K_2S_6Sn_2$: C, 43.56 (43.12); H, 5.19 (4.99); N, 9.80 (9.40).

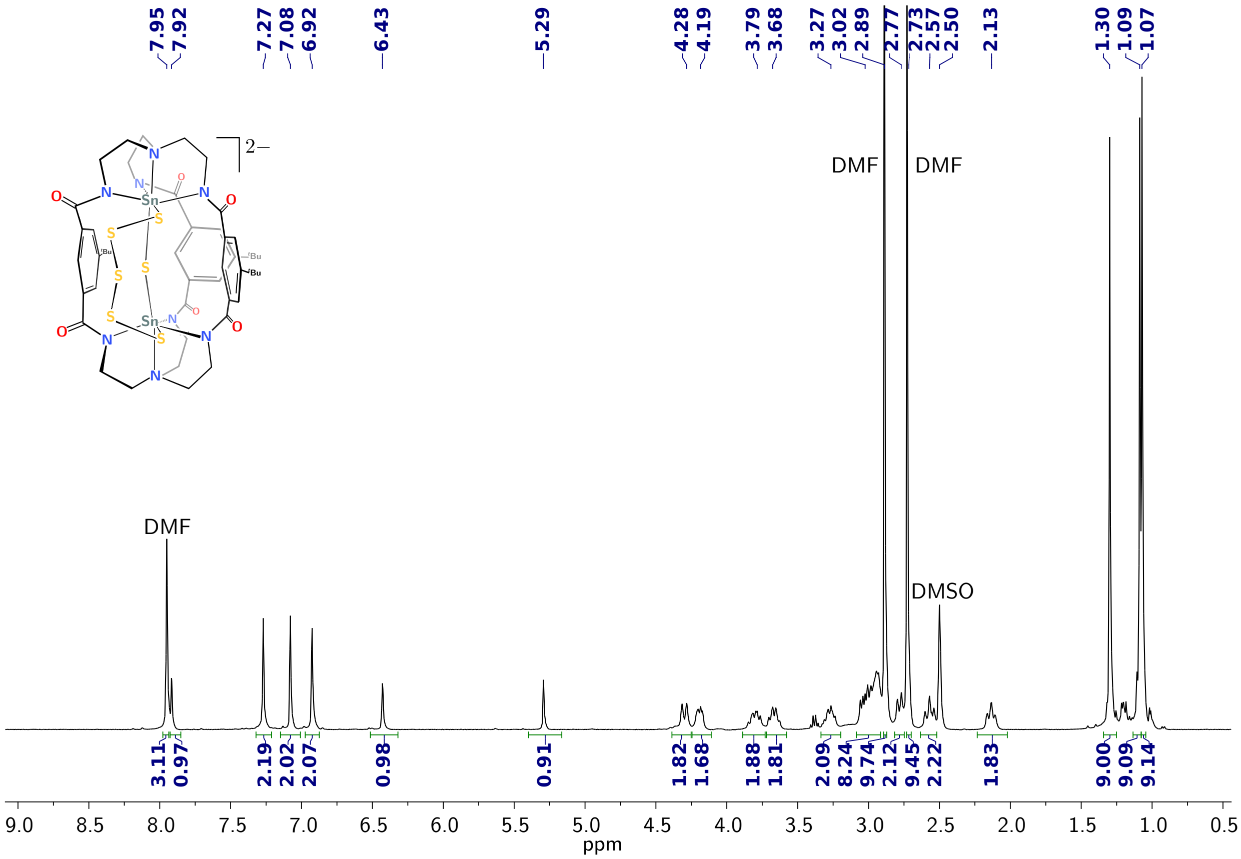

Figure S18: ^{1}H NMR spectrum of $[K_2(DMF)_3]$[**4**] in DMSO-d_6 (400.1 MHz, 25 °C).

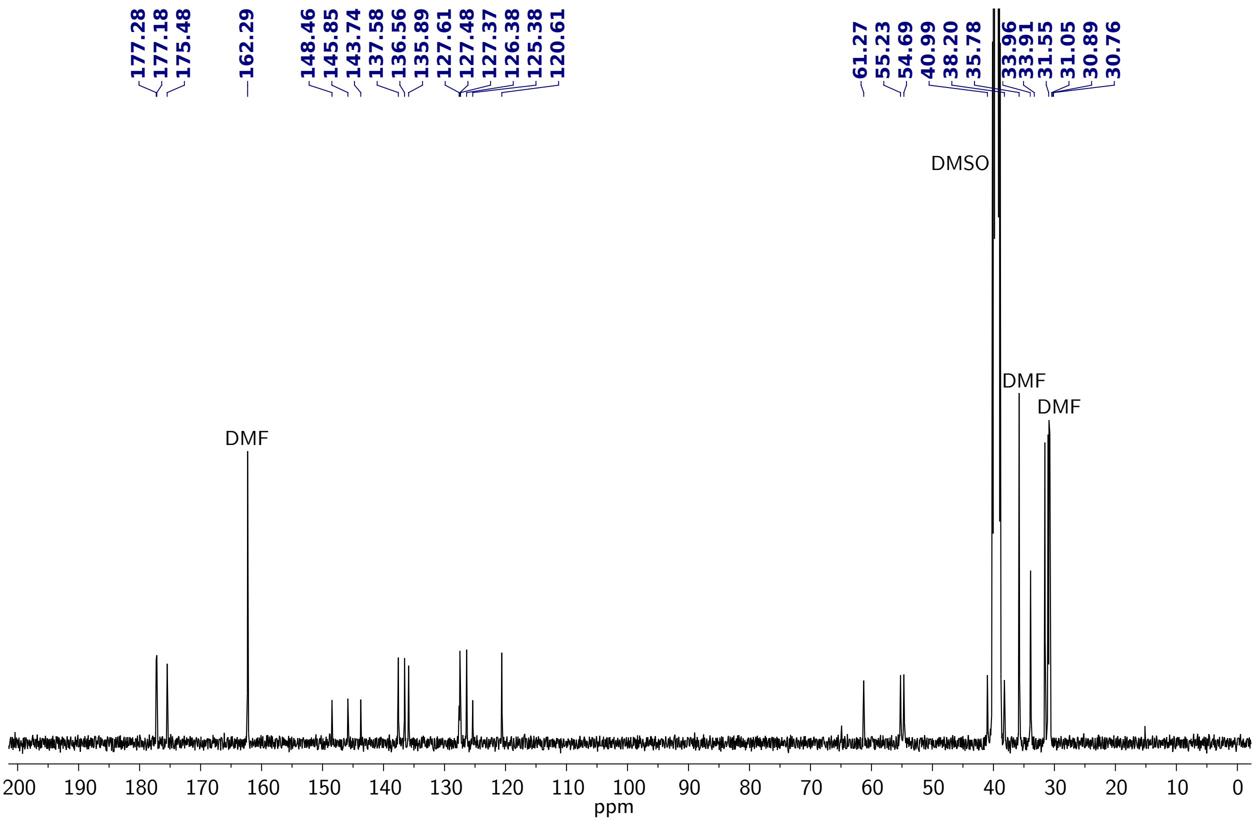

Figure S19: $^{13}C\{^{1}H\}$ NMR spectrum of $[K_2(DMF)_3]$[**4**] in DMSO-d_6 (100.6 MHz, 25 °C).

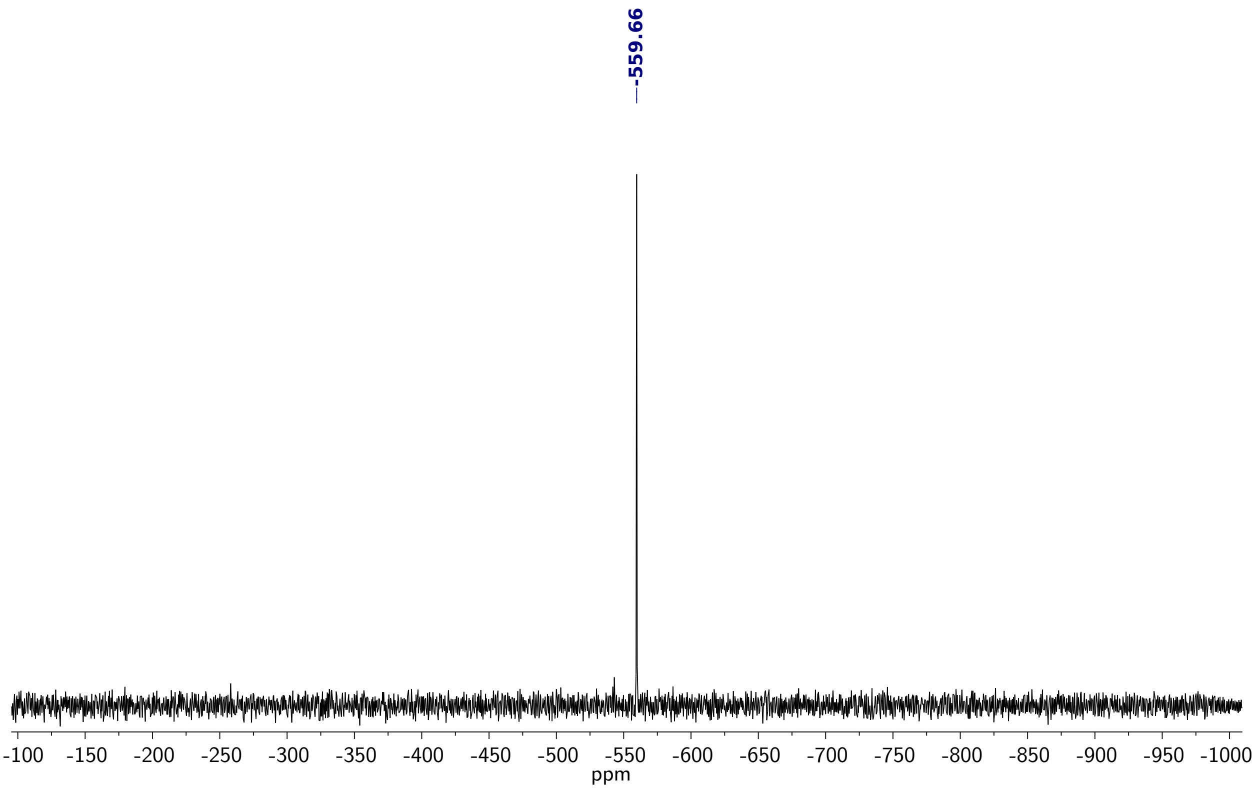

Figure S20: $^{119}Sn\{^{1}H\}$ NMR spectrum of $[K_2(DMF)_3]$[**4**] in DMSO-d_6 (149.2 MHz, 25 °C).

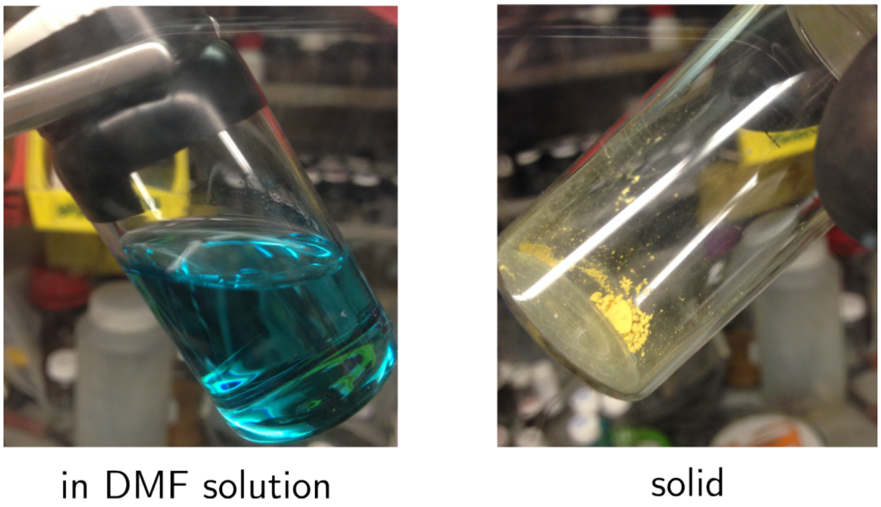

Figure S21: Image of $[K_2(DMF)_3][\mathbf{4}]$ in DMF solution (0.29 mM, left) and as a solid (right).

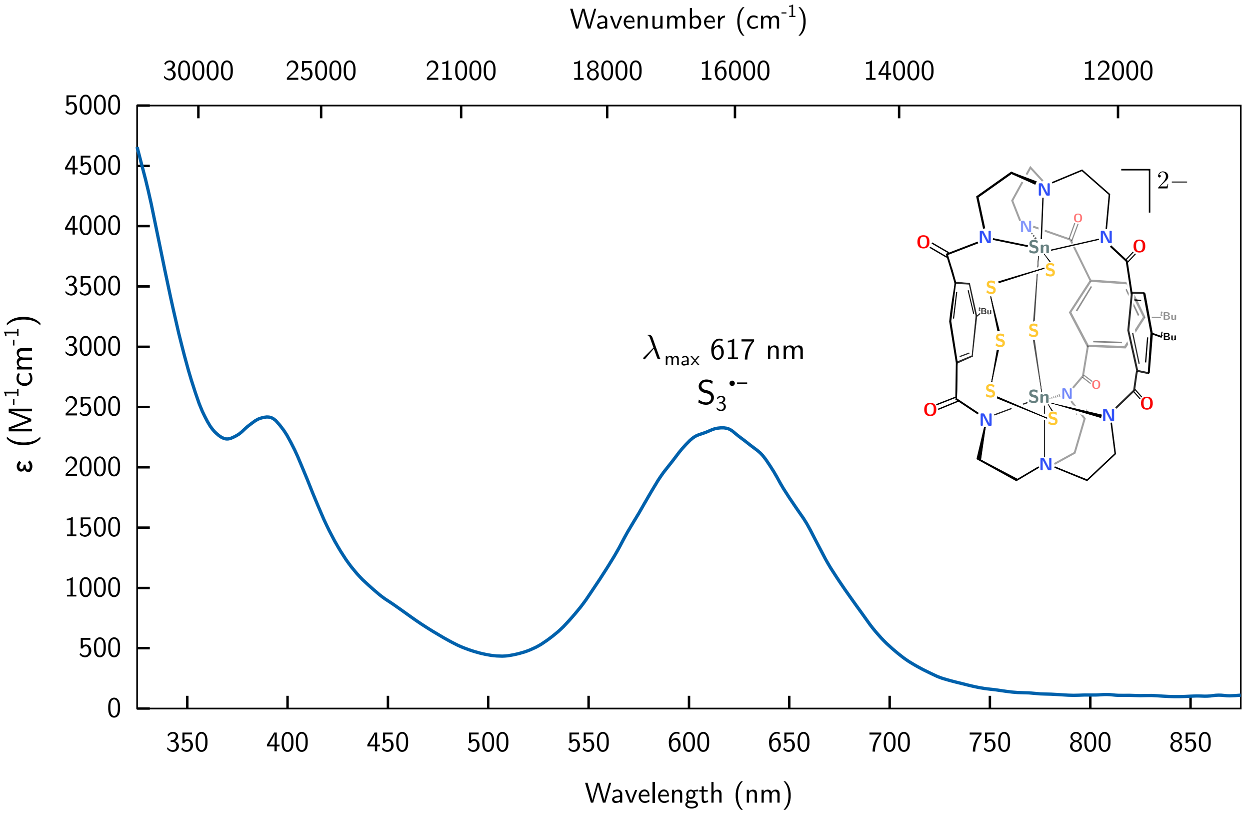

Figure S22: UV-Vis spectrum of $[K_2(DMF)_3][\mathbf{4}]$ (DMF, 25 °C, 0.29 mM).

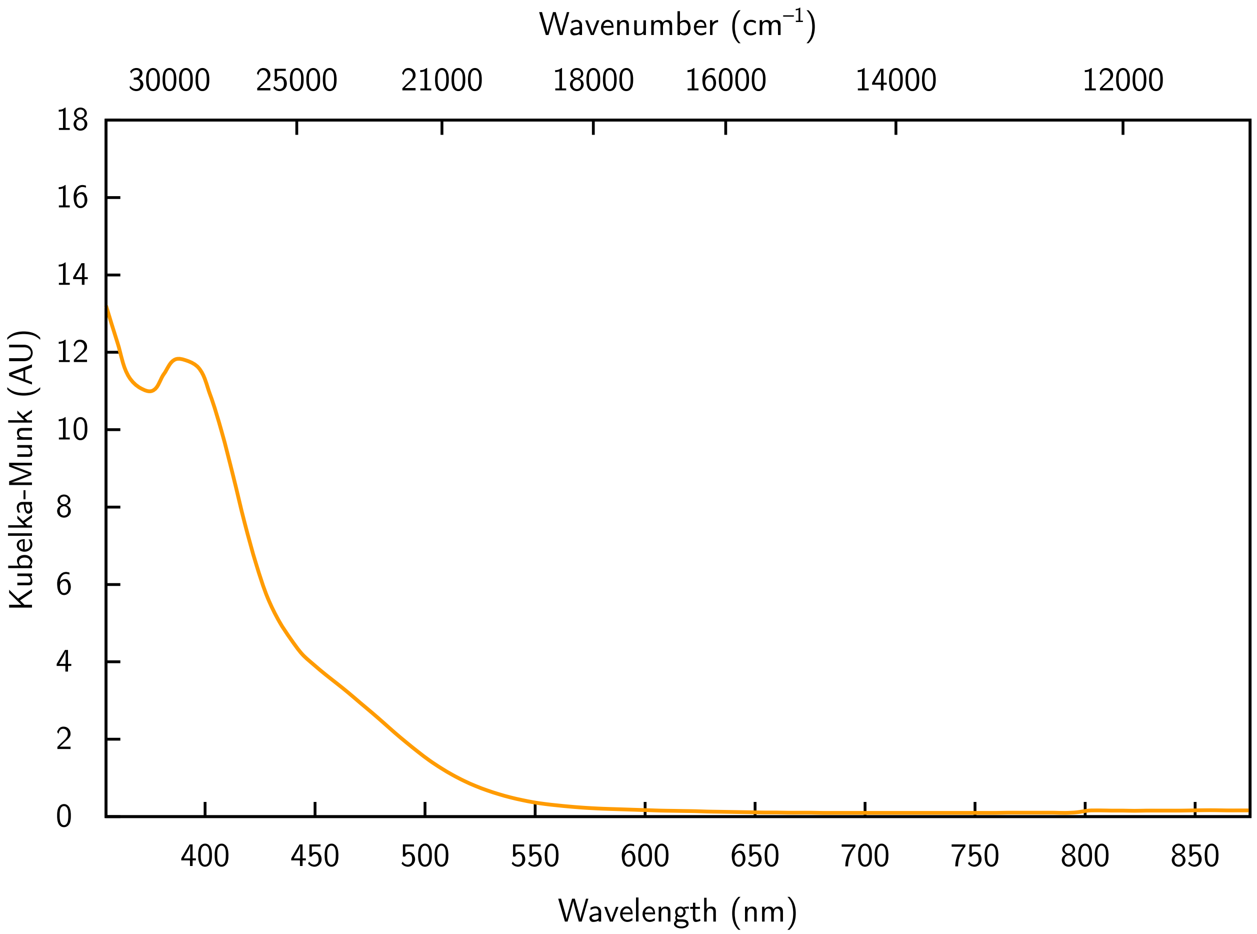

Figure S23: Diffuse reflectance UV-Vis spectrum of $[K_2(DMF)_3]$[**4**].

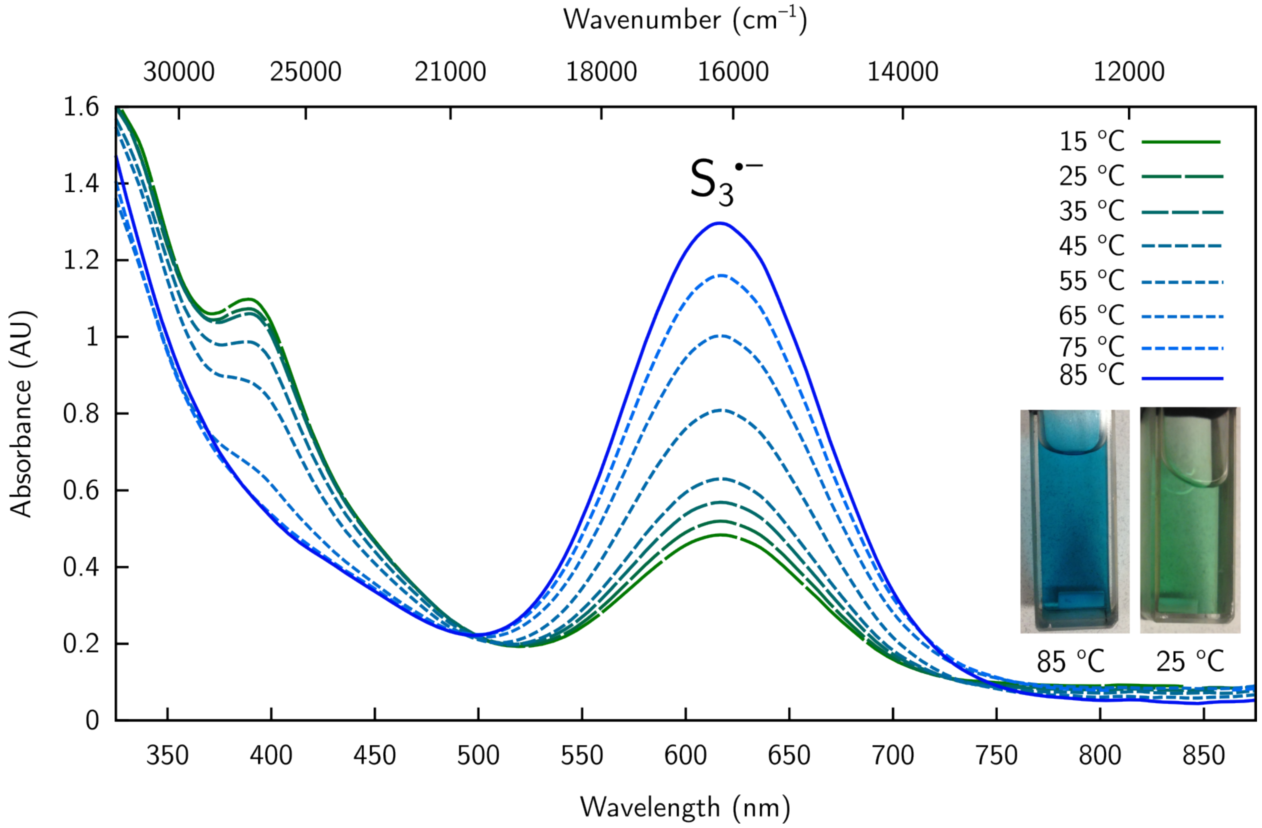

Figure S24: UV-Vis spectra of $[K_2(DMF)_3]$[**4**] in DMF solution (0.3 mM) collected at varying temperature.

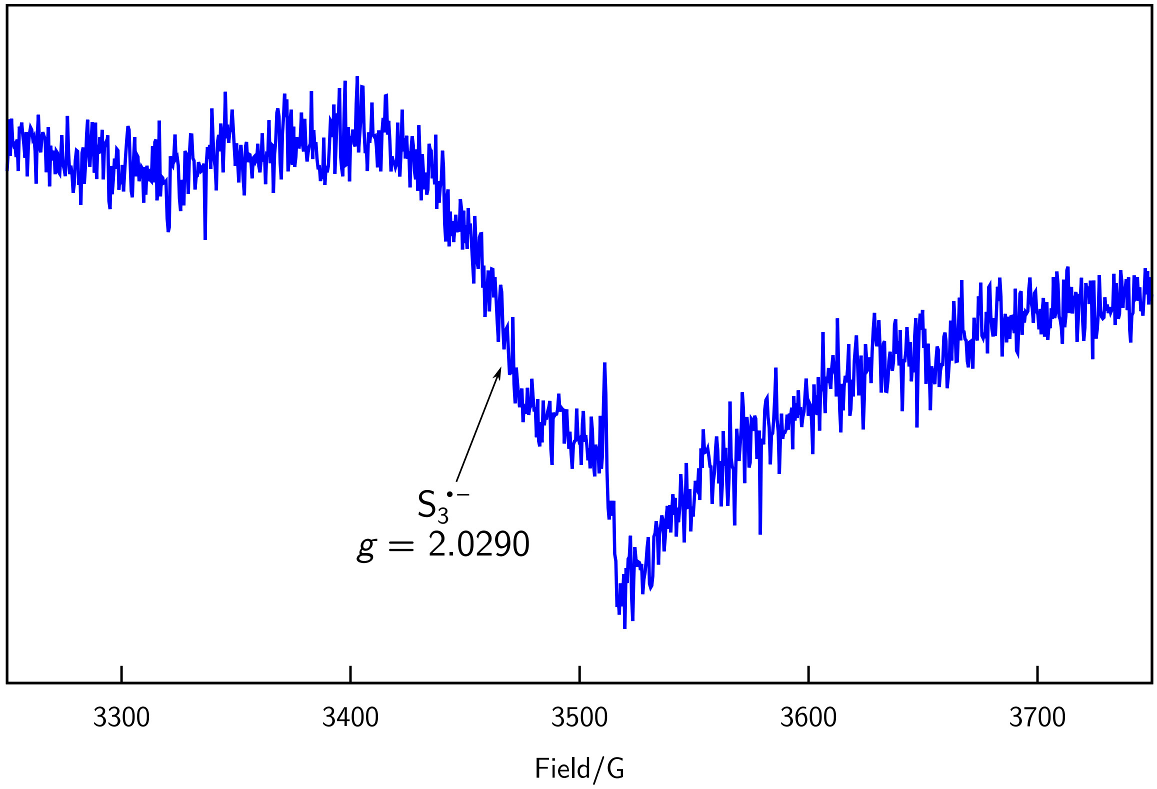

Figure S25: EPR spectrum of a 11 mM DMF solution of $[K_2(DMF)_3]$[**4**] collected at 25 °C.

3 Selenium atom transfer (SeAT) and sulfur atom transfer (SAT) from 3 and 4 to PR_3 (R = Ph, ^{t}Bu, O^{i}Pr)

3.1 SeAT

3.1.1 Treatment of 3 with PPh_3 in DMSO-d_6

To a dark red solution of $[K_2(DMF)_3]$[**3**] (7 mg, 0.004 mmol, 1 eq) in DMSO-d_6 (0.5 mL) was added PPh_3 (6 mg, 0.02 mmol, 6 eq) as a solid. The color of the solution immediately became colorless upon complete dissolution of PPh_3. The solution was transferred to an NMR tube and analyzed by ^{1}H (Figure S26), ^{31}P (Figure S27), and ^{119}Sn (Figure S28) NMR spectroscopy.

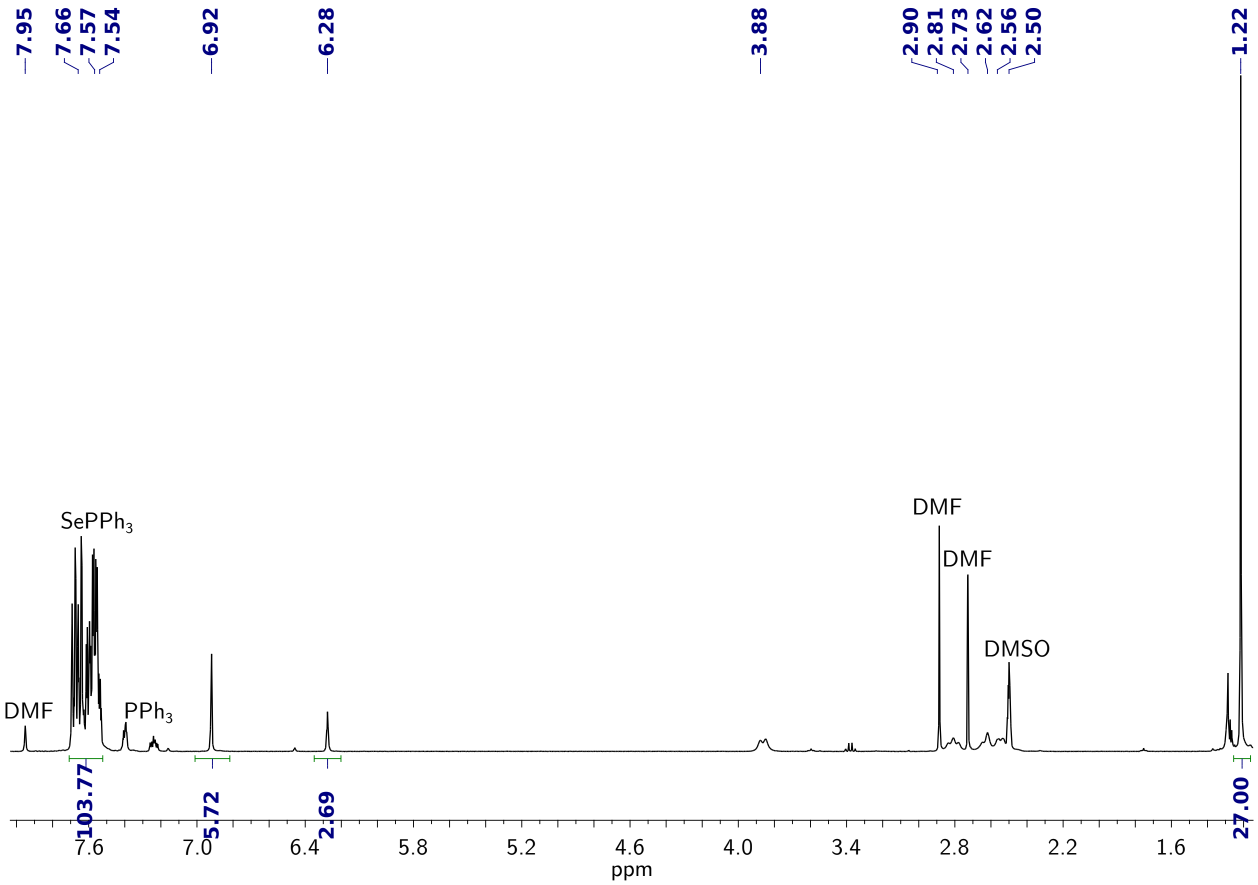

Figure S26: ^{1}H NMR spectrum of **3** treated with PPh_3 showing **1** and $SePPh_3$ (DMSO-d_6, 400 MHz, 25 °C).

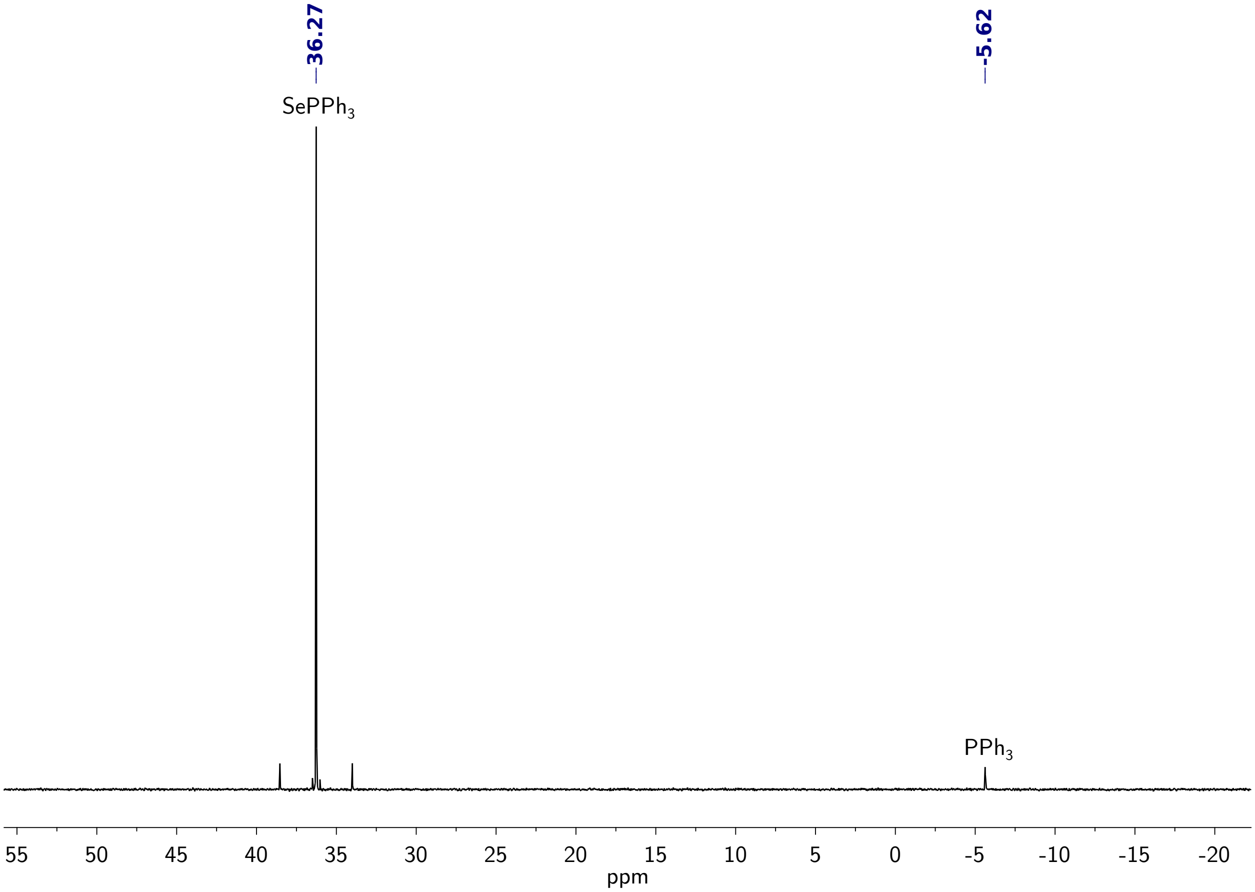

Figure S27: $^{31}P\{^1H\}$ NMR spectrum showing $SePPh_3$ after the treatment of **3** with 6 eq PPh_3 (DMSO-d_6, 162 MHz, 25 °C).

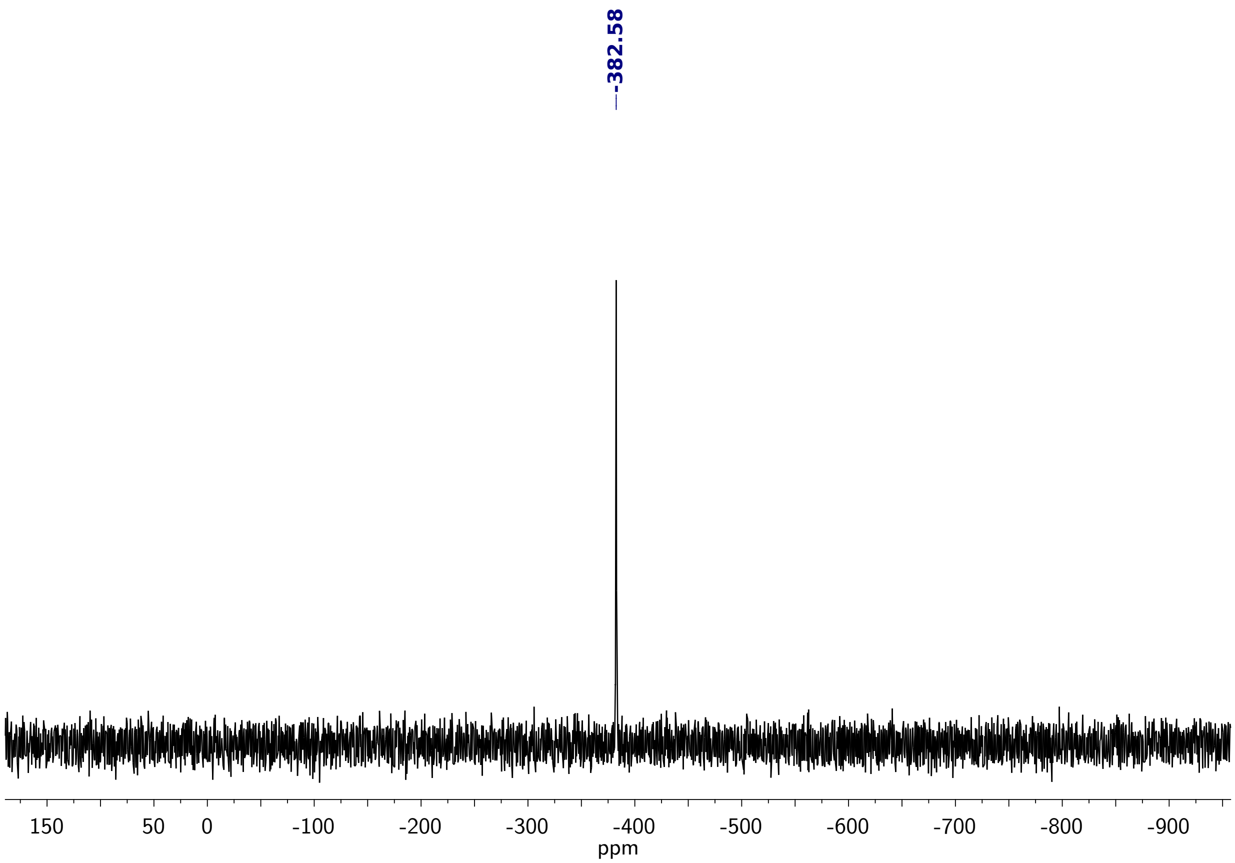

Figure S28: $^{119}Sn\{^1H\}$ NMR spectrum showing **1** after the treatment of **3** with 6 eq PPh_3 (DMSO-d_6, 149.2 MHz, 25 °C).

3.1.2 Monitoring the reaction between 3 and PPh_3 by UV-Vis.

In the glovebox, 2 mL of a freshly prepared 0.26 mM red solution of **3** in DMF (0.52 mmol, 1 eq) was transferred to a 1 cm path length Quartz cuvette. The cuvette was capped, taped, and removed from the glovebox. An initial UV-Vis spectrum was acquired, and then 0.1 mL of a 0.031 M solution of PPh_3 in DMF (3.1 mmol, 6.0 eq) was injected through the septum on the cap of the cuvette. UV-Vis spectra were acquired every 5 seconds for a total of 3 min.

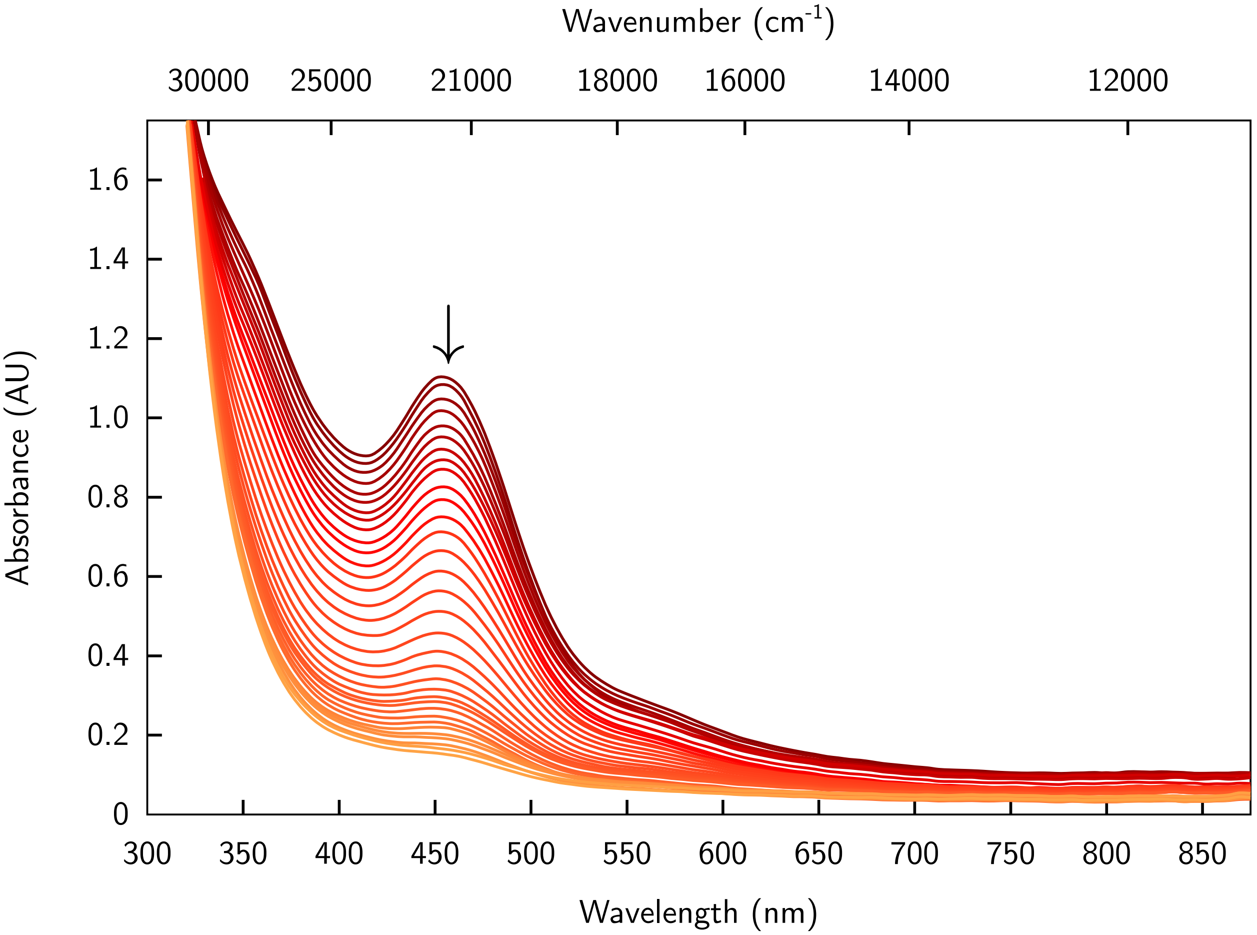

Figure S29: UV-Vis spectra of $[K_2(DMF)_3]$[**3**] upon treatment with PPh_3 monitored over 3 min (DMF, 0.26 mM).

3.1.3 Treatment of 3 with P(^{t}Bu)$_3$ in DMSO-d_6

To a dark red solution of [K_2(DMF)$_3$][**3**] (7 mg, 0.004 mmol, 1 eq) in DMSO-d_6 (0.5 mL) was added P(^{t}Bu)$_3$ (4 mg, 0.02 mmol, 6 eq) as a solid. The color of the solution gradually became colorless over the course of 15 min upon complete dissolution of P(^{t}Bu)$_3$. The solution was transferred to an NMR tube and analyzed by ^{1}H (Figure S30), and ^{31}P (Figure S31) NMR spectroscopy after an additional 30 min.

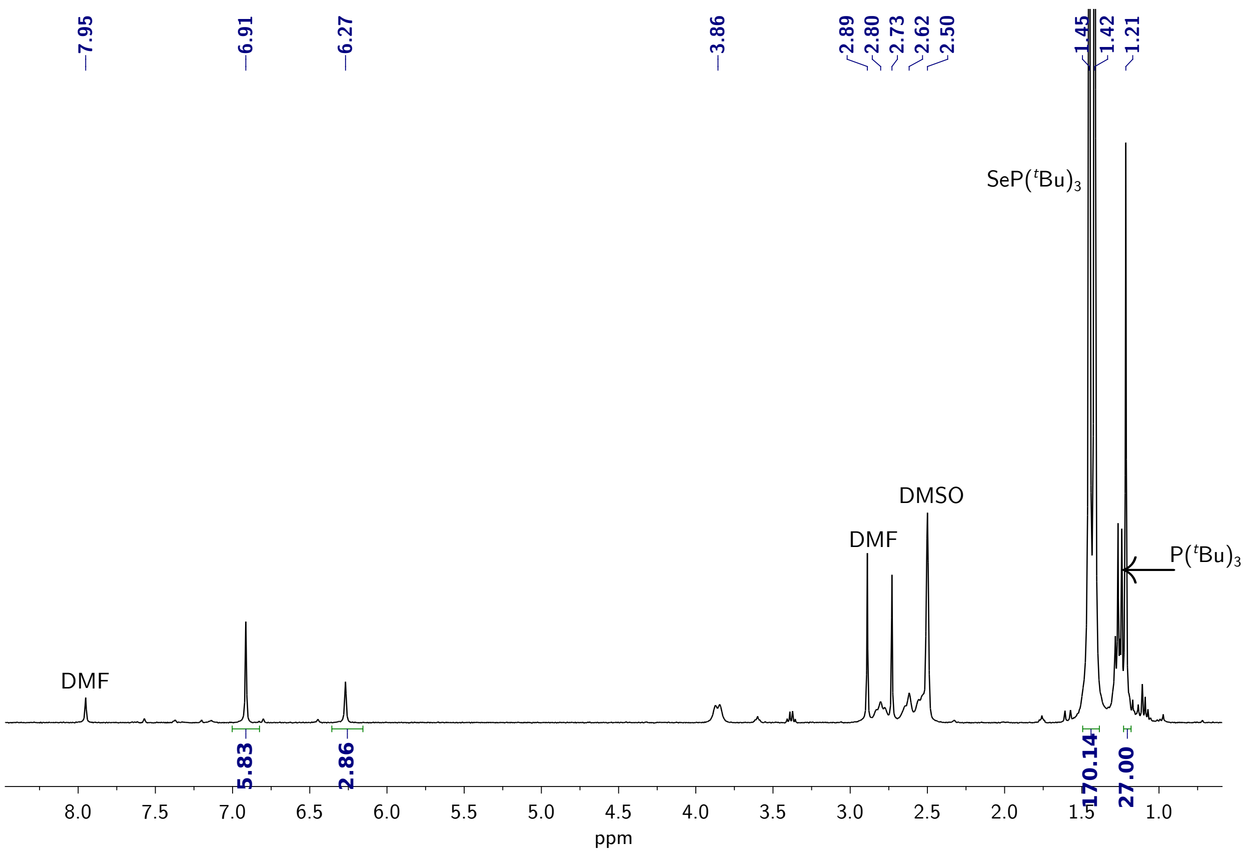

Figure S30: ^{1}H NMR spectrum of **3** treated with P(^{t}Bu)$_3$ showing **1** and SeP(^{t}Bu)$_3$ (DMSO-d_6, 400 MHz, 25 °C).

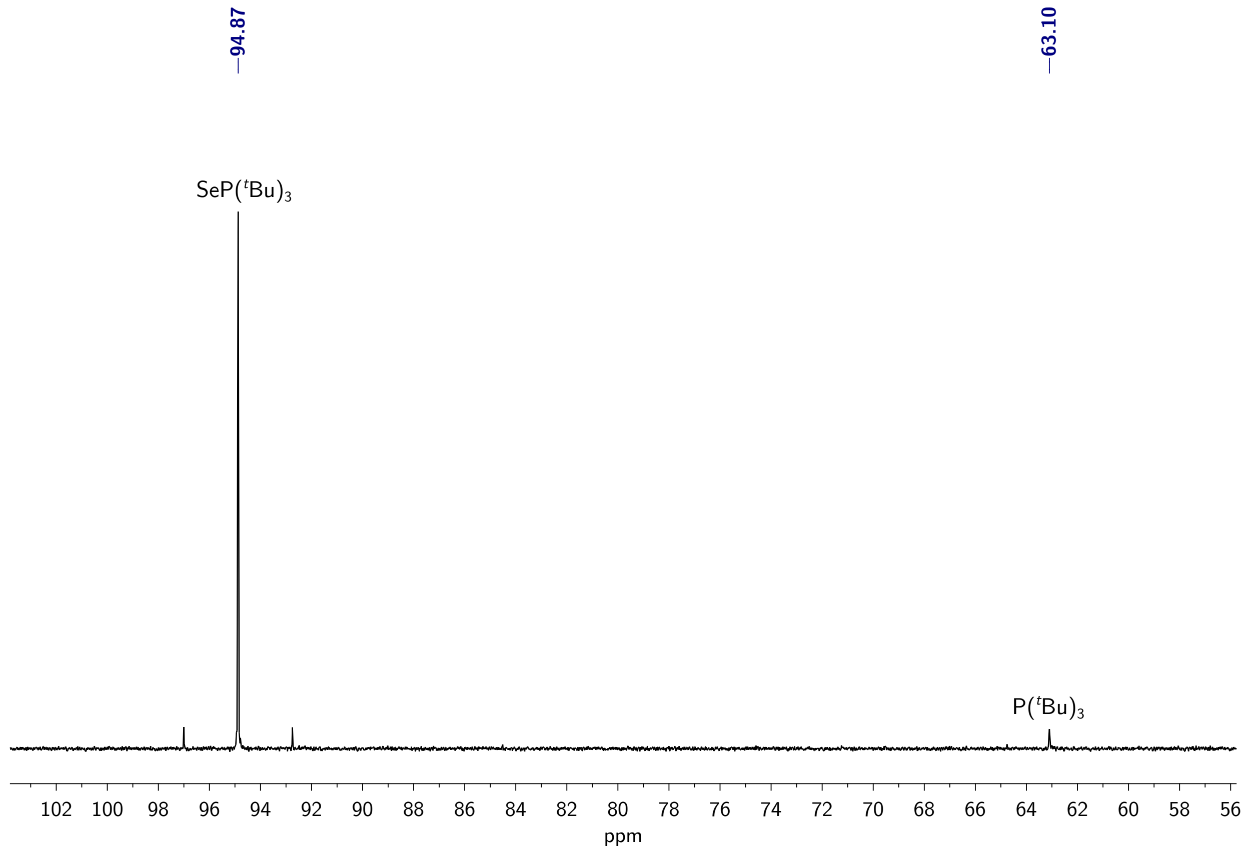

Figure S31: $^{31}P\{^{1}H\}$ NMR spectrum showing SeP(^{t}Bu)$_3$ after the treatment of **3** with 6 eq P(^{t}Bu)$_3$ (DMSO-d_6, 162 MHz, 25 °C).

3.1.4 Treatment of 3 with $P(O^iPr)_3$ in DMSO-d_6

To a dark red solution of $[K_2(DMF)_3]$[**3**] (8 mg, 0.004 mmol, 1 eq) in DMSO-d_6 (0.5 mL) was added $P(O^iPr)_3$ (7 μL, 0.03 mmol, 6.5 eq) via 10 μL syringe. The reaction mixture immediately became colorless upon complete addition. The solution was transferred to an NMR tube and analyzed by ^{1}H (Figure S32), and ^{31}P (Figure S33) NMR spectroscopy.

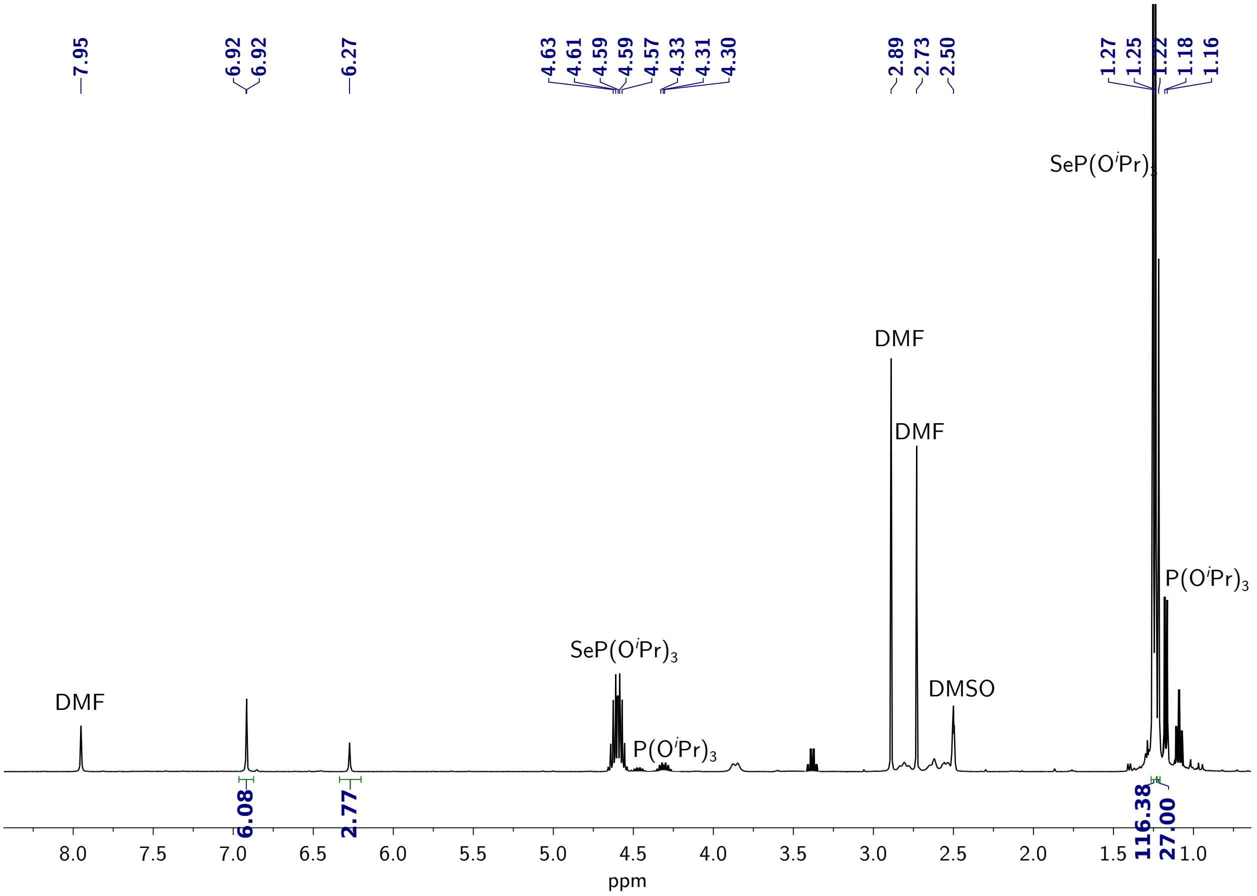

Figure S32: ^{1}H NMR spectrum of **3** treated with $P(O^iPr)_3$ showing **1** and $SeP(O^iPr)_3$ (DMSO-d_6, 400 MHz, 25 °C).

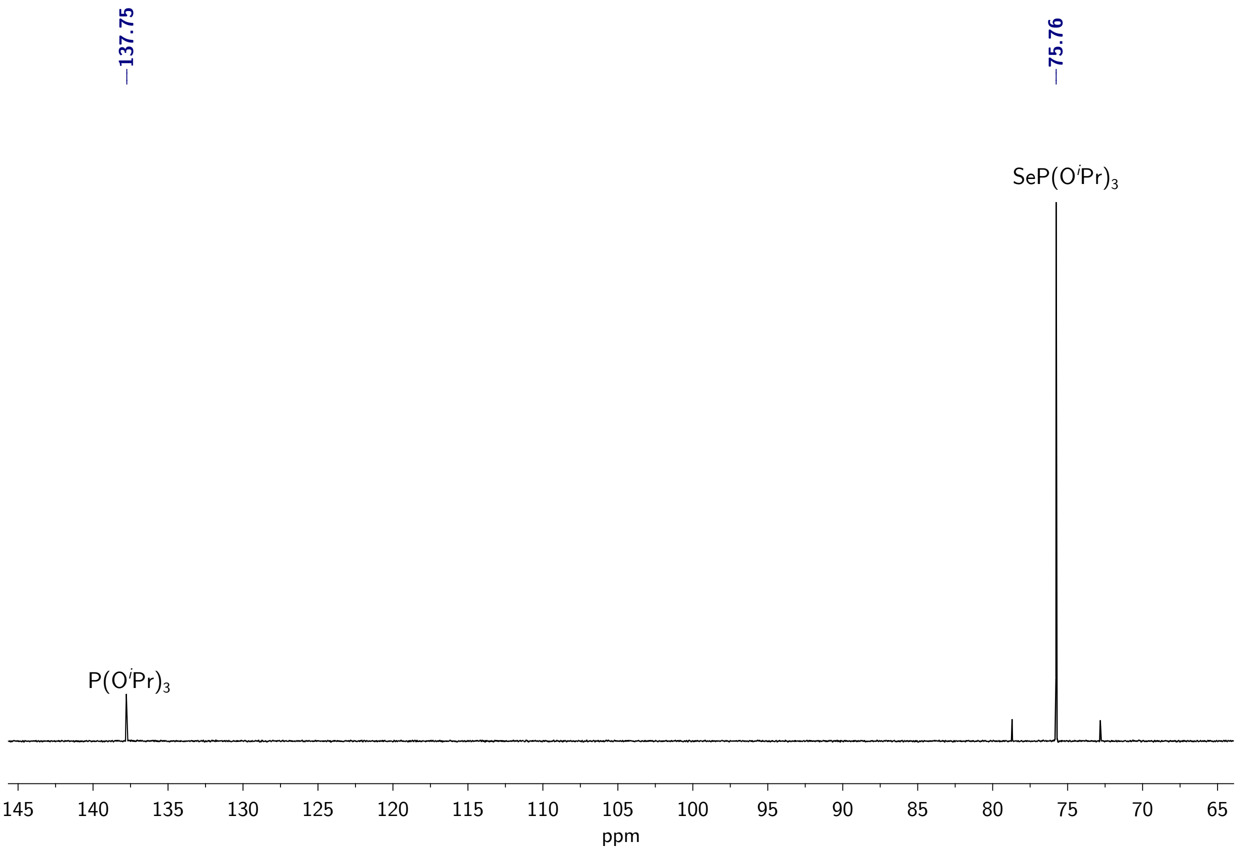

Figure S33: $^{31}P\{^1H\}$ NMR spectrum showing $SePPh_3$ after the treatment of **3** with 6 eq $P(O^iPr)_3$ (DMSO-d_6, 162 MHz, 25 °C).

3.2 SAT

3.2.1 Treatment of 4 with PPh_3 in DMSO-d_6

To a bright green solution of $[K_2(DMF)_3][\mathbf{4}]$ (7 mg, 0.005 mmol, 1 eq) in DMSO-d_6 (0.5 mL) was added PPh_3 (7 mg, 0.03 mmol, 6 eq) as a solid. The reaction mixture immediately became colorless upon complete dissolution of PPh_3. The solution was transferred to an NMR tube and analyzed by ^1H (Figure S34), ^{31}P (Figure S35), and ^{119}Sn (Figure S36) NMR spectroscopy.

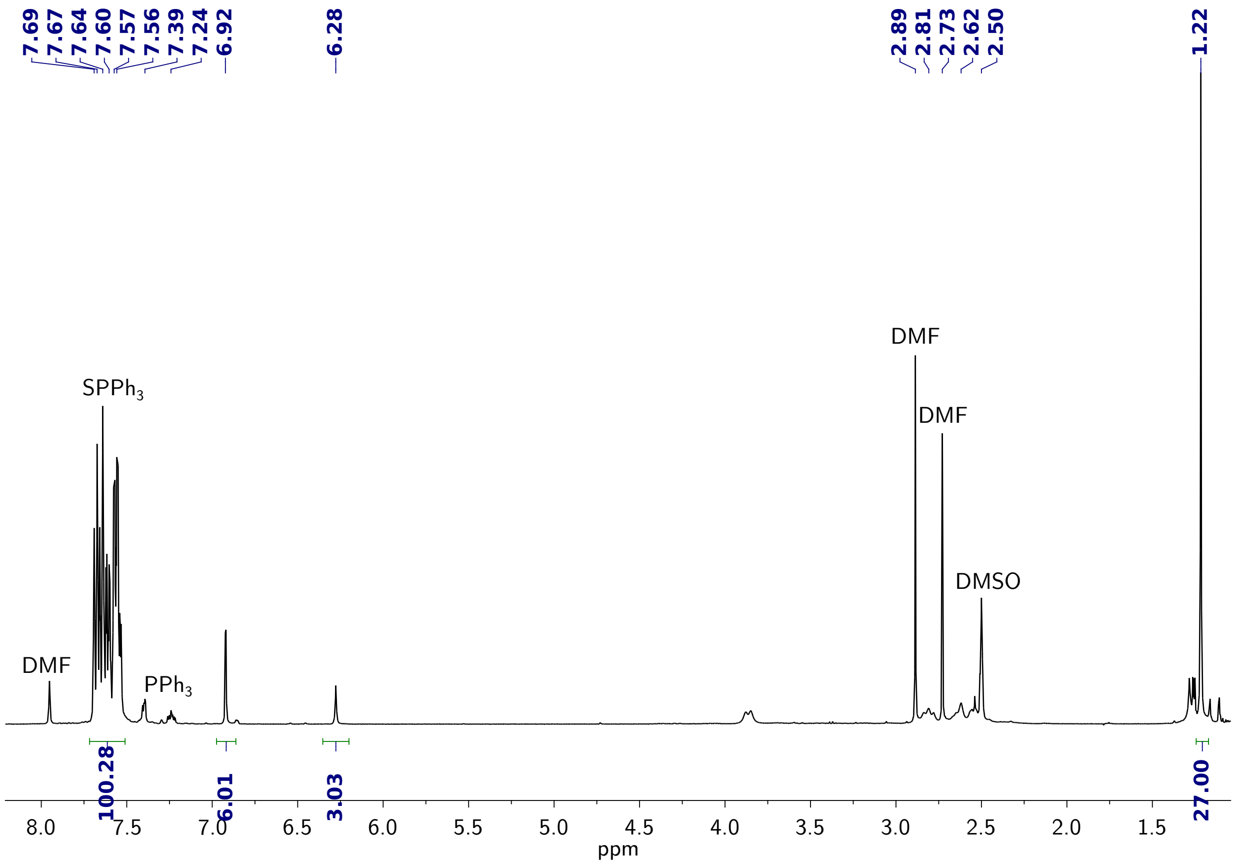

Figure S34: ^1H NMR spectrum of **4** treated with PPh_3 showing **1** and $SPPh_3$ (DMSO-d_6, 400 MHz, 25 °C).

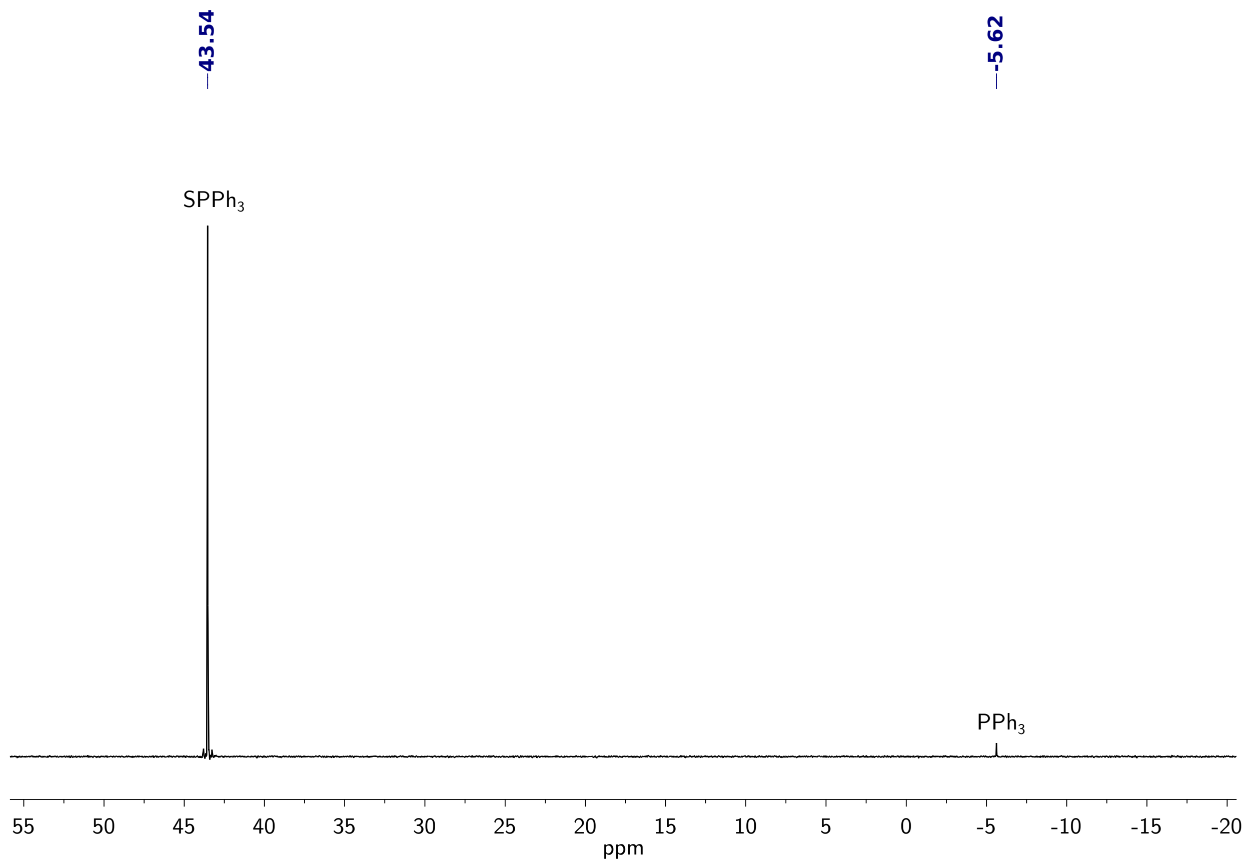

Figure S35: $^{31}P\{^{1}H\}$ NMR spectrum showing $SPPh_3$ after the treatment of **4** with 6 eq PPh_3 (DMSO-d_6, 162 MHz, 25 °C).

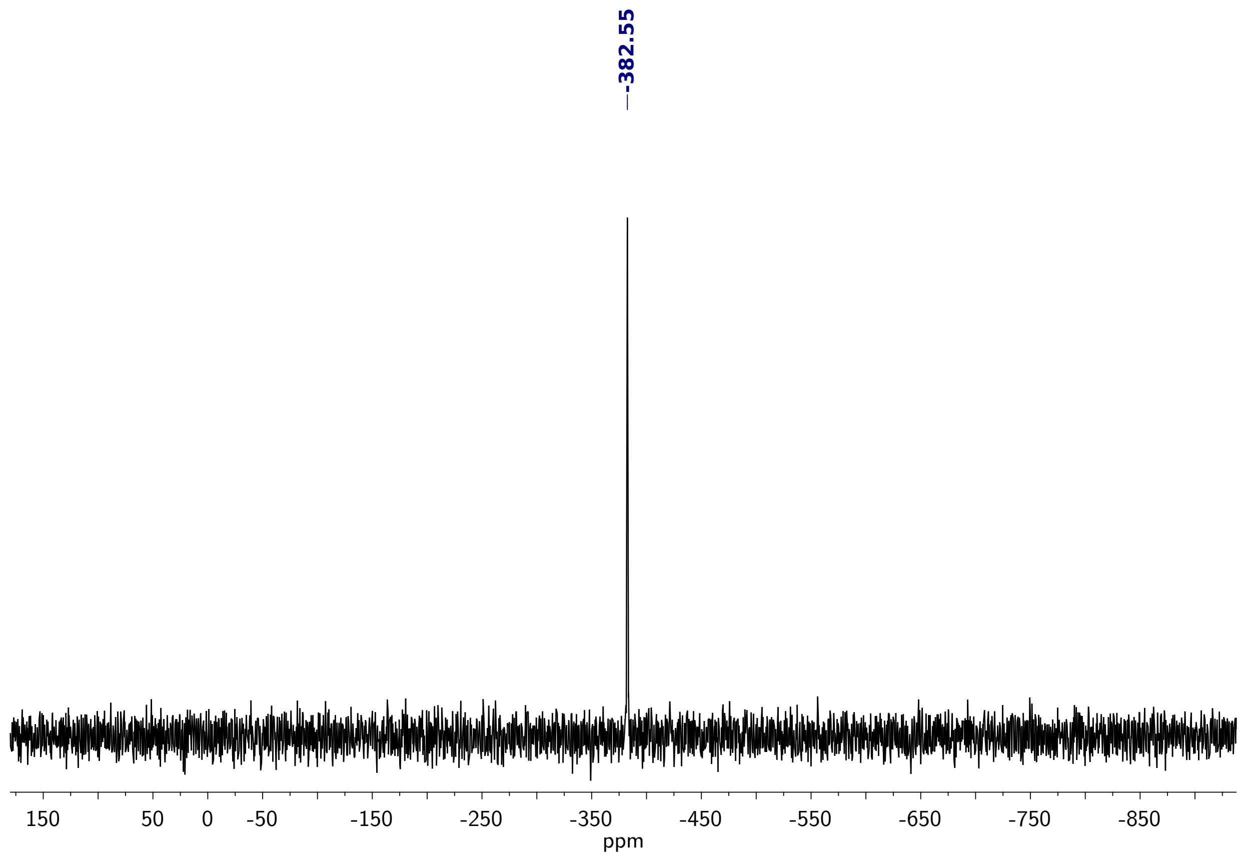

Figure S36: $^{119}Sn\{^1H\}$ NMR spectrum showing **1** after the treatment of **4** with 6 eq PPh_3 (DMSO-d_6, 149.2 MHz, 25 °C).

3.2.2 Monitoring the reaction between 4 and PPh_3 by UV-Vis

In the glovebox, 2 mL of a freshly prepared blue 0.29 mM solution of **4** in DMF (0.58 mmol, 1 eq) was transferred to a 1 cm path length Quartz cuvette. The cuvette was capped, taped, and removed from the glovebox. An initial UV-Vis spectrum was acquired, and then 0.1 mL of a 0.031 M solution of PPh_3 in DMF (3.5 mmol, 6.0 eq) was injected through the septum on the cap of the cuvette. UV-Vis spectra were acquired every 5 seconds for a total of 10 min.

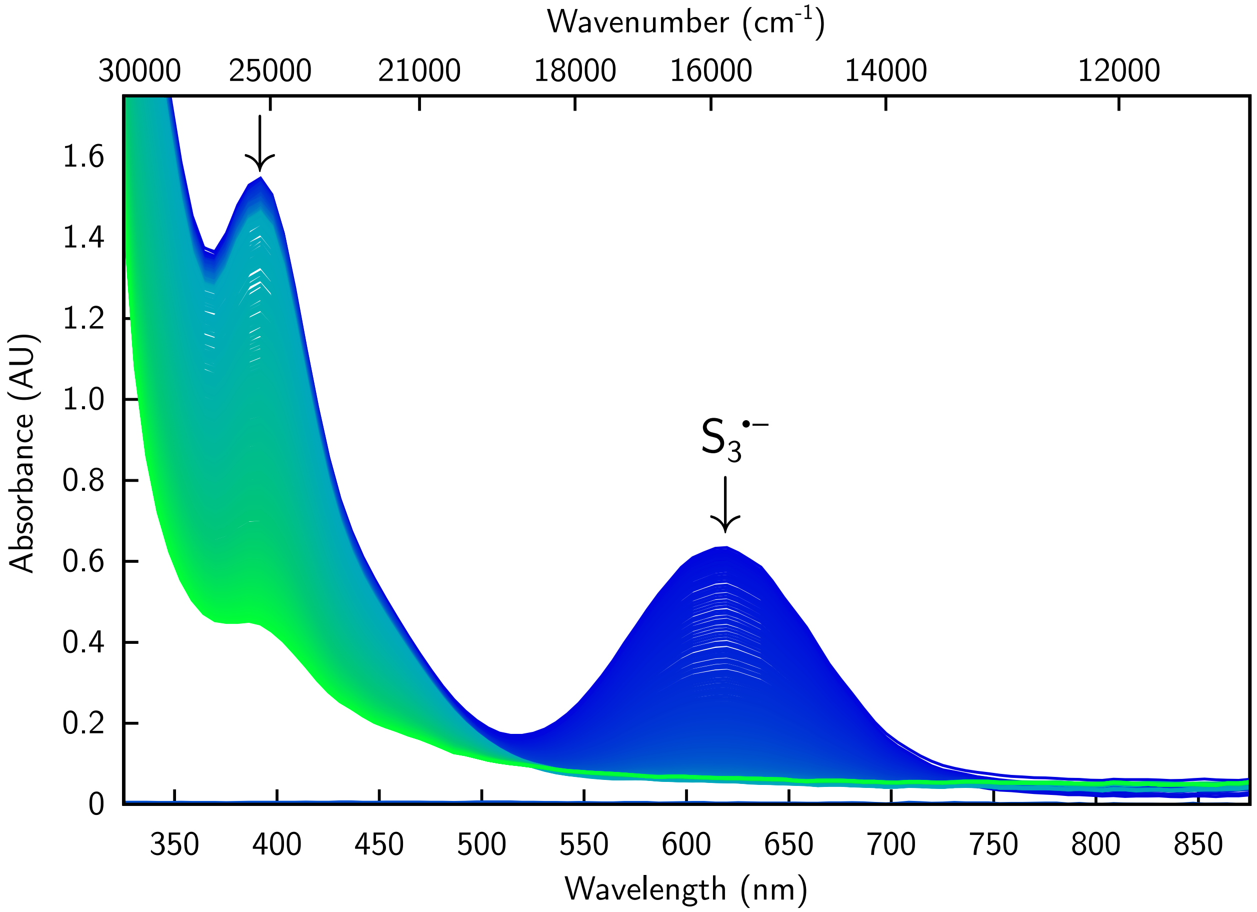

Figure S37: UV-Vis spectra of $[K_2(DMF)_3][\mathbf{4}]$ upon treatment with 6 eq PPh_3 monitored over 10 min at 25 °C (DMF, 0.29 mM).

3.2.3 Treatment of 4 with P(^{t}Bu)$_3$ in DMSO-d_6

To a bright green solution of [K_2(DMF)$_3$][**4**] (7 mg, 0.005 mmol, 1 eq) in DMSO-d_6 (0.5 mL) was added P(^{t}Bu)$_3$ (6 mg, 0.031 mmol, 6.2 eq) as a solid. The reaction mixture gradually became colorless upon complete dissolution of P(^{t}Bu)$_3$ (15 min). The solution was then transferred to an NMR tube and analyzed by ^{1}H (Figure S38), and ^{31}P (Figure S39) NMR spectroscopy after an additional 30 min.

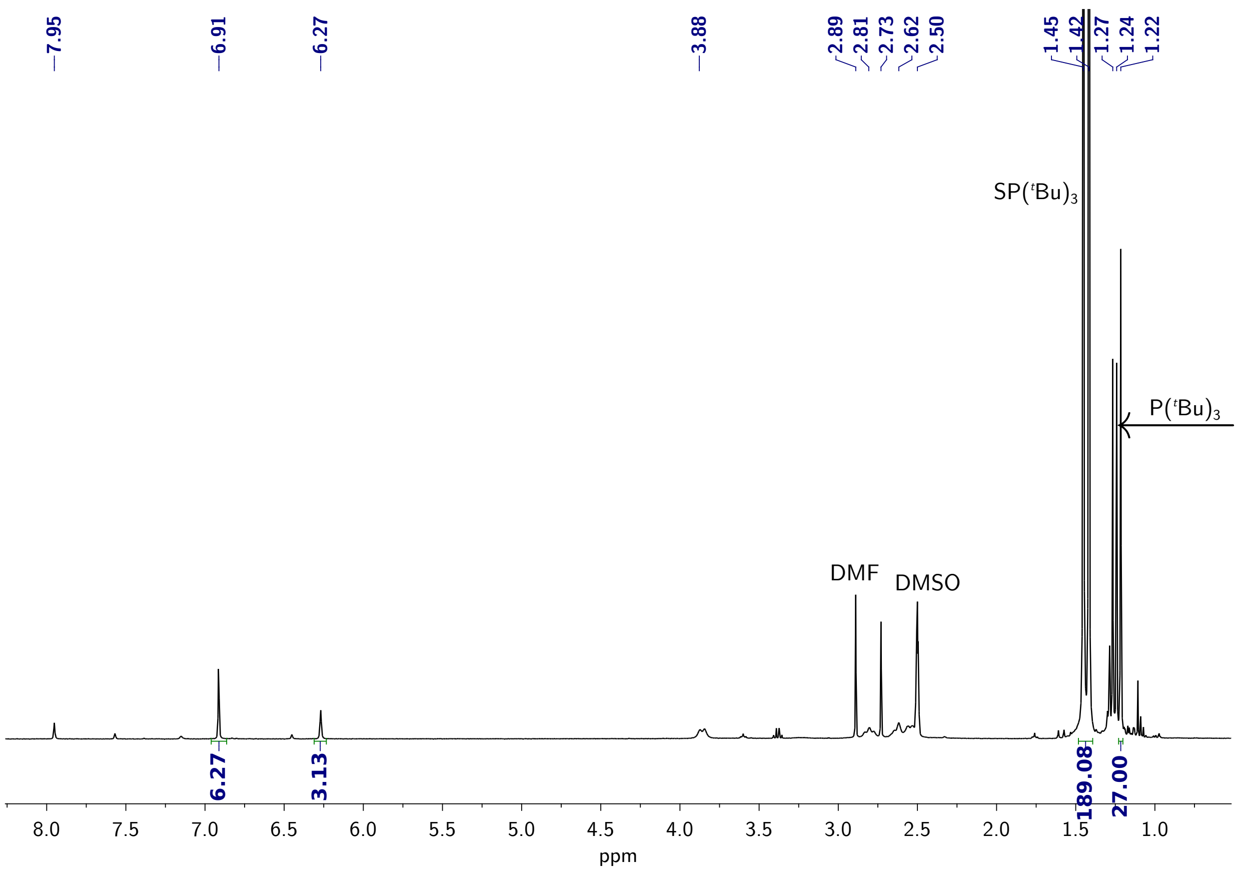

Figure S38: ^{1}H NMR spectrum of **4** treated with 6 eq P(^{t}Bu)$_3$ showing **1** and SP(^{t}Bu)$_3$ (DMSO-d_6, 400 MHz, 25 °C).

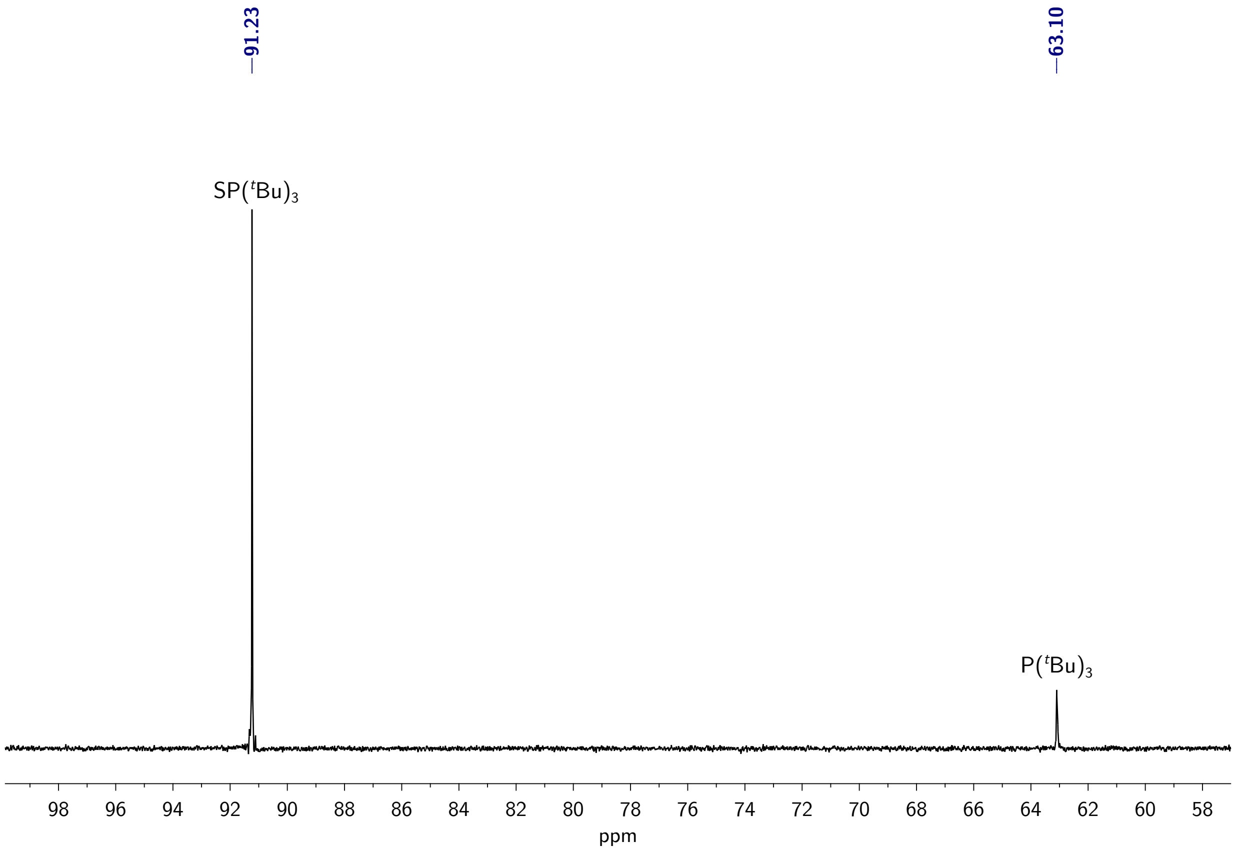

Figure S39: $^{31}P\{^{1}H\}$ NMR spectrum showing $SP(^{t}Bu)_3$ after the treatment of **4** with 6 eq $P(^{t}Bu)_3$ (DMSO-d_6, 162 MHz, 25 °C).

3.2.4 Treatment of 4 with P(O^{i}Pr)$_3$ in DMSO-d_6

To a bright green solution of [K_2(DMF)$_3$][**4**] (7 mg, 0.005 mmol, 1 eq) in DMSO-d_6 (0.5 mL) was added P(O^{i}Pr)$_3$ (8 μL, 0.033 mmol, 6.5 eq) via 10 μL syringe. The reaction mixture immediately became colorless upon complete addition. The solution was then transferred to an NMR tube and analyzed by ^{1}H (Figure S40), ^{31}P (Figure S41) NMR spectroscopy.

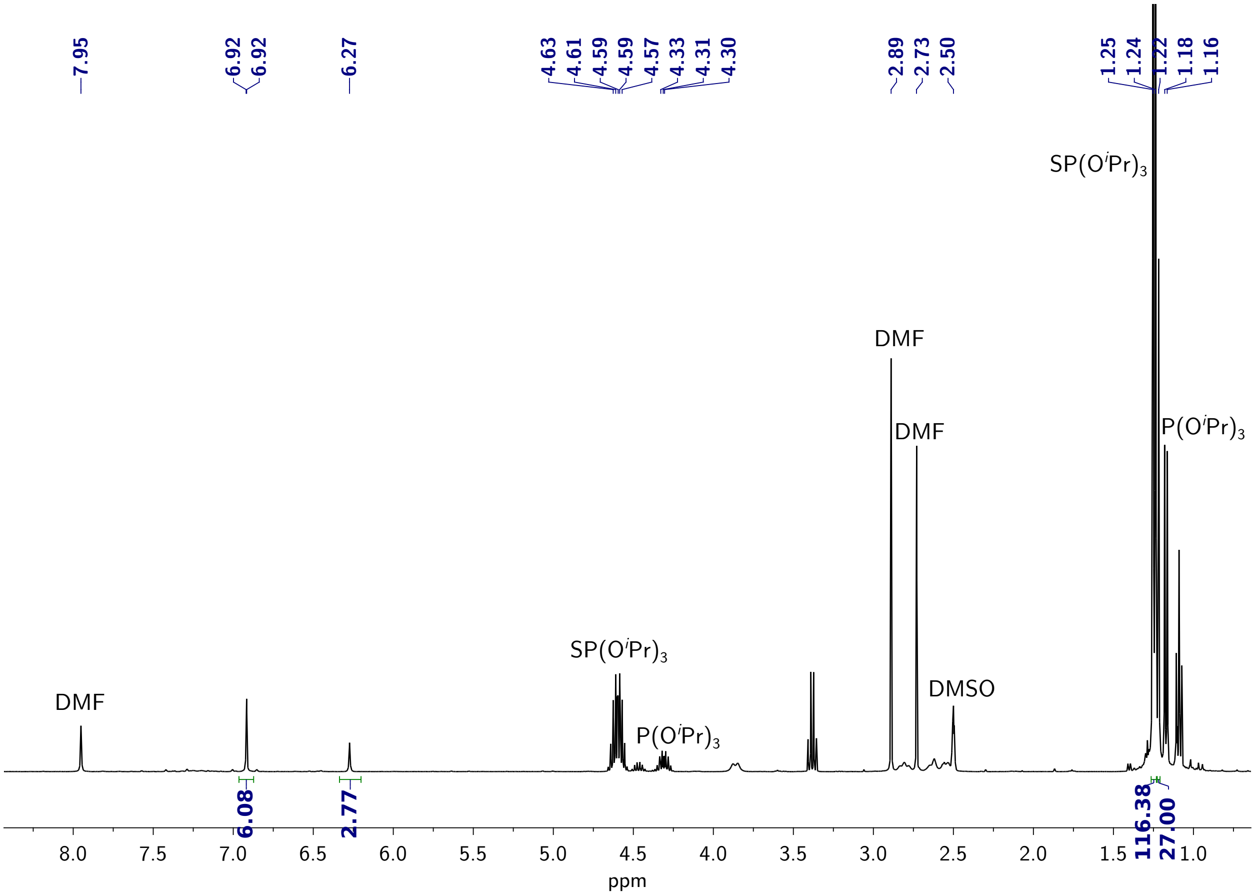

Figure S40: ^{1}H NMR spectrum of **4** treated with P(O^{i}Pr)$_3$ showing **1** and SP(O^{i}Pr)$_3$ (DMSO-d_6, 400 MHz, 25 °C).

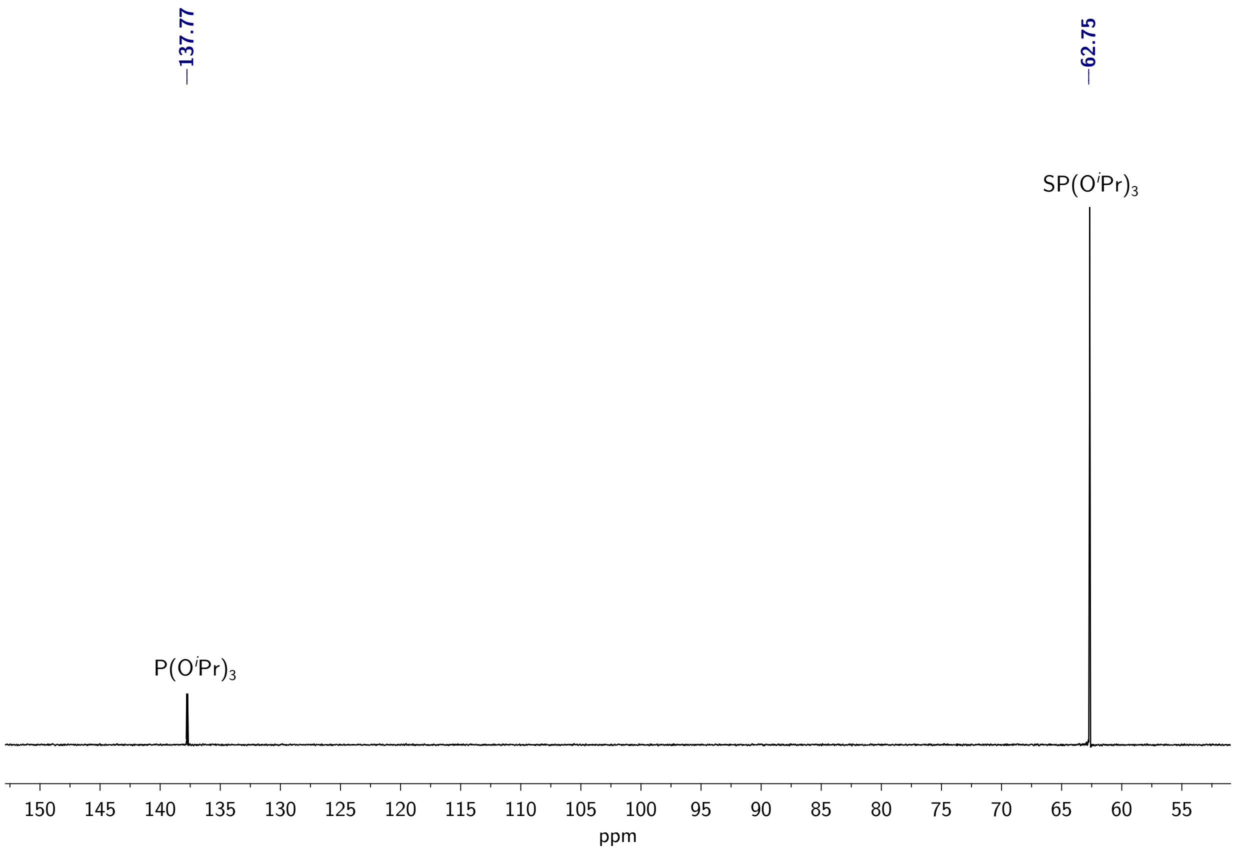

Figure S41: $^{31}P\{^1H\}$ NMR spectrum showing SP(O^{i}Pr)$_3$ after the treatment of **4** with 6 eq P(O^{i}Pr)$_3$ (DMSO-d_6, 162 MHz, 25 °C).

4 Electrochemistry of [K(18-crown-6)]$_2$[**1**]

In a dinitrogen filled glovebox, a 1.25 mM solution of [K(18-crown-6)]$_2$[**1**] was prepared in a 190 mM solution of [TBA][PF_6] in DMF (5 mL total) and transferred to a vial equipped with platinum counter electrode, pseudo-reference electrode (Ag/Ag^+) and a 2 mm^2 glassy carbon working electrode. All cyclic voltammograms were recorded with a BioLogic Science Instruments SP-150 potentiostat using EC Lab V 10.44 software, measured at a scan rate of 100 mV/s, and referenced vs. Fc/Fc^+.

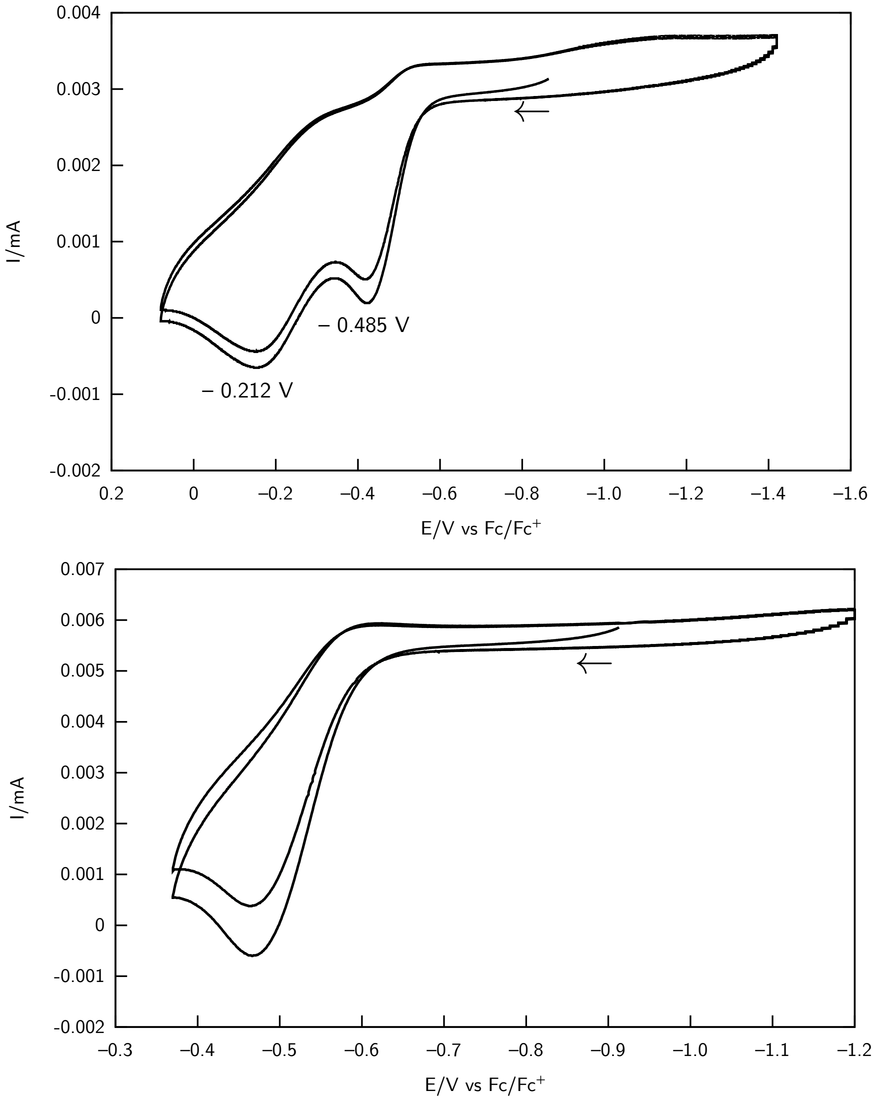

Figure S42: Cyclic voltammograms of a 1.25 mM solution of [K(18-crown-6)]$_2$[**1**] in DMF measured at a scan rate of 100 mV/s and referenced vs. Fc/Fc^+

5 Calculation of the $[(\mu\text{-S}_5)\text{Sn}_2(\mu\text{-S})(m\text{BDCA-5t})]^{2-} \rightleftharpoons 2\text{S}_3^{\bullet-} + [\text{Sn}_2(m\text{BDCA-5t})]$ equilibrium constant

Equilibrium equation:

$$\underset{\mathbf{4}}{[(\mu\text{-S}_5)\text{Sn}_2(\mu\text{-S})(m\text{BDCA-5t})]^{2-}} \rightleftharpoons \underset{\mathbf{Sn_2}}{[\text{Sn}_2(m\text{BDCA-5t})]} + 2\ \text{S}_3^{\bullet-}$$

Assuming $\varepsilon_{\text{S}_3^{\bullet-}} = 4500\ \text{M}^{-1}\text{cm}^{-1}$ [4]

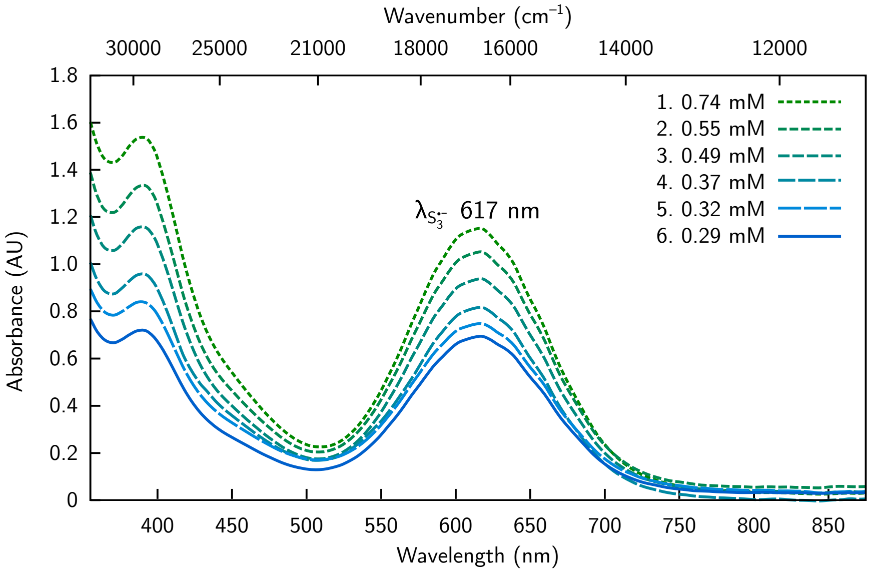

Figure S43: UV-Vis spectra of $[\text{K}_2(\text{DMF})_3][\mathbf{4}]$ collected at varying concentration in DMF solution measured at 25 °C.

$$K_{\text{eq}} = \frac{[\mathbf{S_3}^{\bullet-}]^2[\mathbf{Sn_2}]}{[\mathbf{4}]}$$

Sample	$[\text{K}_2(\text{DMF})_3][\mathbf{4}]$ (mM)	$\text{S}_3^{\bullet-}$ Absorbance	$[\text{S}_3^{\bullet-}]$ (mM)	$[\text{Sn}_2]$ (mM)	K_{eq}	Conversion of **4** to $\text{S}_3^{\bullet-}$ (%)
1	0.74	1.12	0.25	0.12	0.012	17
2	0.55	1.03	0.23	0.11	0.013	21
3	0.49	0.94	0.21	0.10	0.011	21
4	0.37	0.85	0.19	0.094	0.012	26
5	0.32	0.80	0.18	0.090	0.013	28
6	0.29	0.69	0.15	0.077	0.0085	26

Table 1: Parameters used to calculate $K_{\text{eq(avg)}}$.

$K_{\text{eq(avg)}}$ **= 0.012 ± 0.002**

6 Computational details for ^{77}Se and ^{119}Sn NMR calculations of complex 3 and simulated spectra

NMR parameters were computed using both Gaussian 09[5] and Amsterdam Density Functional (ADF) software packages.[6] In Gaussian 09 calculations for magnetic shielding, the B3LYP exchange functional was used. The 6-311++G(d,p) and DGDZVP basis sets employed for H/C/N/Se and Sn atoms, respectively. In the ADF calculations for indirect spin-spin (J) couplings, Vosko-Wilk-Nusair (VWN) exchange-correlation functional[7] was used for the local density approximation (LDA) and Perdew Burke Ernzerhof (PBE) exchange-correlation functional[8] was applied for the generalized gradient approximation (GGA). Standard Slater-type-orbital (STO) basis sets with quadruple-zeta quality plus polarization functions (QZ4P) were used for all the atoms. The spin orbital relativistic effect was incorporated via the zero order regular approximation (ZORA).[9–12] In both Gaussian 09 and ADF calculations, a simplified model containing 76 atoms (Figure S45) was constructed from the crystal structure of **3** to reduce computational cost. All quantum chemical calculations were performed at the High Performance Computing Virtual Laboratory (HPCVL) at Queen's University.

6.1 Computed NMR parameters

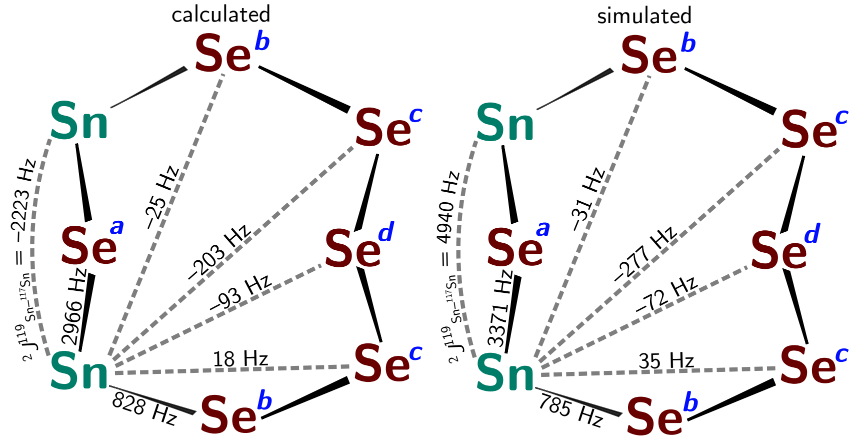

Figure S44: Calculated coupling constants (left), and coupling constants simulated from the experimental spectrum using the program gNMR (right).

	DFT Computation	Experiment
σ_{iso}[Se(a)]/ppm	1732	—
δ_{iso}[Se(a)]/ppm	—	537
σ_{iso}[Se(b)]/ppm	685	—
δ_{iso}[Se(b)]/ppm	—	884
σ_{iso}[Se(c)]/ppm	758	—
δ_{iso}[Se(c)]/ppm	—	789
σ_{iso}[Se(d)]/ppm	601	—
δ_{iso}[Se(d)]/ppm	—	902
$^1J[^{119}\mathrm{Sn}, {}^{77}\mathrm{Se(a)}]$/Hz	2965	3371
$^1J[^{119}\mathrm{Sn}, {}^{77}\mathrm{Se(b)}]$/Hz	828	785
$^2J[^{119}\mathrm{Sn}, {}^{77}\mathrm{Se(c)}]$/Hz	18	Not observed
$^3J[^{119}\mathrm{Sn}, {}^{77}\mathrm{Se(b)}]$/Hz	–25	Not observed
$^3J[^{119}\mathrm{Sn}, {}^{77}\mathrm{Se(d)}]$/Hz	–93	Not observed
$^4J[^{119}\mathrm{Sn}, {}^{77}\mathrm{Se(c)}]$/Hz	–203	277^a
$^2J[^{119}\mathrm{Sn}, {}^{117}\mathrm{Sn}]$/Hz	–2223	4940^a
$^1J[^{77}\mathrm{Se}, {}^{77}\mathrm{Se}]$/Hz	<65^b	Not observed

Table 2: Computed NMR parameters for the model shown below and the corresponding experimental data for **3**. Since the computational model shown above is asymmetric, Sn(1)/Sn(2), Se(2)/Se(6), and Se(4)/Se(5) exhibit slightly different NMR parameters. The reported computational magnetic shielding and J-coupling constant values for Se(a), Se(b), Se(c), and Se(d) are the average values. ^{a} The sign of J was not determined. ^{b}All $^1J(^{77}\mathrm{Se},^{77}\mathrm{Se})$ coupling constants in the molecule are less than 65 Hz.

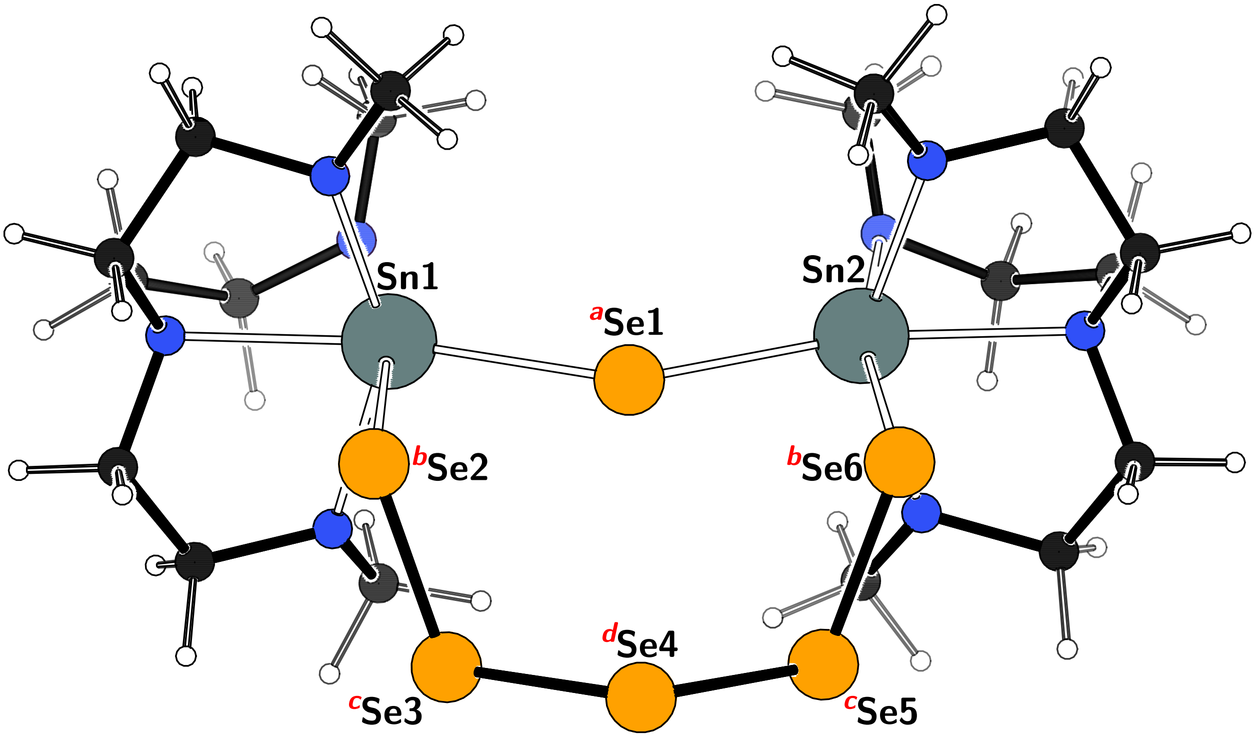

Figure S45: Truncated model of complex **3** containing 76 atoms used for NMR calculations. Coordinates for the model system were derived from the X-ray crystallographic coordinates experimentally obtained for complex **3** by replacing the three carboxamide aryl spacers with methyl groups.

6.1.1 X, Y, Z coordinates of truncated model complex (Figure S45)

Sn	1.177568	-0.068398	1.710459
Sn	2.206496	-3.033046	5.515574
Se	2.455994	-1.552019	3.387497
Se	3.746322	0.213720	0.842398
Se	4.151070	-1.952768	0.083106
Se	5.808004	-2.814136	1.490534
Se	5.034580	-4.274402	3.140716
Se	4.886106	-3.048245	5.109146
N	-1.046344	0.000000	1.997823
N	0.097041	-3.293847	6.221750
C	-1.634536	0.176092	0.652016
C	-1.009690	1.338299	-0.120854
C	0.054785	-4.667365	6.739484
C	1.087535	-4.845310	7.855519
C	-1.489433	0.759976	2.940784
C	-0.367079	-2.265099	6.857354
N	1.364390	2.044521	2.432003

N	2.847679	-1.699751	7.212456
C	0.754039	3.041758	1.503963
C	1.063710	2.608015	0.076093
C	2.656506	-2.392998	8.520963
C	3.230322	-3.799880	8.388173
C	2.062145	2.567236	3.401820
C	3.051123	-0.418914	7.351214
N	0.773751	-1.392054	0.046253
N	2.186012	-5.112229	4.655123
C	0.684485	-0.795194	-1.408473
C	0.999338	0.685832	-1.342824
C	2.645604	-6.187317	5.565259
C	3.147141	-5.672016	6.891670
C	0.357199	-2.615430	-0.091014
C	1.328795	-5.644212	3.807652
N	0.482738	1.266009	-0.099966
N	2.418598	-4.482005	7.343754
H	-1.325534	2.276421	0.333118
H	-1.351936	1.301661	-1.154027
H	-2.701620	0.364361	0.760681
H	-1.483675	-0.740581	0.083796
H	0.537941	1.188243	-2.191716
H	2.078741	0.824773	-1.381605
H	-0.322909	-0.933514	-1.798275
H	1.399056	-1.287706	-2.066304
H	2.141019	2.572822	-0.079108
H	0.617541	3.304876	-0.631889
H	1.177262	4.027630	1.690668
H	-0.324000	3.075078	1.654437
H	1.595637	-2.446204	8.761060
H	3.180002	-1.851583	9.307565
H	3.150391	-4.331224	9.335382
H	4.275138	-3.754416	8.084505
H	4.201625	-5.418976	6.791896
H	3.031177	-6.456849	7.637666
H	3.453447	-6.731099	5.077805
H	1.812345	-6.863722	5.749854
H	1.094197	-5.883645	8.183754
H	0.831504	-4.200817	8.695160
H	0.275451	-5.363090	5.931280
H	-0.939416	-4.872471	7.133713
H	1.332295	-5.072449	2.880831
H	1.615357	-6.674090	3.599913
H	2.130654	0.120577	7.133021
H	3.361022	-0.209283	8.373926

H	0.330233	-5.625787	4.241766
H	-0.348233	-1.395065	6.202673
H	0.248438	-2.072508	7.734832
H	-1.391651	-2.462803	7.168940
H	3.831555	-0.098621	6.662550
H	3.120882	2.379400	3.229446
H	1.762974	2.117660	4.347494
H	1.885251	3.641151	3.438384
H	-0.619251	-2.684111	0.386217
H	1.063086	-3.258625	0.432383
H	0.276964	-2.933816	-1.129336
H	-1.186051	1.788476	2.750867
H	-1.079993	0.431003	3.894753
H	-2.576422	0.702210	2.973015

6.2 Experimental and simulated spectra

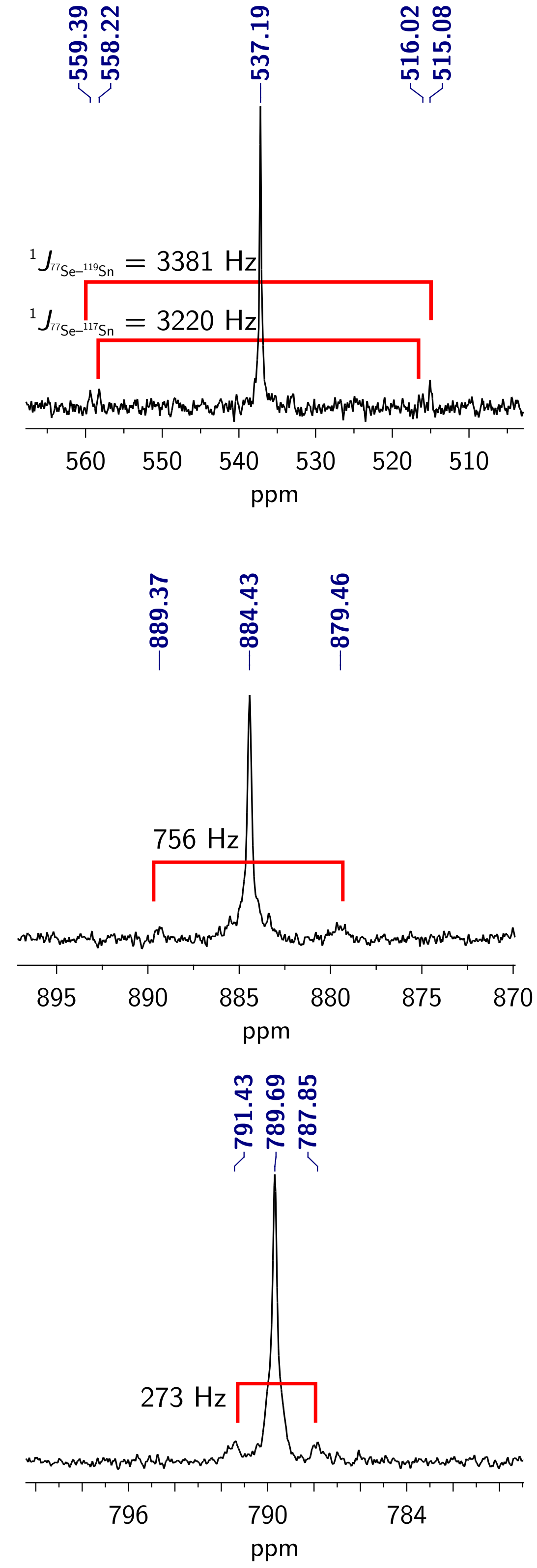

Figure S46: Zoomed-in ^{77}Se NMR spectra of [$K_2(DMF)_3$][**3**] showing ^{119}Sn–^{77}Se and ^{117}Sn–^{77}Se coupling (DMSO-d_6, 76.3 MHz, 25 °C).

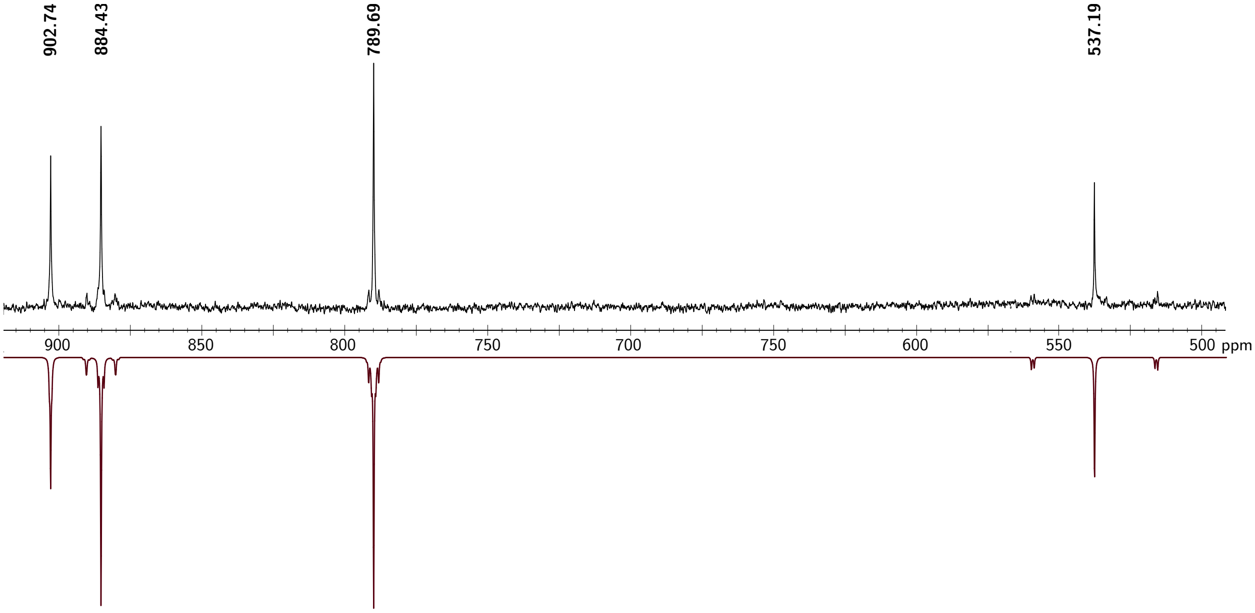

Figure S47: Top: Experimental ^{77}Se NMR spectrum of [K_2(DMF)$_3$][**3**] (DMSO-d_6, 76.3 MHz, 25 °C); Bottom: simulated spectrum using the program gNMR.

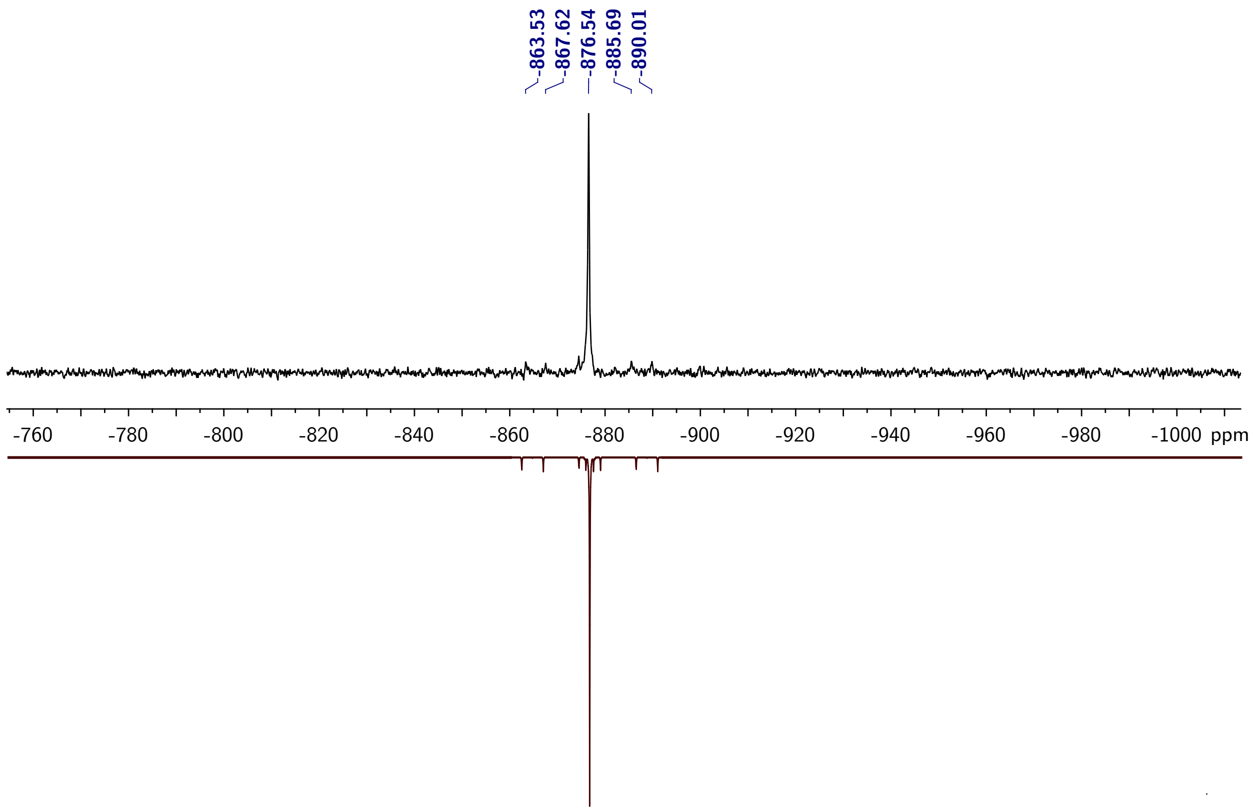

Figure S48: Top: Zoomed-in $^{119}Sn\{^{1}H\}$ NMR spectrum of $[K_2(DMF)_3][\mathbf{3}]$ showing ^{119}Sn–^{77}Se coupling (DMSO-d_6, 186.38 MHz, 25 °C); Bottom: simulated spectrum using the program gNMR.

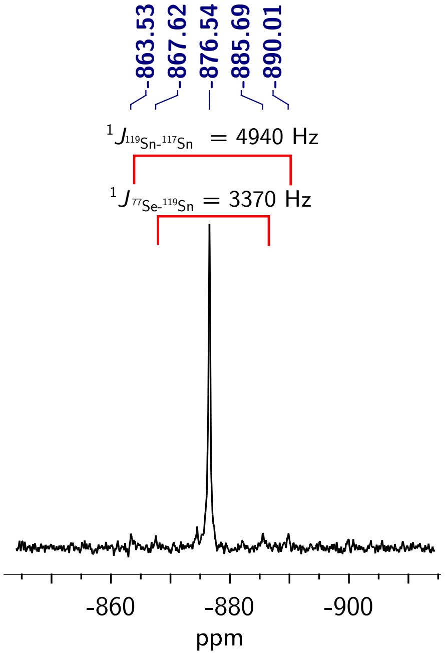

Figure S49: Zoomed-in ^{119}Sn{^{1}H} NMR spectrum of [K_2(DMF)$_3$][**3**] (DMSO-d_6, 186.38 MHz, 25 °C) showing ^{119}Sn-^{77}Se coupling ($^{1}J_{^{119}Sn-^{77}Se}$ = 3370 Hz) and ^{119}Sn-^{117}Sn coupling ($^{2}J_{^{119}Sn-^{77}Se}$ = 4940 Hz).

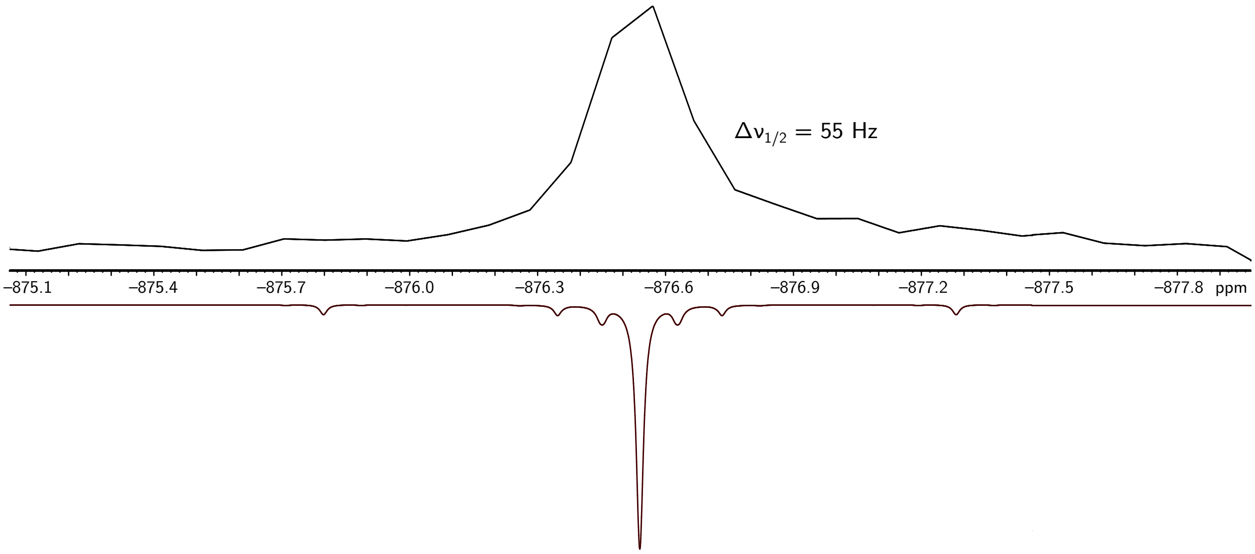

Figure S50: Top: zoomed-in ^{119}Sn{^{1}H} NMR spectrum of [K_2(DMF)$_3$][**3**] (DMSO-d_6, 186.38 MHz, 25 °C); Bottom: simulated spectrum. The line width of [K_2(DMF)$_3$][**3**] ($\Delta\nu_{1/2}$ = 55 Hz) prevents the observation of higher order ^{119}Sn-^{77}Se coupling including the $^{4}J_{^{119}Sn-^{77}Se}$ = 273 Hz coupling observed in the ^{77}Se NMR spectrum (Figure S46).

7 Computational details for model complex, $[Sn_2(m\text{BDCA-5H})]^{2-}$

Coordinates for the model system $[Sn_2(m\text{BDCA-5H})]^{2-}$ were derived from the X-ray crystallographic coordinates experimentally obtained for $[Sn_2(m\text{BDCA-5t})]^{2-}$ by replacing the three spacer *tert*-butyl groups with H atoms, and then by fixing the coordinates of the Sn_2N_8 core while optimizing the coordinates for all C, H, and O atoms at the B3LYP-D3/defbas-4 level of theory. After the optimization, the electronic structure of $[Sn_2(m\text{BDCA-5H})]^{2-}$ was calculated at the level of theory indicated in the Figure 2 caption in the main text.

7.1 X, Y, Z coordinates of model complex, $[Sn_2(m\text{BDCA-5H})]^{2-}$

Sn	4.28670493936618	-2.57743903430370	0.91762997102650
Sn	2.21131097270645	-2.61556693985125	3.78197887772457
N	6.31620803040689	-3.53622798336932	1.11218097755892
N	3.50584704556292	-3.60229496192599	-0.93059598374688
N	6.11260196851951	-2.53655202189370	-1.60551195982352
N	4.84263902805418	-0.55946999554498	0.08411498288721
N	0.38976598371475	-2.65120297457617	6.30834395589906
N	0.22597501322229	-3.66372301202991	3.59271405900703
N	1.56704399577495	-0.61964602626678	4.60491905091809
N	3.04061203007942	-3.59640605052779	5.63298507012743
C	0.12375737820247	0.14516464001230	0.15130625616967
C	0.00238208068243	0.17545893913503	1.53423069480211
C	1.13794788213821	0.17277151204123	2.34285312465783
C	2.39809794270464	0.15143111765575	1.74195910415524
C	2.53030689428294	0.16075946227009	0.35083079918722
C	1.38039497174105	0.16121646584672	-0.43882144611136
C	0.94821293653726	0.29448985302387	3.83718830342578
O	0.23895153364045	1.22539908128275	4.25627399978756
C	3.87459318254520	0.28174926876693	-0.33053587684881
O	3.99862661062242	1.14995853031376	-1.21157057886110
C	1.39740619796776	-0.44044185692105	6.04280565611565
C	0.19489983525644	-1.23251734377053	6.54727657603032
C	-0.77488723975458	-3.37556193730086	5.82990967060777
C	-0.94508732534707	-3.19419274872665	4.32104251937664
C	0.23076980410512	-4.97915151019274	3.29386460268317
C	1.40786204195892	-5.47549952959127	2.48128800992826
C	1.78147692251014	-4.89624062745646	1.26576825006380
C	2.81183968471093	-5.44196232780575	0.49512370381460

C	3.49814676384603	-6.55656346466262	0.97626422961030
C	3.16168136698621	-7.11210611747595	2.20439886621658
C	2.10264449862206	-6.59411132852053	2.93826813442113
C	3.16871361767630	-4.90373883804018	-0.87529458267153
O	3.13400513758572	-5.68120041762524	-1.84378563361313
C	2.17370037717143	-4.30661978479930	6.56425191540872
C	1.21395159001301	-3.35329095474522	7.27403947088818
C	4.15631582164513	-3.06364310763209	6.17088106580257
C	5.11243185741266	-2.38686281571977	5.21000547881962
C	5.58222728294149	-3.00204150514112	4.04564170064637
C	6.54904733330152	-2.38667301043630	3.24536311017962
C	7.02213734901416	-1.12556649243624	3.60650351257654
C	6.52962337648144	-0.48879028951826	4.73817283027173
C	5.59640712426435	-1.12365599544926	5.54695042867135
C	7.16953758367529	-3.06235897669451	2.03973515458100
O	8.40933396311901	-3.12758388003682	1.98323240155553
O	-0.63704895533758	-5.80300048629982	3.63134289815793
O	4.45801151458359	-3.08056983160831	7.37686742072290
C	6.15504571348909	-0.34870638574750	-0.51403722776488
C	6.29111750528662	-1.11385456186694	-1.82859758958030
C	5.29732312706911	-3.23896031293330	-2.57994503676049
C	3.81120107413497	-3.08656802572284	-2.26027616053379
C	6.92284589441147	-4.22756352258597	-0.01604873445833
C	7.28704488698652	-3.25375798532873	-1.13662223724496
H	1.25411030475107	-4.02236216024391	0.90717040238574
H	1.80294352195277	-7.04438351573869	3.87513262706712
H	3.72073618388661	-7.95796154427396	2.58712322533746
H	4.29598636476349	-6.97850646614847	0.37887560336294
H	5.20773727144358	-3.97927203449393	3.77304582943268
H	5.23179835206658	-0.65378906100076	6.45060647719563
H	6.88602001381247	0.50049586258803	5.00061391300729
H	7.77851490700668	-0.65699189034448	2.99025199997368
H	3.28520836398989	0.16337993928692	2.36086299865800
H	-0.97143090887038	0.21394232492796	2.00462191358284
H	-0.76489220882367	0.12668583717088	-0.46914266767009
H	1.48954585610149	0.19123922793706	-1.51518828355868
H	5.53468668784258	-4.30252512713536	-2.53722807301860
H	5.50779294634670	-2.89732586409171	-3.60895682274660
H	3.53449906488556	-2.02880218456999	-2.29414860578937
H	3.22584330409068	-3.62200170633802	-3.01102265807113
H	5.51105159170789	-0.75893452586136	-2.50190044410326
H	7.26299384087526	-0.89341677908510	-2.30612539730763
H	6.91365094994615	-0.69837749929019	0.19209046638967
H	6.31917773089308	0.71627137755031	-0.69806938875332
H	6.20684802594382	-4.96406401899920	-0.39190368224136

H	7.82281837720959	-4.75668749200234	0.30852103529941
H	8.00229141084479	-2.53635484304438	-0.73350098237290
H	7.78787663779918	-3.79469724548225	-1.95920003745112
H	0.00302681366496	-1.01790893879810	7.61396306556365
H	-0.67782743749675	-0.89445066039716	5.98830752705418
H	1.25599760884977	0.61713699613649	6.27587517137714
H	2.30106468377728	-0.79496987922268	6.54605851576234
H	-1.69258021914909	-3.06636923904284	6.36196963842599
H	-0.63567716064827	-4.43988249429207	6.01854835236634
H	-1.83152433367212	-3.74299730360965	3.99276594364627
H	-1.08978858081121	-2.13509461081996	4.08957198326227
H	0.59914134145431	-3.90515296410823	8.00788820355056
H	1.81284818213653	-2.63039592118340	7.82779557098106
H	2.77417226309818	-4.83443594599352	7.31004390637222
H	1.59806878100548	-5.04373789087106	5.99805968622089

8 Crystallographic information for [K(Kryptofix-2,2,2)]$_2$[1], [K(Kryptofix-2,2,2)]$_2$[2], and [K$_2$(DMF)$_3$][3]

8.1 X-ray crystal structure determination details

Low-temperature (100 K) diffraction data (ϕ and ω) were collected on a Bruker-AXS X8 Kappa Duo diffractometer coupled to a Smart APEX2 CCD detector with Mo Kα radiation (λ = 0.71073 Å) from an IμS micro-source. Absorption and other corrections were applied using SADABS.[13] The structures were solved by direct methods using SHELXT[14] and refined against F^2 on all data by full-matrix least squares with SHELXL-2015[15] using established refinement approaches.[16] All hydrogen atoms were included into the model at geometrically calculated positions and refined using a riding model. The isotropic displacement parameters of all hydrogen atoms were fixed to 1.2 times the U_{eq} value of the atoms they are linked to (1.5 times for methyl groups). Details about crystal properties, diffraction data and crystal structures can be found in the tables below. All disorders were refined with the help of similarity restraints on 1,2- and 1,3-distances as well as similar ADP and advanced rigid bond restraints.[17]

[K$_2$(DMF)$_3$][3] crystallizes in the monoclinic chiral space group $P2_1$ with one target molecule, two potassium ions and two partially occupied water molecules in the asymmetric unit. The two water molecules are bridging the potassium ions, giving rise to a four-membered ring. This K_2O_2 ring crosslinks the target molecules into infinite two-dimensional sheets coinciding with the [1 0 1] planes. The structure was refined as an inversion twin; the twin ratio converged at 0.27(2). The structure was challenging to refine and is dominated by significant disorder: the entire cryptand ligand is disordered over two positions and, independently, the six Se atoms of the eight-membered Sn_2Se_6-ring are also disordered over two positions. The disorder ratios were refined freely and converged at 0.583(15) for the ligand disorder and at 0.893(3) for the disordered Se atoms. The program SQUEEZE[18] as implemented in PLATON[19] was used to account for the contribution of disordered solvent contained in voids within the crystal lattice. The solvent contribution was added to the model in a separate file (the .fab file) by SHELXL. Squeeze identified one crystallographi-

cally independent solvent accessible void, located at coordinates 0.5, 0.5, 0.0 with a volume of 836 Å^3, which is enough space for ca. 40 non-hydrogen atoms or ca. 8 DMF molecules (the crystals were grown from DMF). In this void, Squeeze identified the equivalent of 286 electrons, corresponding to about 7 DMF molecules. The hydrogen atoms on the two partially occupied bridging water molecules could not be located in the difference Fourier synthesis and were, therefore, not included into the crystallographic model.

Symmetry transformations used to generate equivalent atoms:

#1 -x+1,y-1/2,-z+1 #2 -x,y-1/2,-z #3 x,y-1,z #4 -x,y+1/2,-z #5 -x+1,y+1/2,-z+1 #6 x,y+1,z

[K(Kryptofix-2,2,2)]$_2$[2], and **[K(Kryptofix-2,2,2)]$_2$[1]** are isostructural (the only difference is in the metal atoms, Pb in [K(Kryptofix-2,2,2)]$_2$[**2**] and Sn in [K(Kryptofix-2,2,2)]$_2$[**1**]) and the two respective refinements were identical. Both compounds crystallize in the hexagonal centrosymmetric space group $P6_3/m$ with one sixth of a target molecule and one sixth of a Kryptofix-2,2,2 coordinated potassium ion per asymmetric unit. The one half crystallographically independent *t*-butylaryl moiety of the ligand is disordered over two positions, corresponding to four effective disorder components when considering the crystallographic mirror symmetry. The ratio between the two independent disorder components was refined freely and converged at 0.770(6) for the structure of [K(Kryptofix-2,2,2)]$_2$[**2**] and 0.778(5) for the structure of [K(Kryptofix-2,2,2)]$_2$[**1**]. In addition, the potassium ion was refined as disordered over two positions. The K-disorder ratio refined 0.95(2) for the structure of [K(Kryptofix-2,2,2)]$_2$[**2**] and 0.87(4) for the structure of [K(Kryptofix-2,2,2)]$_2$[**1**]. This is only barely significant for the structure of [K(Kryptofix-2,2,2)]$_2$[**2**]; however introduction of the disorder markedly improved the structure.

Symmetry transformations used to generate equivalent atoms for [K(Kryptofix-2,2,2)]$_2$[**2**]:

#1 -x+y-1,-x+1,z #2 -y+1,x-y+2,z #3 x,y,-z+1/2 #4 -x+y,-x+1,z #5 -y+1,x-y+1,z Symmetry transformations used to generate equivalent atoms for [K(Kryptofix-2,2,2)]$_2$[**1**]:

#1 -y+1,x-y+2,z #2 -x+y-1,-x+1,z #3 -y+1,x-y+1,z #4 -x+y,-x+1,z

8.2 Solid-state structure of [K(Kryptofix-2,2,2)]$_2$[2]

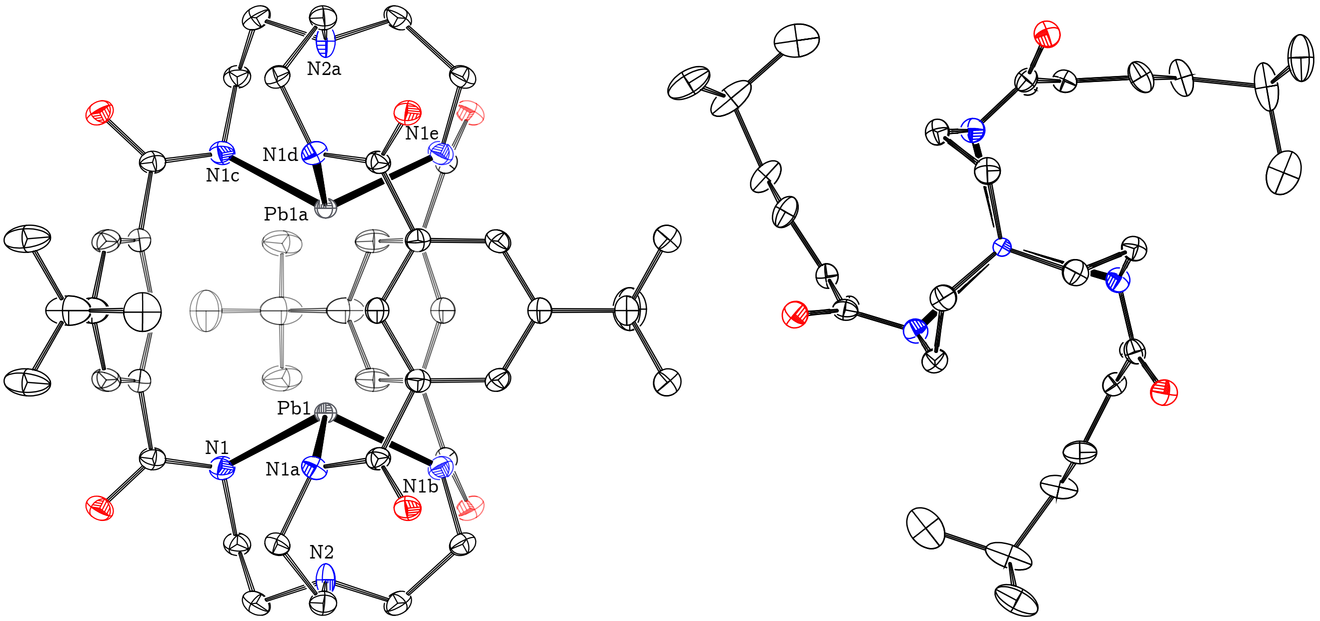

Figure S51: Solid-state structure of $[Pb_2(m\text{BDCA-5t})]^{2-}$ (**2**) with thermal ellipsoids (Drawn using PLATON[19]) shown at the 50% probability level (left), and view down the Pb···Pb axis (right). $[K(\text{Kryptofix } 2,2,2)]^+$ cations, H-atoms and disorder omitted for clarity. Selected interatomic distances (Å) and angles (deg): Pb1–N1 2.380(2), Pb1–N2 2.856(4), Pb1–Pb1a 3.5274(5), N1–Pb1–N1a 105.26(6).

Table 3: Crystallographic Data for [K(Kryptofix-2,2,2)]$_2$[**1**], [K(Kryptofix-2,2,2)]$_2$[**2**], and [K$_2$(DMF)$_3$][**3**]

	[K(Kryptofix-2,2,2)]$_2$[**1**]	[K(Kryptofix-2,2,2)]$_2$[**2**]	[K$_2$(DMF)$_3$][**3**]
Reciprocal Net code / CCDC	X8_15178 / 1469595	X8_15162 / 1469593	X8_13160 / 1469596
Empirical formula, FW (g/mol)	$C_{84}H_{132}K_2N_{12}O_{18}Sn_2$, 1913.59	$C_{84}H_{132}K_2N_{12}O_{18}Pb_2$, 2090.59	$C_{48}H_{60}K_2N_8O_{7.5}Se_6Sn_2$, 1658.38
Color / Morphology	Pale yellow / Block	Colorless / Block	Red / Block
Crystal size (mm^3)	$0.257 \times 0.177 \times 0.086$	$0.030 \times 0.020 \times 0.010$	$0.180 \times 0.130 \times 0.030$
Temperature (K)	100(2)	100(2)	100(2)
Wavelength (Å)	0.71073	0.71073	0.71073
Crystal system, Space group	Hexagonal, P6$_3$/*m*	Hexagonal, P6$_3$/*m*	Monoclinic, P2$_1$/*c*
Unit cell dimensions (Å, °)	$a = 13.1963(19)$, $\alpha = 90$	$a = 13.2463(16)$, $\alpha = 90$	$a = 13.2183(19)$, $\alpha = 90$
	$b = 13.1963(19)$, $\beta = 90$	$b = 13.2463(16)$, $\beta = 90$	$b = 18.536(3)$, $\beta = 113.704(2)$
	$c = 29.806(5)$, $\gamma = 120$	$c = 29.783(4)$, $\gamma = 120$	$c = 16.295(2)$, $\gamma = 90$
Volume (Å^3)	4495.0(15)	4525.7(12)	3655.7(9)
Z	2	2	2
Density (calc., g/cm^3)	1.420	1.540	1.507
Absorption coefficient (mm^{-1})	0.723	3.881	3.828
$F(000)$	2012	2140	1612
Theta range for data collection (°)	1.366 to 31.504	1.367 to 31.502	1.365 to 26.372
Index ranges	$-19 \leq h \leq 19$, $-19 \leq k \leq 19$, $-43 \leq l \leq 43$	$-19 \leq h \leq 19$, $-19 \leq k \leq 18$, $-43 \leq l \leq 43$	$-16 \leq h \leq 16$, $-23 \leq k \leq 23$ $-20 \leq l \leq 20$
Reflections collected	186032	113659	62975
Independent reflections, R_{int}	5098, 0.0604	5130, 0.0853	14765, 0.0807
Completeness to θ_{max} (%)	100.0	100.0	99.9
Absorption correction	Semi-empirical from equiv.	Semi-empirical from equiv.	Semi-empirical from equiv.
Refinement method	Full-matrix least-squares on F^2	Full-matrix least-squares on F^2	Full-matrix least-squares on F^2
Data / Restraints / Parameters	5098 / 546 / 274	5130 / 546 / 274	14765 / 5504 / 1264
Goodness-of-fit^a	1.047	1.014	1.035
Final R indices^b [$I > 2\sigma(I)$]	$R_1 = 0.0372$, $wR_2 = 0.0931$	$R_1 = 0.0267$, $wR_2 = 0.0517$	$R_1 = 0.0718$, $wR_2 = 0.1873$
R indices^b (all data)	$R_1 = 0.0481$, $wR_2 = 0.1008$	$R_1 = 0.0532$, $wR_2 = 0.0612$	$R_1 = 0.1017$, $wR_2 = 0.2100$
Largest diff. peak and hole ($e \cdot$Å^{-3})	1.307 and -1.035	3.298 and -1.673	1.928 and -1.128

^{a} GooF = $\sqrt{\frac{\Sigma[w(F_o^2-F_c^2)^2]}{(n-p)}}$ ^{b} $R_1 = \frac{\Sigma||F_o|-|F_c||}{\Sigma|F_o|}$; $wR_2 = \sqrt{\frac{\Sigma[w(F_o^2-F_c^2)^2}{\Sigma[w(F_o^2)^2]}}$; $w = \frac{1}{\sigma^2(F_o^2)+(aP)^2+bP}$; $P = \frac{2F_c^2+\max(F_o^2,0)}{3}$

References

(1) Lopez, N.; Graham, D. J.; McGuire, R.; Alliger, G. E.; Shao-Horn, Y.; Cummins, C. C.; Nocera, D. G. *Science* **2012**, *335*, 450–453.

(2) Gynane, M. J. S.; Harris, D. H.; Lappert, M. F.; Power, P. P.; Riviere, P.; Riviere-Baudet, M. *J. Chem. Soc., Dalton Trans.* **1977**, 2004–2009.

(3) Gabuda, S.; Kozlova, S.; Terskikh, V.; Dybowski, C.; Neue, G.; Perry, D. *Chem. Phys. Lett.* **1999**, *305*, 353–358.

(4) Chivers, T.; Elder, P. J. W. *Chem. Soc. Rev.* **2013**, *42*, 5996–6005.

(5) Frisch, M. J. et al. *Gaussian 09 Revision E.01*, Gaussian Inc. Wallingford CT 2009.

(6) te Velde, G.; Bickelhaupt, F. M.; Baerends, E. J.; Fonseca Guerra, C.; van Gisbergen, S. J. A.; Snijders, J. G.; Ziegler, T. *J. Comput. Chem.* **2001**, *22*, 931–967.

(7) Vosko, S. H.; Wilk, L.; Nusair, M. *Can. J. Phys.* **1980**, *58*, 1200–1211.

(8) Perdew, J. P.; Burke, K.; Ernzerhof, M. *Phys. Rev. Lett.* **1996**, *77*, 3865–3868.

(9) Lenthe, E. v.; Baerends, E. J.; Snijders, J. G. *J. Chem. Phys.* **1993**, *99*, 4597–4610.

(10) van Lenthe, E.; Baerends, E. J.; Snijders, J. G. *J. Chem. Phys.* **1994**, *101*, 9783–9792.

(11) van Lenthe, E.; Snijders, J. G.; Baerends, E. J. *J. Chem. Phys.* **1996**, *105*, 6505–6516.

(12) van Lenthe, E.; van Leeuwen, R.; Baerends, E. J.; Snijders, J. G. *Int. J. Quantum Chem.* **1996**, *57*, 281–293.

(13) Krause, L.; Herbst-Irmer, R.; Sheldrick, G. M.; Stalke, D. *J. Appl. Cryst.* **2015**, *48*, 3–10.

(14) Sheldrick, G. M. *Acta Crystallogr. A* **2015**, *71*, 3–8.

(15) Sheldrick, G. M. *Acta Crystallogr. C* **2015**, *71*, 3–8.

(16) Müller, P. *Crystallography Reviews* **2009**, *15*, 57–83.

(17) Thorn, A.; Dittrich, B.; Sheldrick, G. M. *Acta Crystallogr. A* **2012**, *68*, 448–451.

(18) van der Sluis, P.; Spek, A. L. *Acta Crystallogr. A* **1990**, *46*, 194–201.

(19) Spek, A. L. *Acta Crystallogr. D* **2009**, *65*, 148–155.
